# Transcriptome-Wide Identification and Characterization of MicroRNAs from Castor Bean (*Ricinus communis* L.)

**DOI:** 10.1371/journal.pone.0069995

**Published:** 2013-07-24

**Authors:** Wei Xu, Qinghua Cui, Fei Li, Aizhong Liu

**Affiliations:** 1 Kunming Institute of Botany, Chinese Academy of Sciences, Kunming, China; 2 College of Life Sciences, Yunnan University, Kunming, China; 3 Key Laboratory of Tropical Plant Resource Science, Xishuangbanna Tropical Botanical Garden, Chinese Academy of Sciences, Kunming, China; Cankiri Karatekin University, Turkey

## Abstract

**Background:**

MicroRNAs (miRNAs) are endogenously encoded small RNAs that post-transcriptionally regulate gene expression and play essential roles in numerous developmental and physiological processes. Currently, little information on the transcriptome and tissue-specific expression of miRNAs is available in the model non-edible oilseed crop castor bean (*Ricinus communis* L.), one of the most important non-edible oilseed crops cultivated worldwide. Recent advances in sequencing technologies have allowed the identification of conserved and novel miRNAs in many plant species. Here, we used high-throughput sequencing technologies to identify and characterize the miRNAs in castor bean.

**Results:**

Five small RNA libraries were constructed for deep sequencing from root tips, leaves, developing seeds (at the initial stage, seed1; and at the fast oil accumulation stage, seed2) and endosperms in castor bean. High-throughput sequencing generated a large number of sequence reads of small RNAs in this study. In total, 86 conserved miRNAs were identified, including 63 known and 23 newly identified. Sixteen miRNA isoform variants in length were found from the conserved miRNAs of castor bean. MiRNAs displayed diverse organ-specific expression levels among five libraries. Combined with criteria for miRNA annotation and a RT-PCR approach, 72 novel miRNAs and their potential precursors were annotated and 20 miRNAs newly identified were validated. In addition, new target candidates for miRNAs newly identified in this study were proposed.

**Conclusions:**

The current study presents the first high-throughput small RNA sequencing study performed in castor bean to identify its miRNA population. It characterizes and increases the number of miRNAs and their isoforms identified in castor bean. The miRNA expression analysis provides a foundation for understanding castor bean miRNA organ-specific expression patterns. The present study offers an expanded picture of miRNAs for castor bean and other members in the family Euphorbiaceae.

## Introduction

The castor bean (*Ricinus communis* L., Euphorbiaceae, 2 n = 20) is one of most important non-edible oilseed crops and its seed derivatives are often used in aviation oil, lubricants, nylon, dyes, inks, soaps, adhesive and biodiesel. Among all the vegetable oils, seed oil of castor bean is distinctive due to its high level of ricinoleic acid (over 85%), a fatty acid consisting of 18 carbons, a double bond between C9 and C10, and a hydroxyl group attached to C12. In particular, owing to its excellent solubility in ethanol or methanol, seed oil of castor bean was considered as an ideal and unique feedstock for biodiesel production [1]–[3]. Because of its high economic value, castor bean is widely cultivated in tropical, sub-tropical and warm-temperate countries, particularly India, China and Brazil [4]. Due to increased demand for production of castor bean seed oils in many countries, breeding and improvement of varieties are drawing a great attention from breeders [5]. Particularly, genetic improvement of varieties by genetic engineering techniques offers great promises in castor bean [6], [7]. Enhanced efforts should be paid for elucidating the molecular mechanism underlying the regulation of growth and development.

The microRNAs (miRNAs) are endogenous noncoding small RNAs which play significant roles in the regulation of gene expression. Post-transcriptional gene regulation by miRNAs constitutes one of the most conserved and well characterized gene regulatory mechanisms. In higher plants, miRNAs play significant roles in different developmental stages by regulating gene expression at transcriptional and post-transcriptional levels [8]–[12]. Identification and characterization of miRNAs and their targets in diverse species has been a major focus in recent years [13]–[16]. Although a number of miRNAs have been identified from diverse plants, information on identification and characterization of miRNAs in the family Euphorbiaceae, an important resource plant group, is very limited. So far, the miRNA database miRBase[17]–[19] (Release 19, January 2013, http://www.mirbase.org) contains 63 miRNAs identified from castor bean, 28 miRNAs identified from rubber tree (*Heven brasiliensis*) and 10 miRNAs identified from *Manihot esculenta* in Euphorbiaceae. Although 63 miRNAs had been identified from castor bean in the previous study [20], little information on the transcript level and their tissue-specific expression of miRNAs, however, is available in castor bean. Identification and characterization of miRNAs will contribute to the understanding of the molecular basis of regulating developmental and physiological processes in castor bean.

Recently, high-throughput sequencing technologies have been proven to be a powerful strategy to profile miRNA expression pattern and detect novel miRNAs at unprecedented perspectives [21]–[24]. In particular, high-throughput sequencing technologies can be reliably used to measure modest changes in miRNA abundance among different samples; such changes are unlikely to be identified by sequencing low numbers of clones (i.e., traditional small RNA library sequencing) or hybridization-based methods such as small RNA blot and miRNA array analyses. High-throughput sequencing technologies can not only discover novel miRNAs (which produce transcripts in low abundance) due to their ability to generate millions of reads with a determined length, but also characterize their expression among tissues according to their relative abundance. MiRNAs of diverse plants such as maize [25], common bean [26], peanut [27], safflower [28], cucumber [29], soybean [30], cabbage [31], *Panax ginseng* [32] and *Pinus densata* [33], have been investigated using high-throughput sequencing technologies in recent years.

In this study, we performed deep sequencing and bioinformatic analyses of caster bean tissues (leaves, roots, developing seeds and endosperms) to identify and characterize conserved and novel miRNAs, as well as expression patterns of miRNAs among different tissues and at different stages of seed development. We expected that the conserved, novel and differentially expressed miRNAs obtained in this study provide a basis for further investigation of the physiological roles of identified miRNAs and the molecular mechanism underlying the regulation of growth and development in castor bean.

## Results

### Library Construction, Sequencing and Characterization of Small RNA Transcriptomes in Castor Bean

**Figure 1:**
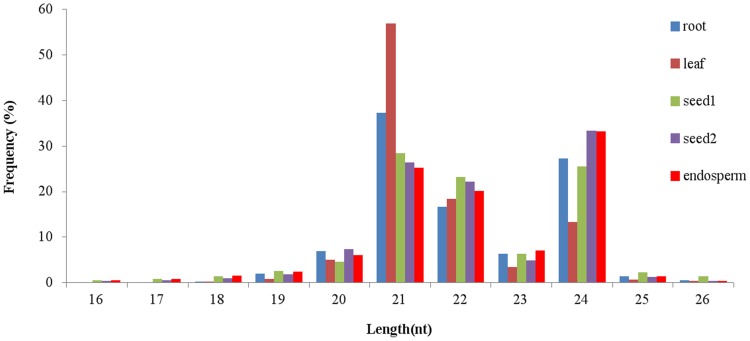
The length size distribution of small RNAs in root, leaf, seed1, seed2 and endosperm libraries in castor bean.

In order to identify and characterize conserved and novel miRNAs in castor bean, we constructed five small RNA libraries from leaves, root tips, developing seeds at the initial stage (seed1) and at the oil fast accumulation stage (seed2) and endosperms, and obtained sequence reads through Solexa high-throughput sequencing technologies. Initially, a total number of 14,259,011 (leaf), 13,467,037 (root tip), 11,423,439 (seed1), 11,334,893 (seed2), 12,955,198 (endosperm) raw reads were obtained. After filtering the low quality reads, adaptor and contaminant sequences, the clean reads were 14,187,024, 13,317,609, 11,098,154, 11,089,507 and 12,553,234 for leaf, root tip, seed1, seed2 and endosperm libraries, respectively. Based on these sequences we analyzed the length distribution and found that among the unique size distribution pattern, most of the reads were distributed between 21 and 24 nt (Figure 1). This observation was consistent with the typical size of miRNA from Dicer digestion products. Among which, sequences with the length of the 21 nt and 24 nt were shown to be significantly in abundance, specifically, the sequences with length of 21 nt was highest abundance in leaf, root tip and seed1 libraries, accounting for 56.82%, 37.22% and 28.42% of the sequence number, respectively, whereas sequences with the length of 24 nt were the highest abundance in seed2 and endosperm, accounting for 33.35% and 33.17% of the sequence number.

Subsequently, we annotated all the reads fall into the length of 16–26 nt from all the five libraries (including leaf, root, seed1, seed2 and endosperm) and obtained 1,742,976, 2,758,394, 2,411,289, 2,944,394, 3,557,270 unique reads (the sequence of a particular type with non-redundancy) for leaf, root, seed1, seed2 and endosperm libraries, respectively. Among them, non-coding small RNAs annotated (snRNAs, snoRNAs, tRNAs, rRNAs and miRNAs) occupy 7,050,077, 5,569,288, 4,714,941, 3,012,704 and 3,742,076 reads in leaf, root, seed1, seed2 and endosperm, respectively (Table 1). In addition, a small proportion of reads could be mapped to coding sequences, which are likely to be RNA degradation products; a small proportion of reads could be mapped to intron sequences, which are likely to be related to the splicing of the host gene to produce pre-miRNA molecules.

**Table 1:** Reads abundance of small RNAs in leaf, root, seed1, seed2 and endosperm libraries.

Category	Reads abundance of total small RNAs				
	Leaf	Root	Seed1	Seed2	Endosperm
Total reads	14259011	13467037	11423439	11334893	12955198
Clean reads	14187024	13317609	11098154	11089507	12553234
Unique reads	1742976	2758394	2411289	2944394	3557270
exon_antisense	117549	108113	62340	88220	98610
exon_sense	484447	269154	271996	281762	265621
intron antisense	212386	328599	233083	272340	317912
intron_sense	878033	768915	608664	691092	764507
rRNA	406058	1463991	1887217	690693	1152370
snRNA	8759	8069	5631	4197	6062
snoRNA	4444	21301	6350	8406	9969
tRNA	437711	398694	410336	208648	290630
miRNA	6193105	3677233	2405407	2100760	2283045
perfect miRNA matching reads	5716106	2938562	863191	564291	814579
miRNA isoform reads	476999	738671	1542216	1536469	1468466
unannotated	5444532	6273540	5207130	6743389	7364508

### Identification of Conserved miRNAs in Castor Bean

To identify conserved miRNAs in castor bean small RNA libraries, the unique reads (excluded reads mapped to snRNAs, snoRNAs, tRNAs and coding sequence or intron sequence) 11,637,637, 9,950,773, 7,612,537, 8,844,149, 9,647,553 from five libraries were subjected to the homolog search against miRBase 19. A number of 6,193,105, 3,677,233, 2,405,407, 2,100,760, and 2,283,045 reads in leaf, root, seed1, seed2 and endosperm libraries respectively, were homologous with known castor bean miRNAs, which accounts for 53.2%, 36.9%, 31.6%, 23.8%, and 23.7% of unique reads from each library, respectively (see Table 1). These observations suggest that known miRNAs are only a small portion and there still may be complicated ingredients in Solexa sequenced data.

In total, 86 conserved miRNAs were detected, covering 26 miRNA families. As shown in Table 2 and Table S1, the most abundant are miR169 (12 members), miR170/171 (nine members), and miR156/157 (eight members). Of the 86 miRNAs, 69 miRNAs were expressed in all five libraries, which accounted for 80.2%; 13 miRNAs (including one miR159/319, nine miR169s, one miR172, two miR399s) were not detected in the leaf library; seven miRNAs (including one miR160, four miR169s, one miR398 and miR2111) were not detected in the root library; ten miRNAs (including nine miR169s and one miR170/171) were not detected in the seed1 library; six miR169s were not detected in the seed2 library; and four miRNAs (including two miR169s, one miR170/171 and one miR399) were not detected in the endosperm tissue.

**Table 2:** Conserved miRNAs and their expression levels among different tissues.

miRNAfamily	ReferencemiRNA	Sequence (5′–3′)	Length (nt)	Reads				
				leaf	root	seed1	seed2	endosperm
156	rco-miR156a	TGACAGAAGAGAGTGAGCAC	20	115967	554938	20415	397950	259577
	rco-miR156b	TGACAGAAGAGAGTGAGCAC	20	116382	555423	20532	398971	261537
	rco-miR156c	TGACAGAAGAGAGTGAGCAC	20	115967	554938	20414	397950	259577
	rco-miR156d	TGACAGAAGAGAGTGAGCAC	20	117010	555518	20426	398098	259709
	rco-miR156e	TTGACAGAAGAGAGAGAGCAC	21	1734	2320	819809	928064	912186
	rco-miR156f	TTGACAGAAGATAGAGAGCAC	21	4581548	2141241	170320	254303	517291
	rco-miR156g	TTGACAGAAGATAGAGAGCAC	21	4574207	2143679	169362	253236	517521
	rco-miR156h	TTGACAGAAGATAGAGAGCAC	21	4581205	2140983	170329	254302	517216
159	rco-miR159	TTTGGATTGAAGGGAGCTCTA	21	11246	8102	8950	4359	1711
160	rco-miR160a	TGCCTGGCTCCCTGTATGCCA	21	100	38	9	28	71
	rco-miR160b	TGCCTGGCTCCCTGTATGCCA	21	93	80	8	28	71
	rco-miR160c	TGCCTGGCTCCCTGAATGCCA	21	63	0	4	22	37
162	rco-miR162	TCGATAAACCTCTGCATCCAG	21	1125	925	1682	866	713
164	rco-miR164a	TGGAGAAGCAGGGCACGTGCA	21	1792	5372	30683	448	2143
	rco-miR164b	TGGAGAAGCAGGGCACGTGCA	21	1788	5256	30613	447	2141
	rco-miR164c	TGGAGAAGCAGGGCACGTGCA	21	1812	5269	30783	447	2150
	rco-miR164d	TGGAGAAGCAGGGCACATGCT	21	2	51	147	3	5
166	rco-miR166a	TCGGACCAGGCTTCATTCCCC	21	1041527	630125	521438	192914	200808
	rco-miR166b	TCGGACCAGGCTTCATTCCCC	21	1040582	629444	521029	192753	200683
	rco-miR166c	TCGGACCAGGCTTCATTCCCC	21	1044631	634097	533841	193804	201735
	rco-miR166d	TCGGACCAGGCTTCATTCCCC	21	1077411	712533	540960	199357	206911
	rco-miR166e	TCGGACCAGGCTTCATTCCCC	21	1040582	629429	521021	192738	200671
167	rco-miR167a	TGAAGCTGCCAGCATGATCTA	21	25258	17649	72490	21432	32506
	rco-miR167b	TGAAGCTGCCAGCATGATCTAA	22	199726	26315	657226	165097	258009
	rco-miR167c	TGAAGCTGCCAGCATGATCTG	21	15711	2542	10441	6967	7664
	rco-miR167d*	TAAAGCTGCCAGCATGATCTA	21	808	456	604	292	148
168	rco-miR168	TCGCTTGGTGCAGGTCGGGAA	21	64409	112339	19109	78883	52530
169	rco-miR169a	CAGCCAAGGATGACTTGCCGG	21	24	417	8	79	456
	rco-miR169b	CAGCCAAGGATGACTTGCCGG	21	24	406	7	64	414
	rco-miR169c	TGAGCCAAGGATGACTTGCCG	21	22	200	6	3	31
	rco-miR169d	CAGCCAAGGATGACTTGCCGA	21	0	152	0	0	5
	rco-miR169e	CAGCCAAGGATGACTTGCCGA	21	0	152	0	0	0
	rco-miR169f	CAGCCAAGGATGACTTGCCGA	21	0	154	0	1	0
	rco-miR169g	TAGCCAAGGATGACTTGCCTG	21	0	0	0	0	9
	rco-miR169h	TAGCCAAGGATGACTTGCCTG	21	0	0	0	0	8
	rco-miR169i	TAGCCAAGGATGACTTGCCCA	21	0	0	0	0	5
	rco-miR169j	TAGCCAAGGATGACTTGCCCG	21	0	27	0	10	30
	rco-miR169k	TAGCCAAGGATGACTTGCCCG	21	0	19	0	8	20
	rco-miR169l	TAGCCAAGGATGACTTGCCCA	21	0	0	0	0	10
171	rco-miR171a	TGATTGAGCCGTGCCAATATC	21	539	260	195	364	1274
	rco-miR171b	TGATTGAGCCGTGCCAATATC	21	539	260	195	364	1274
	rco-miR171c	TGATTGAGCCGTGCCAATATC	21	511	268	193	374	1294
	rco-miR171d	TGATTGAGCCGTGCCAATATC	21	510	257	191	364	1261
	rco-miR171e	TGATTGAGCCGTGCCAATATC	21	516	435	241	432	1487
	rco-miR171f	TGATTGAGCCGTGCCAATATC	21	510	257	191	364	1261
	rco-miR171g	TTGAGCCGCGCCAATATCACT	21	4	107	6	5	0
	rco-miR171h	TTGAGCCGCGTCAATATCTCC	21	27	143	0	24	60
	rco-miR171i	CGAGCCGAATCAATATCACTC	21	2448	78	45	626	635
172	rco-miR172a	GGAATCTTGATGATGCTGCAG	21	0	1	702	24	82
	rco-miR172b	AGAATCTTGATGATGCTGCAT	21	16659	964	1160	2865	3119
	rco-miR172c	AGAATCTTGATGATGCTGCAT	21	16659	964	1160	2865	3119
	rco-miR172d	AGAATCTTGATGATGCTGCAT	21	16659	964	1160	2865	3119
319	rco-miR319a	TTGGACTGAAGGGAGCTCCC	20	21	10	7	9	2
	rco-miR319b	TTGGACTGAAGGGAGCTCCC	20	21	9	7	9	2
	rco-miR319c	TTGGACTGAAGGGAGCTCCC	20	22	21	8	9	2
	rco-miR319d	TTGGACTGAAGGGAGCTCCTT	21	0	10	1	4	1
390	rco-miR390a	AAGCTCAGGAGGGATAGCGCC	21	308	6671	6698	246	554
	rco-miR390b	AAGCTCAGGAGGGATAGCGCC	21	326	6759	6844	254	562
393	rco-miR393a	TCCAAAGGGATCGCATTGATCT	22	31	8	76	101	135
	rco-miR393b	TCCAAAGGGATCGCATTGATCC	22	24	7	35	8	21
394	rco-miR394a*	TTGGCATTCTGTCCACCTCC	20	9	0	10	25	108
	rco-miR394b*	TTGGCATTCTGTCCACCTCC	20	14	0	13	25	110
395	rco-miR395a	CTGAAGTGTTTGGGGGAACTC	21	341	58	10	14	30
	rco-miR395b	CTGAAGTGTTTGGGGGAACTC	21	341	58	10	14	30
	rco-miR395c	CTGAAGTGTTTGGGGGAACTC	21	337	58	10	13	30
	rco-miR395d	CTGAAGTGTTTGGGGGAACTC	21	336	58	10	13	30
	rco-miR395e	CTGAAGTGTTTGGGGGAACTC	21	341	58	10	14	30
396	rco-miR396a	TTCCACAGCTTTCTTGAACTT	21	171	36	951	94	96
	rco-miR396b	TTCCACAGCTTTCTTGAACTG	21	1859	1001	1088	579	1032
	rco-miR396c	TTCCACAGCTTTCTTGAACTG	21	1854	1005	1092	580	1035
397	rco-miR397	TCATTGAGTGCAGCGTTGATG	21	368	3350	509	582	237
398	rco-miR398a	TTCTCAGGTCACCCCTTTGGG	21	1	0	2	1	3
	rco-miR398b	TGTGTTCTCAGGTCGCCCCTG	21	47	60	13	1	3
399	rco-miR399a	TGCCAAAGGAGAGTTGCCCTG	21	177	16	34	70	95
	rco-miR399b	TGCCAAAGGAGATTTGCCCGG	21	1	284	2	7	15
	rco-miR399c	TGCCAAAGGAGATTTGCCCGG	21	1	280	2	7	15
	rco-miR399d	TGCCAAAGGAGAGCTGCCCTG	21	0	1	1	1	0
	rco-miR399e	TGCCAAAGGAGATTTGCC	18	0	5	1	4	7
403	rco-miR403a	TTAGATTCACGCACAAACTCG	21	761	309	368	2293	1061
	rco-miR403b	TTAGATTCACGCACAAACTCG	21	761	309	368	2293	1061
408	rco-miR408	CTGCACTGCCTCTTCCCTGGC	21	77	250	243	94	69
482	rco-miR482*	GGAATGGGCGGTTTGGGAAAG	21	3467	178492	5481	2955	34179
535	rco-miR535	TGACAACGAGAGAGAGCACGC	21	44477	37580	56970	27692	21852
827	rco-miR827*	TTAGATGACCATCAACAAACA	21	2338	132	86	15	201
2111	rco-miR2111*	TAATCTGCATCCTGAGGTTTA	21	180	0	132	109	153
4414	rco-miR4414*	TATGAATGATGCGGGAGATAA	21	3033	22022	60	1905	51

Note: *: New conserved miRNA in known miRBase in other species. The loci on genome were identified for six miRNAs newly identified in this study. rco-miR167d, 29883∶144402:144497:+; homologue: *Arabidopsis thaliana* miR167a; rco-miR394a,b, 30170∶3866594:3866721:+; 30116∶128336:128443:+; homologue: *Arabidopsis thaliana* miR394a,b; rco-miR482, 29586∶144986:145094:-; homologue: *Malus domestica* miR482a; rco-miR827, 28266∶68399:68502:+; homologue: *Gossypium hirsutum* miR827a; rco-miR2111, 29973∶58727:58830:+; homologue: *Arabidopsis thaliana* miR2111a; rco-miR4414, 29729∶702439:702549:+; homologue: *Medicago truncatula* miR4414b.

Compared with the known 63 miRNAs from castor bean in the miRNA database, 23 conserved miRNAs (see Table S1) were newly identified including one miR167 member (rco-miR167d), nine miR169 members (rco-miR169d-i), two miR170/171 members (rco-miR171 h,i), three miR172 members (rco-miR172b-d), one miR393 (rco-miR393b), one miR394 member (rco-miR394b), two miR396 members (rco-miR396b.c), one miR482 (rco-miR482), one miR827 member (rco-miR827), one miR2111 member (rco-miR2111) and one miR4414 member (rco-miR4414). The second structures of 23 new conserved miRNAs were predicted and results were shown in Figure S1. Further, we compared with the miRNAs predicted by Zeng et al. [20] based on genome sequences of castor bean and found that six (including rco-miR167d, rco-miR394, rco-miR482, rco-miR827, rco-miR2111, rco-miR4414) of the 23 miRNAs newly identified in our analyses were reported for the first time in castor bean (see Table 2 and Table S1). Seventy-eight of 83 miRNAs predicted in previous study were confirmed. Five (including one miR169 and four miR399) of 83 miRNAs predicted were not identified in our analysis, probably because the expression of the five miRNAs is related to environmental stress.

The sequencing frequencies for miRNAs in the library can be used as an index for estimating the relative abundance of miRNAs. High-throughput sequencing produced a large number of miRNA sequences, allowing us to determine the relative abundance of miRNAs in castor bean; the frequencies of miRNA families varied largely in different libraries, e.g. most members of miRNA156, miRNA167, miRNA168, miRNA535 were abundant in all libraries, whereas members of miRNA160, miRNA169, miRNA319, miRNA393, miRNA395, miRNA398 and miRNA399 were scarce in all libraries (see Table 2), indicating that expression level of miRNAs varies significantly among different miRNA families in castor bean. In addition, most of the miRNA members displayed a tissue- or developmental stage-specific expression, e.g. miR156e has a low expression in leaf and root libraries and a high expression in the seed libraries; the miR156f, miR156g and miR156 h have the highest expression in the leaf library and the lowest expression in seed1 library.

**Figure 2:**
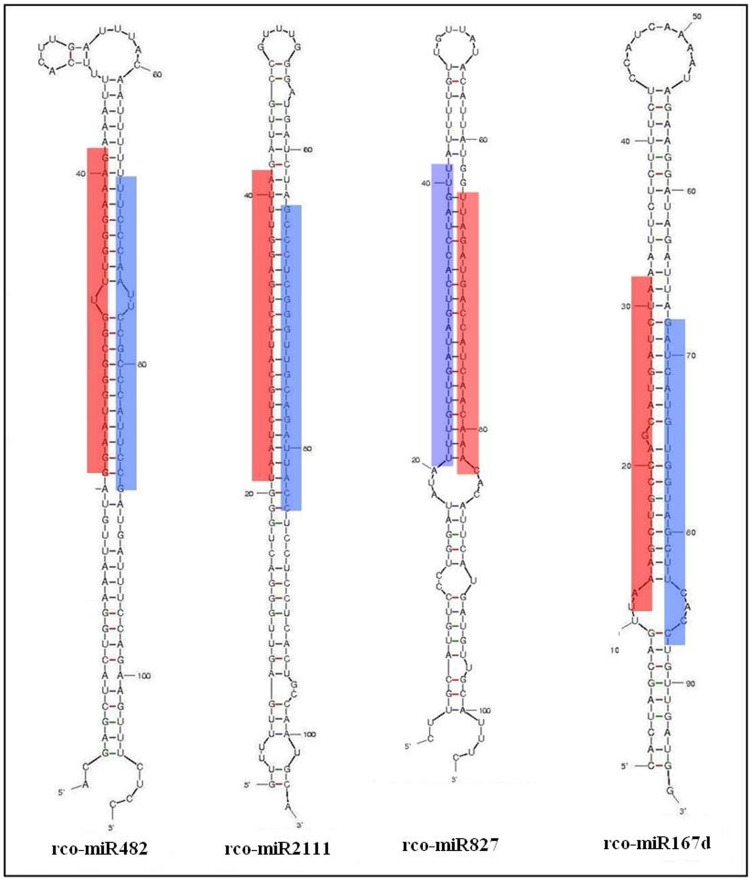
The secondary structures of rco-miR482, rco-miR2111, rcomiR827 and rco-miR167 miRNAs identified from castor bean. Sequences shaded in red and blue, corresponding to miRNA and predicated miRNA*, respectively.

When analyzing the miRNA/miRNA* duplex structure for all conserved miRNAs identified in castor bean, we found that 60 of 86 conserved miRNAs displayed the miRNA/miRNA* duplex structure (Figure 2 for examples), involving 23 families (see Table 3). In contrast, the abundance of miRNA* is significantly lower than their reference miRNAs, except for rco-miR171e* and rco-miR408* (which has abundances higher than their references rco-miR171e and rco-miR408).

**Table 3:** Conserved mature-star miRNAs from castor bean.

miRNAfamily	ReferencemiRNA	Star sequence(5′–3′)	Length (nt)	Reads				
				root	leaf	seed1	seed2	endosperm
156	rco-miR156a	GCTCACCCTCTATCTGTCGCC	21	18	2	15	1	5
	rco-miR156b	GCTCACTTCTCTTTCTGTCAAG	22	18	5	1	5	46
	rco-miR156c	GCTTACTCTCTATCTGTCACC	21	707	9	2	93	156
	rco-miR156d	TGCTCACCTCTCTTTCTGTCAGC	23	1024	1275	2	96	58
	rco-miR156e	TGCTCTCTCCTCTTCTGTCATC	22	0	0	16	21	109
	rco-miR156f	TTTTGTGCTCTTTTTTCTTCTG	22	0	20	0	0	0
	rco-miR156g	GCTCTCTAGTCTTCTGTCATC	21	82	1	0	7	29
	rco-miR156h	GCTCTCTATGCTTCTGTCATC	21	48	106	2	8	82
160	rco-miR160b	GCGTGCGAGGAGCCAAGCATA	21	49	4	0	2	0
	rco-miR160c	ATGAGGGGAGTCATGCAGGCC	21	0	1	0	0	1
162	rco-miR162	TGGAGGCAGCGGTTCATCGATC	22	98	43	23	32	20
164	rco-miR164a	CACGTGCTCCACTTCTCCAAC	25	7	0	0	0	1
	rco-miR164c	CATGTGCCCGTCTTCCCCATC	21	18	12	60	5	8
166	rco-miR166b	GGAATGTTGTCTGGCTCGAGG	21	7533	1685	1581	2078	1428
	rco-miR166c	TGAATGTTGTCTGGTTCGATG	21	131	46	174	9	18
	rco-miR166d	GGGAATGCTGTCTGGTTCGAG	21	0	6	5	1	4
	rco-miR166e	GGAATGTTGTCTGGCTCGAGG	21	7533	1685	1581	2078	1428
167	rco-miR167a	GGTCATGCTCTGACAGCCTCACT	23	91	0	0	2	4
	rco-miR167b	AGATCATGTGGCAGTTTCACC	21	75	94	22	57	79
	rco-miR167c	AGATCATGTGGCAGTTTCACC	21	75	94	22	57	79
	rco-miR167d	GATCATGTGGTAGCTTCACC	20	23	9	1	11	1
168	rco-miR168	CCCGCCTTGCATCAACTGAAT	21	1650	555	116	1729	1276
169	rco-miR169a	CGGCAAGCTGTTCTTGGCTAT	21	207	5	43	126	503
	rco-miR169b	GGCAAGTTGTTCTTGGCTACA	21	4	1	0	0	1
	rco-miR169c	GCAAGACATTCTTGGCTCTAC	21	59	20	0	0	21
	rco-miR169d	GGCAAGTTGTCCTTGGCTACA	21	0	4	0	0	5
	rco-miR169e	GGCAGGTTGTCCTTGGCTAC	20	0	354	0	0	0
	rco-miR169f	GGCGAGCTGTTCTTGGCTACA	21	0	410	0	13	0
	rco-miR169g	GGCAGTCTCCTTGGCTAAC	19	0	0	0	0	3
	rco-miR169i	GGCAGTCAACTTGGCTAAT	19	0	0	0	0	10
	rco-miR169j	GGCATGTCACCTTGGCTAAT	20	0	2	0	2	2
171	rco-miR171a	ATATTGGTCCGGTTCAATAAG	21	5	45	1	9	1
	rco-miR171b	CGAGATATTGGTGCGGTTCAA	21	12	57	14	12	8
	rco-miR171e	TGTTGGAATGGCTCAATCAAA	21	2488	75	458	355	4104
	rco-miR171g	CGATGTTGGTGAGGTTCAATC	21	21	0	0	1	0
	rco-miR171h	GAAGGTATTGGCGCGTCTCAATC	23	2	11	0	3	7
	rco-miR171i	CGTGATATTGGTCCGACTCATC	22	230	18	45	181	24
172	rco-miR172a	GGAGCATCATCAAGATTCACA	21	0	0	119	20	512
	rco-miR172b	GGAGCATCATCAAGATTCACA	21	42	9	3	1	13
	rco-miR172c	GTAGCATCATCAAGATTCACA	21	16	2	0	2	6
	rco-miR172d	GCGGCATCATCAAGATTCACA	21	1	4	0	32	156
390	rco-miR390a	CGCTATCCATCCTGAGTTTCA	21	94	3	161	6	8
	rco-miR390b	CGCTATCCATCCTGAGTTTCA	21	94	3	161	6	8
393	rco-miR393a	ATCATGCGATCCCTTAGGAAG	21	1	1	1	3	4
	rco-miR393b	ATCATGCTATCCCTTTGGATT	21	7	0	4	2	11
394	rco-miR394a	AGGTGGGCATACTGCCAACT	20	2	0	37	9	13
396	rco-miR396a	TTCAAGAAAGCTGTGGGAGA	20	17	17	257	7	8
	rco-miR396b	TTCAATAAAGCTGTGGGAAG	20	899	684	402	371	407
	rco-miR396c	GTTCAAGAAAACTGTGGAAAA	0	0	0	0	3	0
397	rco-miR397	CACCAGCGCTGCATTCAATCA	20	1	0	0	0	0
398	rco-miR398a	CAGAGGAGTGGCTCCCTGAGAACA	24	0	32	6	3	17
	rco-miR398b	GGAGCGACCTGAGAATCACATG	22	127	22	2	1	2
399	rco-miR399d	GGGCATCTCTCGCTTGGCAGG	21	0	1	0	1	4
403	rco-miR403a	AGTTTGTGTGTGAATCTAATT	21	0	2	0	1	3
	rco-miR403b	TCTCTAGTTTGTGCGTGAATC	21	5	3	0	5	1
408	rco-miR408	AAGACTGGGAACAGGCAGTGC	21	1770	357	544	239	337
482	rco-miR482	TTCCCAATTCCGCCCATTCCGA	22	87	1437	209	31	289
535	rco-miR535	GTGCTCCCTATCGTTGTCAAT	21	930	2218	485	890	1272
827	rco-miR827	TTTGTTGATAGTCACCTAGTT	21	471	42	10	3	42
2111	rco-miR2111	GCCCTCGGGTTGCAGATTACC	21	1	0	1	0	5

### Identification of miRNA Isoforms

MiRNAs were initially thought to have a specific sequence of a defined length. Identification of miRNAs from different species has revealed that there are variations in pre-miRNA processing, which could result in miRNA isoforms with one or two nucleotide variation in length or structure from the same locus [26]. Ehrhardt et al. (2010) demonstrated that one fifth of the annotated *Arabidopsis thaliana* miRNAs (miRBase 14) have a stable miRNA isoform of one or two nucleotides longer [34]. Previous studies have revealed that these miRNA isoforms may have functional divergence due to differential associations with AGO proteins [35]–[36]. To identify miRNA isoforms from our transcriptome data, all miRNA reads (including 6,193,105, 3,677,233, 2,405,407, 2,100,760, 2,283,045 reeds from leaf, root, seed1, seed2 and endosperm, respectively) obtained from previous analyses were aligned against miRBase 19), allowing at most two mismatches or four nucleotides in length difference. The total number of isoform variants found for each library was subjected to a filter that consisted of choosing variants that had a total number of reads 50% greater than the number of total reads of their reference miRNA previously reported, so that low-abundance and probable non-functional variants were discarded.

Compared with the length and sequences of the reference miRNAs identified from castor bean genome based on computational prediction in previous study [20], 16 isoform variants from five libraries were detected totally, involving ten families (miRNAs 156, 167, 171, 319, 393, 395, 396, 398, 399 and 403; see Table 4). In the case of miR156, the isoform variant iso-miR156a-d with the 21A absent was detected from four loci (a, b, c and d); the isoform iso-miR156e with a 5′ single nucleotide U/T extension from one locus (e). For the miR167 family, two isoforms with a 3′ single nucleotide A (iso-miR167b) extension or G (isomiR167c) deletion were detected from two loci (b and c). In the case of miR319, two isoform variants with a 3′ single nucleotide T (iso-miR319a-c) and a 5′ single T extension and a 3′di- nucleotide TT deletion (iso-miR319d) were detected from different loci. In the case of miR395, the isoform variant iso-miR319a-e with a 3′ tri- nucleotide TCT deletion were detected from all miR395 loci identified (a, b, c, d and e). Similarly, in the case of miR399, the isoform variant with a 3′ bi- nucleotide GG deletion (isomiR399b-d) was detected from three loci (b, c and d), and the isoform variant with a 3′ tri- nucleotide CAG deletion (iso-miR399e) was detected from the e locus. In the cases of miR171 and miR398, two isoform variants (iso-miR171a,b and iso-miR171g, and iso-miR398a and iso-miR398b) with a 5′ tri- or tetra- nucleotide addition and a 3′ tri- or tetra- nucleotide deletion were detected from different loci. In the other cases such as miR393, miR396 and miR403, isoform variants were produced due to the 1–3 nucleotide addition or deletion in the 3 strand of miRNAs. These results indicated that the isoform variants mainly occurred in several specific miRNA families such as miR156 (isoforms were detected from five loci), miR395 (isoforms were detected from five loci) and miR399 (isoforms were detected from four loci) in castor bean. The variation in length of isoforms identified involved two types: 1) single or several nucleotides addition or deletion in the 3′ strand only (such as miR167 and iso-miR167, miR395 and iso-miR395, miR399 and iso-miR399); and 2) single or several nucleotides addition or deletion both in the 5′ and 3′ strands simultaneously (such as miR156 and iso-miR156, miR171 and iso-miR171, miR398 and iso-miR398).

**Table 4:** miRNA isoforms identified from castor bean.

miRNA	Sequence (5′–3′)	Length (nt)	Reads				
			leaf	root	seed1	seed2	endosperm
rco-miR156a-d	TGACAGAAGAGAGTGAGCAC**A**	21	18327	12383	169	3649	2407
	TGACAGAAGAGAGTGAGCAC	20	97028	541063	20192	393009	255941
rco-miR156e	TGACAGAAGAGAGAGAGCAC**A**	22	24	14	184	112	586
	**T** TGACAGAAGAGAGAGAGCAC	21	1560	1493	817557	925113	906579
rco-miR167b	TGAAGCTGCCAGCATGATCTA	21	24537	17445	70355	20807	31417
	TGAAGCTGCCAGCATGATCTA**A**	22	174186	8647	582840	143017	224446
rco-miR167c	TGAAGCTGCCAGCATGATCTG**G**	22	1074	678	2656	1352	1578
	TGAAGCTGCCAGCATGATCTG	21	13649	1167	5508	5040	5006
rco-miR171a,b	TTGAGCCGTGCCAATATC**ACG**	21	6	0	0	0	1
	**TGA** TTGAGCCGTGCCAATATC	21	505	249	184	355	1228
rco-miR171g	**AGA** TTGAGCCGCGCCAATATC	21	0	1	0	0	0
	TTGAGCCGCGCCAATATC**ACT**	21	4	87	5	5	0
rco-miR319a-c	TTGGACTGAAGGGAGCTCCC**T**	21	11	3	3	5	1
	TTGGACTGAAGGGAGCTCCC	20	8	2	3	2	1
rco-MIR319d	TTGGACTGAAGGGAGCTCC**TT**	22	0	7	0	1	1
	**A** TTGGACTGAAGGGAGCTCC	20	0	0	1	1	1
rco-miR393	TCCAAAGGGATCGCATTGATC	21	1	0	13	21	19
	TCCAAAGGGATCGCATTGATC**T**	22	21	5	27	42	49
rco-MIR395a-e	CTGAAGTGTTTGGGGGAA**CTC**	21	296	47	8	5	16
	CTGAAGTGTTTGGGGGAA	18	12	2	2	7	6
rco-miR396	TTCCACAGCTTTCTTGAA**CTT**	21	99	17	161	3	15
	TTCCACAGCTTTCTTGAA	18	15	7	547	71	60
rco-miR398a	**TGTG** TTCTCAGGTCACCCCTT	21	0	0	1	0	2
	TTCTCAGGTCACCCCTT**TGGG**	21	1	0	1	18	1
rco-miR398b	TGTGTTCTCAGGTCGCCC**CTG**	21	37	56	10	1	1
	**TCA** TGTGTTCTCAGGTCGCCC	21	6	2	1	0	2
rco-miR399b-d	TGCCAAAGGAGATTTGCCC**GG**	21	1	275	1	3	9
	TGCCAAAGGAGATTTGCCC	19	0	1	1	3	5
rco-miR399e	TGCCAAAGGAGATTTGCC**CAG**	21	0	1	0	0	1
	TGCCAAAGGAGATTTGCC	18	0	3	1	1	5
rco-miR403a,b	TTAGATTCACGCACAAACT**CG**	21	688	278	137	358	267
	TTAGATTCACGCACAAACT	19	43	8	184	1601	639

**Figure 3:**
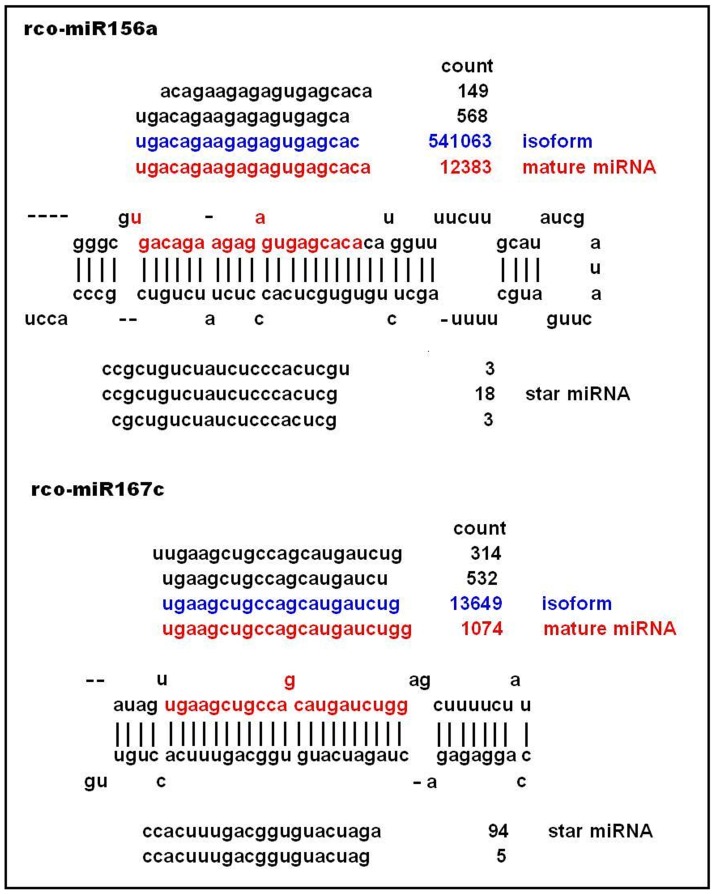
Differential processing of castor bean pre-miRNAs. Stem-loop precursors of rco-miR156a and rco-miR167c pre-miRNAs were aligned against mature (red) and isoform (blue) miRNA sequences. Count data number represents the total number of reads found in leaf libraries.

When inspecting the expression of these isoform variants among five libraries, we unexpectedly found that the expression of these isoforms among different libraries had significant divergence, e.g., in the cases of miR156a-d, miR156e, miR167c and miR171a,b, the variants iso-miR156a-d, iso-miR156e, iso-miR167c and iso-miR171a,b were more highly expressed in all libraries than the rco-miR156a-d, rco-miR156e, rco-miR167c and rco-miR171a,b (Figure 3 for examples); in the case of miR395, rco-miR395a-e had relatively higher expression in the leaf library than its expression in other libraries, whereas the iso-miR395a-e was weakly expressed in all libraries; in the case of miR399, rco-miR399b-d was relatively higherly expressed in root library than other tissues, whereas the rco-miR399b-d was weakly expressed in all libraries; in the case of miR403, rco-miR403a,b was relatively higherly expressed in the leaf library than other libraries, whereas the iso-miR403a,b was higherly expressed in the seed2 library than others; in the case of miR171, rco-miR171g was only present in the root library, whereas the iso-miR171g was present in all libraries except for the endosperm library (see Table 4).

### Expression Patterns of miRNAs among Tissues

Preferential expression of a miRNA in specific tissues might provide clues about its physiological function. To investigate the expression patterns of miRNAs among leaf, root, developing seeds and endosperm in castor bean, read count of each identified miRNA was normalized to the total number of miRNA read count in each library. Based on the relative abundance, we found that the expression of certain members within the miRNA families varied greatly in the given tissues, suggesting functional divergence within the family in castor bean. For example, abundance of the miR156 family varied from 122 reads (rco-miR156e) to 322,939 reads (rco-miR156e) in the leaf library, similar to the case for miR167 family varied from 941 reads to 59,219 reads in the seed1 library (see Table S2). These results indicate that miRNA members in one given miRNA family display clearly different expression levels, probably implying their functional divergence.

**Figure 4:**
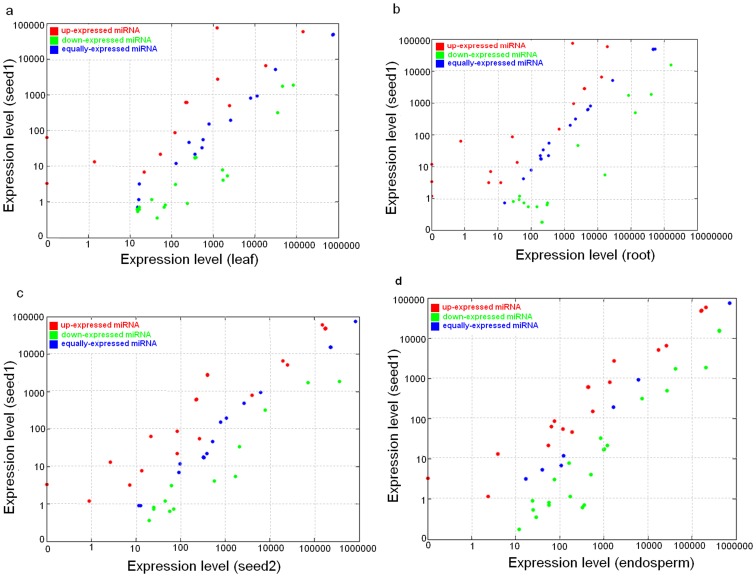
Comparison of expression patterns of miRNAs identified between seed1/root (a), seed1/leaf (b), seed1/seed2 (c), and seed1/endosperm (d).

We compared the expressional differentiation of conserved miRNAs identified between the leaf and seed1, root and seed1, seed2 and seed1, and endosperm and seed1, respectively. We found that 49 out of 69 miRNAs detected between the leaf and seed1 were significantly differentially expressed (log2ratio fold-change >1.0 and P value <0.001, see Figure 4a and Table S2) with 15 miRNAs up-regulated and 34 miRNAs down-regulated in leaf. Similarly, 42 out of 69 miRNAs between the seed1 and root were significantly differentially expressed with 17 miRNAs up-regulated and 25 miRNAs down-regulated in root (see Figure 4b and Table S2). When comparing the expressional differentiation of miRNAs between the seed2 and seed1, endosperm and seed1, respectively, we found that 42 out of 65 miRNAs detected between the seed1 and seed2, and 60 out of 68 miRNAs detected between the endosperm and seed1, were significantly differentially expressed (log2ratio fold-change >1.0 and P value <0.001, see Figure 4c-d and Table S2) with 23 miRNAs up-regulated and 19 miRNAs down-regulated in the seed2, and 23 miRNAs up-regulated and 37 miRNAs down-regulated in the endosperm. It is worthy to note that some families such as miR166 and miR165 were of abundance cross the five libraries, whereas many families such as miR160, miR169, miR171, miR395 were lowly expressed in five libraries. Based on their abundance in the libraries, most members of miR156 family were of higher abundance in vegetable tissues (leaf and root), whereas the rco-miR156e had higher expression in developing seeds than in the leaf and root; the members of miR167 and miR164 had obviously preferential expression among tissues (see Table 2 and Table S2).

### Novel miRNA Detection

One of the most important features for high-throughput sequencing is that it can be employed to detect novel miRNAs in small RNA transcriptome [22], [37]. In the previous study, 83 miRNAs were predicted based on genome sequences in castor bean and 63 of 83 miRNAs predicted were validated and released in the miRNA database [20]. In this study, remaining unannotated reads (5,444,532, 6,273,540, 5,207,130, 6,743,389 and 7,364,508 from leaf, root, seed1, seeds and endosperm, respectively) were mapped to reference genome of castor bean for identifying the genomic location and retrieving the adjoining sequence to help with secondary structure prediction of a miRNA precursor using the MIREAP pipeline (developed by BGI). The resulting reads, with a characteristic hairpin structure, a maximum free energy of ∼25kcal/mol, minimal matched base pairs of miRNA and miRNA* exceeding 16 nt and the sequence length of 20–23 nt, and reads abundance more than 100 at least in one independent library were considered as novel miRNA candidates. As a result, 72 potential miRNA candidates were identified with typical stem-loop structure (Figure S1), the negative folding free energies ranged from 25.4 to 103 (kcal/mol), and diverse loci in castor bean genome (see Table 5 and Table S3). Of the 72 potential miRNAs, 24 represented both the miRNA and miRNA* and 48 were miRNA*-deficient cases (having only the 5′ arm or 3′ arm sequences) (see Table 5 and Table S3). Fifty-three of these novel miRNA candidates were expressed in at least two independent libraries, and 19 of these candidates were expressed in a single library. A recently published article proposed precise and strict new miRNA annotation criteria by Meyers et al. [38]. Besides the primary criteria used by Mireap, two elementary requirements are demanded in high-throughput sequencing data analysis: (i) high-throughput sequencing data should represent both the miRNA and miRNA*; and (ii) in miRNA*-deficient cases, isolation and sequencing of the candidate miRNA should come from multiple and independent libraries. Based on these precise criteria, 58 of 72 novel miRNA candidates were categorized as highly confident. Fourteen miRNA candidates identified by Mireap did not meet Meyers et al.’s criteria (see Table 5).

**Table 5:** Novel miRNAs identified from castor bean.

miRNA	Sequences (5′–3′)	Length(nt)	Reads					RNA*	No of loci
			leaf	root	seed1	seed2	endosperm		
Rco-miR001^a^	TTGGAGGATAGTTTCAGGCCGG	22	0	127	0	19	0	no	1
Rco-miR002^a^	GTGGACGTGCCGGAGTGGTTA	21	1565	3188	0	1221	1656	no	2
Rco-miR003^b^	TCTGATAGCAAAAGATAGAAC	21	814	0	0	0	0	no	1
Rco-miR004^a^	CAACGGATAGGTATACAGTTTT	22	302	0	412	100	0	no	1
Rco-miR005^a^	TCTGAAATTGCAGAGCCTAAA	21	225	372	124	205	343	no	1
Rco-miR006^a^	TCTTTGTAGTTTTGATCCGGAG	22	1312	2054	1735	1394	1314	no	1
Rco-miR007^a^	AGAGAAGGATGGTAGAGATGGTT	23	10	0	27	0	276	no	2
Rco-miR008^a^	TATCTTTGTAGTTTTGATCCGG	22	322	705	547	0	0	no	1
Rco-miR009^a^	TGAAGATGAAGAGCTATGTTTGA	23	867	14	10	131	0	no	1
Rco-miR010^a^	TGAGGAAGAGGATGACTTTGGA	22	0	110	0	59	22	no	1
Rco-miR011^a^	TCTCTAATTCGCTTGGTGCAG	21	193	178	43	71	58	yes	1
Rco-miR012^a^	CAATTGGATCGTTATTTGCTA	21	113	137	87	167	132	no	1
Rco-miR013^a^	AGGTGCAGGTGTGAGTGCAGG	21	17	123	96	0	18	yes	1
Rco-miR014^a^	TAATCTTGCTAACGGACTAAA	21	29	163	0	0	55	yes	1
Rco-miR015^a^	GCCGCTATGGTGAAATCGGT	20	407	0	0	0	0	yes	1
Rco-miR016^a^	AAGCCTGCGAGAGAGAGTTGG	21	0	0	0	371	346	yes	1
Rco-miR017^a^	AGGCCGATGACGATTAGAGGACG	23	0	147	0	0	0	yes	2
Rco-miR018^b^	TTCAAAAGGAGAACAAGGATAA	22	457	0	0	0	0	no	1
Rco-miR019^a^	ACATCCTTGAAGCTAACTCTA	21	45	19	465	386	573	yes	1
Rco-miR020^a^	AGGCAGTCATCTCTTGGCTAC	21	0	0	0	0	163	yes	1
Rco-miR021^a^	CGAGTCATCTGACAGAAGTAG	21	0	443	0	0	0	yes	1
Rco-miR022^b^	AGTGGGCGGAAAGGGGGGGTA	21	189	0	0	0	0	no	1
Rco-miR023^a^	TTTTATCACCGTCAGATTCTA	21	127	333	77	221	185	no	1
Rco-miR024^a^	TTTTGCCTACACCACCCATTCC	22	0	637	621	0	0	no	4
Rco-miR025^a^	AATAGTGATTGTGATATTGGCC	22	323	0	10	10	0	yes	1
Rco-miR026^a^	ATTTTAGGAAGGGAATGAACA	21	249	768	368	653	431	yes	1
Rco-miR027^b^	TTATTTTGATTTTGGACGTTTC	22	180	0	0	0	0	no	5
Rco-miR028^a^	TCTTATAGCAATCAGGGGACTTG	23	0	16651	0	0	0	yes	1
Rco-miR029^a^	TATGGGGGGATCGGGCAATAT	21	3079	8498	6191	4222	2431	yes	1
Rco-miR030^a^	GTCTGGGTGGTGTAGTCGGTT	21	3842	3735	5213	5004	5369	no	1
Rco-miR031^a^	TGTCGCTGGAGAGATGGCGCCA	22	132	114	64	0	0	no	1
Rco-miR032^a^	GAGGTCCTGTAGGGAGAGTGG	21	14	33	11443	0	29	yes	1
Rco-miR033^a^	TCCGGAGAGATTTGTGGACGA	21	237	0	418	0	285	no	1
Rco-miR034^a^	TCAGGTGGAGAATCAAACAGA	21	171	0	167	600	419	no	1
Rco-miR035^a^	TCCGGAGAGATTTGTGGACGAT	22	0	0	418	0	285	no	1
Rco-miR036^a^	CATGGACCAGAAGGCATATAC	21	103	82	0	84	66	no	1
Rco-miR037^a^	CTGAGACTTGAGGGATAGGTGTT	23	0	579	111	0	0	no	5
Rco-miR038^a^	TGACGTGGCATGAACTTCGGCA	22	923	641	376	1013	1707	no	1
Rco-miR039^a^	TAGAGCCAAGAATGACTTGCCGG	23	0	0	0	204	411	yes	1
Rco-miR040^a^	ACTCTCTCTGAAGGCTTCAAA	21	3199	1179	935	4583	4525	no	1
Rco-miR041^a^	TCCGGAGAGATTTGTGGACGAT	22	418	0	515	0	285	yes	1
Rco-miR042^a^	TCTGTCGCAGGAAAGATGGTAC	22	0	3225	76	0	863	yes	1
Rco-miR043^a^	TTTGCATGACCTGGGAGACGT	21	81	9617	243	28454	25073	no	1
Rco-miR044^a^	TGGAAATTTCTGGGTTGGAGG	21	0	2789	2941	896	314	no	1
Rco-miR045^b^	ATCAAATAAGGAAGAATCGAG	21	0	0	0	121	0	no	1
Rco-miR046^b^	TCGAAAGAGATATCAAGGACTG	22	0	0	0	1789	0	no	1
Rco-miR047^a^	GGAGGCCTTTGAGCAGAGTGGA	22	0	0	118	40	0	yes	1
Rco-miR048^b^	TTGGCATCAGAGGAGTCAAGC	21	105	0	0	0	0	no	1
Rco-miR049^a^	TAGGCAAAGCATCAGGATTCAT	22	2121	434	0	0	0	no	2
Rco-miR050^a^	TGTTTTTTGATCAGGACCATAA	22	174	167	201	68	139	no	1
Rco-miR051^a^	CTGTCGCAGGAGCGGTGGCACC	22	687	523	23	0	0	yes	1
Rco-miR052^a^	GGTATTGGACGGGTTGGCAAGA	22	9127	19777	4389	8140	1429	yes	1
Rco-miR053^a^	TCGAACCCAACTAGAAGATCTC	22	0	0	1225	2281	1379	no	4
Rco-miR054^a^	TATGGGAGGCATGGTCAGAAA	21	290	5820	886	867	417	no	1
Rco-miR055^a^	TGGACAAGTAGAGGTTACTAAT	22	0	214	244	422	472	no	1
Rco-miR056^b^	TCTGGATGAAGGCTGGAGTGAT	22	0	0	549	0	0	no	1
Rco-miR057^a^	GCCGCTATGGTGAAATCGGT	20	407	17	0	15	0	no	1
Rco-miR058^b^	TGAGGTTGGGTTGGACGACATA	22	0	1470	0	0	0	no	1
Rco-miR059^a^	CAGCAAGGATTAAGGGACATTT	22	296	0	556	0	0	no	1
Rco-miR060^b^	TCTGAAGCTGTGAATGGGAAT	21	0	0	0	277	0	no	2
Rco-miR061^a^	GAACGGCATTTGTAGCCCAGGAG	23	101	35	17	10	0	yes	1
Rco-miR062^a^	TCTGAATCAGGCTCTATATTAG	22	0	53	0	159	0	yes	1
Rco-miR063^b^	TTGAACAGTAGGAAGAGGGTTT	22	0	0	0	328	0	no	1
Rco-miR064^a^	TCTTTATATAGAGGTCTCGGAG	22	2595	1375	1103	1600	1864	no	1
Rco-miR065^a^	TTTTGTGCCAAGAACGTTGTTT	22	237	121	48	0	198	no	5
Rco-miR066^a^	TGGATAAGTTTCAGGAGATCTC	22	667	833	795	822	0	yes	1
Rco-miR067^b^	TGGGCTTTGAAGAAGAAGGTA	21	0	0	110	0	0	no	1
Rco-miR068^a^	TCATCAGATGAAGAGCATGACC	22	1064	0	933	0	0	no	1
Rco-miR069^b^	TGGGCTAGAGCATTAGAAGTTT	22	0	0	129	0	0	no	1
Rco-miR070^a^	TCTGGGAGTAGATTGAAGTGAA	22	1182	0	0	1475	0	no	1
Rco-miR071^b^	ATTGAGTTGGTAGAAGGTGCAA	22	0	0	140	0	0	no	1
Rco-miR072^a^	TTAGGAAAGCAGCTTGACACGTG	23	0	0	36	189	0	yes	1

Note: ^a^: these candidates meet Meyers et al.’s criteria; ^b^: these candidates do not meet Meyers et al.’s criteria.

### Predicted Targets of Castor Bean miRNAs

According to Allen et al. and Schwab et al.’s methods [39], [40], we predicted targets of the 95 miRNA candidates (including 23 new conserved and 72 novel miRNAs) using the currently annotated mRNAs of genes in the castor bean (from the CBGD database http://castorbean.jcvi.org). As a result, 80 of 95 miRNA candidates were identified to have their target genes, involving 482 miRNA:target pairs. The function of these target genes were broadly involved in the growth and development process of castor bean. The predicted target genes of these 95 miRNA candidates and their potential functional annotations are listed in Table S4.

### Validation of the Putative miRNAs Newly Identified in Castor Bean

**Figure 5:**
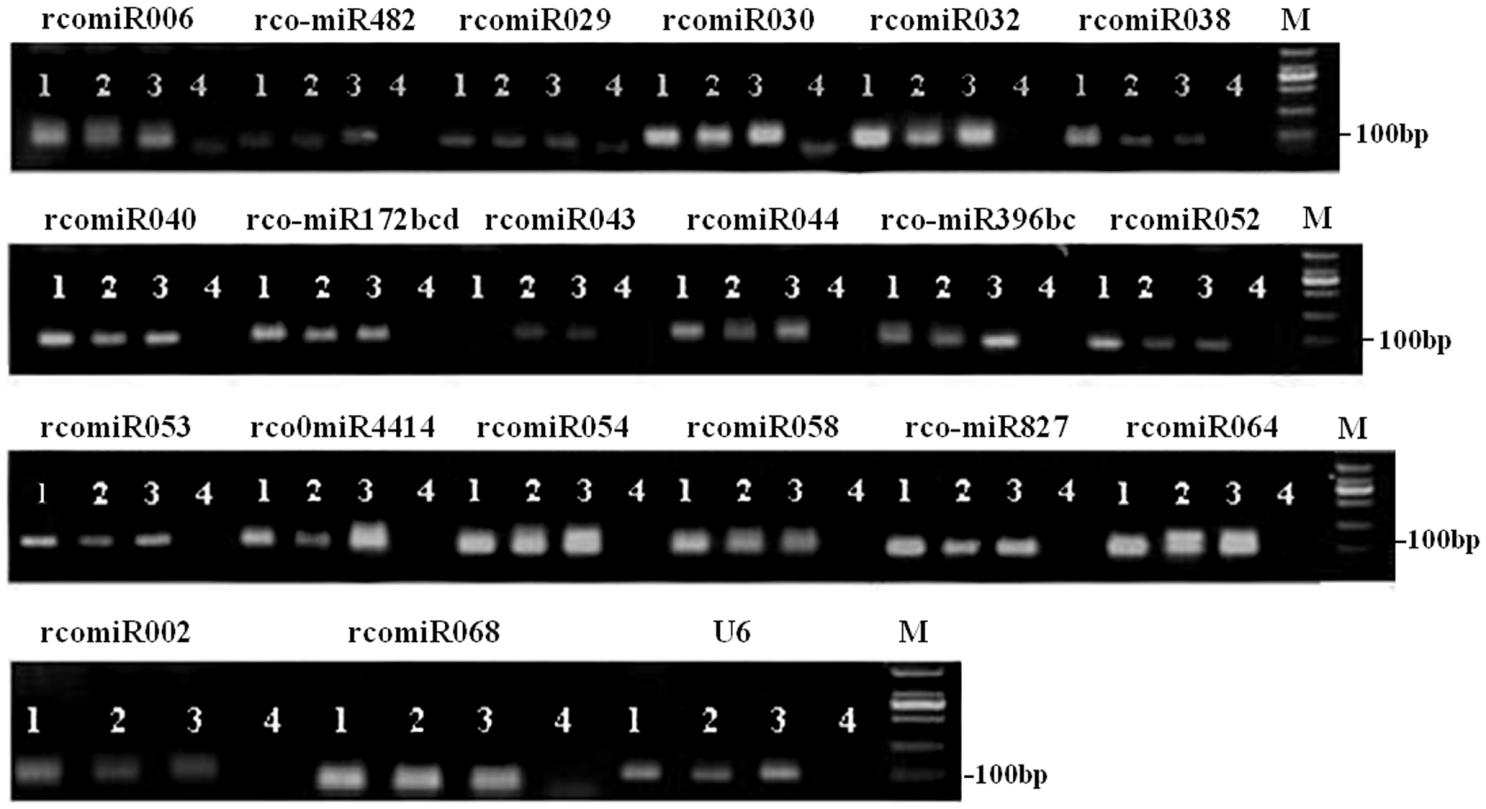
Validation of the 20 miRNAs newly identified using the RT-PCR method. The numbers 1, 2 and 3 showed that the bands amplified using cDNAs as templates obtained from developing seeds, root tips and leaves, respectively. The number 4 showed a negative control (NTC, i.e., no template in PCR reaction). M denoted markers. The amplified bands were separated in 1.5% agarose gel.

To validate the 95 miRNA candidates newly identified by high-throughput sequencing results, RT-PCR analysis was performed according to the method described in “Materials and Methods”. Using first-strand cDNAs obtained respectively from leaves, root tips and developing seeds, 20 primer pairs showed clean amplification bands for miRNAs PCR products including five conserved miRNA families (rco-miR172bc-d, 396b-c, 482, 827 and 4414) and fifteen novel putative miRNAs (Rco-miR002, Rco-miR006, Rco-miR029, Rco-miR030, Rco-miR032, Rco-miR038, Rco-miR040, Rco-miR043, Rco-miR044, Rco-miR052, Rco-miR053, Rco-miR054, Rco-miR058, Rco-miR064 and Rco-miR068, see Figure 5), suggesting the 20 miRNAs newly identified were validated by RT-PCR amplification. When comparing the abundance of these miRNAs validated in five miRNA libraries, we found these miRNAs were relatively more abundant than other miRNAs newly identified (see Table 2 and Table 5). Those miRNAs newly identified with low abundances were not validated by RT-PCR amplification probably because of their low expression levels in these tissues tested. These RT-PCR results exhibited the same expression profiles as the original high-throughput sequencing results.

## Discussion

Although miRNAs have been studied extensively in diverse plant species in these years, limited knowledge is known for plant species in the family Euphorbiaceae. Based on complete genome data of castor bean, the study on a genome-scale computational prediction of miRNAs combined with experimental analysis [20] provided a basis for further characterization and functional analysis of miRNAs in Euphorbiaceae species. The current study using high-throughput sequencing method greatly enriches our knowledge in identifying miRNAs in castor bean and facilitates more particular and specific miRNA studies castor bean and other members of the family Euphorbiaceae as well.

High-throughput sequencing analyses have become one of the major sources supporting miRNA annotations [22]–[24]. This study is the first report on identification and characterization of miRNAs and generates a large number of small RNA sequence reads using high-throughput sequencing techniques in castor bean. Studies to elucidate the number of miRNA molecules sequenced from these small RNA sequence reads are still needed for more accurate small RNA profiling studies. In term of reads, the small RNA libraries sequenced finally yielded a large number of unannotated reads after new miRNA screen in this study. These remaining unannotated reads could remain for further analyzing characterization of siRNA populations in castor bean.

Usually, miRNA isoform variants are considered to be a consequence of inaccuracies in Dicer pre-miRNA processing [41]. However, sequence length variation often have been overlooked, as small variations in the sequence length might not have been thought to alter the function of individual miRNAs, as they are directed to their target genes by base pairing [34]. Recent studies had showed that miRNAs and their isoform variants in length broadly co-existed and these variants might lead to functional differentiation, in particular, when the variation occurs in the 5′-end and gives rise to a alternation of the miRNA and argonaute (AGO) binding [36], [42]. A decrease in abundance of the 21 nt isoform variant reduces miR168 homeostasis and leads to developmental defects in *Arabidopsis* and sequence length heterogeneity for plant miRNAs often is essential for correct plant development and environmental responses [36]. Although most of the isoform variants identified from the length variant group exhibit 3′ heterogeneity, little is known about the biological interest of the variation in length occurring in 3′-end of miRNAs. In this study, small RNA sequences from libraries were considered as miRNA isoforms only if they were similar to a reference miRNA identified in miRBase and had a significantly greater number of reads compared to those found for the reference miRNA in all five libraries. From these analyses for isoform identification, 16 miRNA isoforms involving 10 miRNA families were added to the total number of conserved miRNA families identified in castor bean. Six miRNA isoforms displayed 5′ heterogeneity and ten displayed 3′ heterogeneity. Whether these isoform variants detected in castor bean have functional differentiation and play different regulatory roles in plant growth and environmental responses are yet unknown. The expressional differentiation of these isoform variants and their references among tissues, however, imply their functional divergence, if these isoform variants have their biological interest. In addition, those variant sequences with missing bases and low frequencies produced from high-throughput sequencing could be viewed as degradation products or pyrophosphate sequencing errors.

Application of deep sequencing technology can shed considerable novel lights hidden in the small RNA transcriptome data not only for identification of new conserved miRNAs, but also for successful discovery of novel miRNAs with high accuracy and efficiency [41]. Our current study has led to the discovery of 23 new conserved and 72 novel miRNA candidates in castor bean. These new miRNA candidates largely enriched the miRNA database for castor bean and Euphorbiaceae members. However, only seven new conserved and 15 novel miRNAs were validated using experimental RT-PCR method, though 58 of 72 novel miRNA candidates had been categorized as highly confident according to previous strict miRNA annotation criteria, with 35 represented both the miRNA and miRNA*. Most of novel miRNA candidates identified in this study have not been validated. The most likely reason is due to the limit of RT-PCR method when target miRNAs tested have a low expression [23], [37]. Thus, validity of these novel miRNA candidates need to be further confirmed.

When comparing the numbers of miRNAs identified using the same high-throughput sequencing approach between rubber tree [43] and castor bean, we found that castor bean appeared to have less conserved miRNAs (86) involving 27 miRNA families than rubber tree which had 115 conserved miRNAs, covering 56 families. Further, we found that all homologs of 27 conserved miRNA families of castor bean in rubber tree, but we did not find any homolog of the 72 novel miRNAs identified from castor bean in other members of Euphorbiaceae including rubber tree [43], [44], *Jatropha curcas* [45] and *Manihot esculenta* [46], implying that the 72 novel miRNAs detected might represent castor bean species-specific miRNAs. Compared to the target genes identified in other plants, rco-miR167, rco-miR172 and rco-miR482 exhibited similar targets to their homologs in Arabidopsis [47] and maize [25]. However, four conserved miRNAs newly identified (including rco-miR396, rco-miR827, rco-miR2111 and rco-miR4414) and most of the novel miRNAs in castor bean displayed species-specific targets.

In addition, high-throughput sequencing technologies can serve as a powerful miRNA expression profiling tool to identify the differentially expressed miRNAs, providing the basis for future analysis of miRNA functions and elucidating underlying mechanisms in regulating diverse molecular and physiological pathways [12], [37]. In the study, comparison of their expression patterns among different tissues shows that 49, 42, 42 and 60 of 86 conserved miRNAs are significantly differentially expressed between seed1/leaf, seed1/root, seed1/seed2 and seed1/endosperm, respectively. Similarly, many of the miRNA*, isoform variants and novel miRNAs identified in this study presented differential expression patterns among tissues sampled. Although the biological function of miRNAs in castor bean is unclear the expressional differentiation of these miRNAs among tissues provides a clue for further investigation of the physiological roles of miRNAs in castor bean. Castor bean is of an important oilseed crop worldwide, containing significant amounts of lipid and protein. In this study, we searched for miRNAs that might play a function in regulating biological processes related to the biosynthesis of lipid and protein in developing seeds and endosperms. Our results demonstrated that ten miRNAs (rco-miR156f,e, rco-miR159, rco-miR168, rco-miR390a, rco-miR393a, rco-miR396a, rco-miR408, rco-miR003 and rco-miR020) had 21 target genes, which were involved in amino acid metabolism, fatty acid metabolism and lipid metabolism with differential expressions at different stages of seed development. These results imply that the ten miRNAs might have a physiological role in regulating lipid and protein biosynthesis in castor bean.

In summary, we have identified and characterized a large number of miRNAs from castor bean, analyzed their expression and predicted the putative targets of these miRNAs. It will be very important to experimentally characterize these miRNAs and their downstream targets, as this will lead to a better understanding of the function relationship and mechanism of miRNAs in the regulation network. In particular, our high-throughput sequencing approach to miRNA discovery suggests that a significant number of novel miRNAs remain to be further analyzed and characterized. The current study is the first report on identification and characterization of miRNA using the high-throughput sequencing approach in castor bean.

## Materials and Methods

### Ethics Statement

No specific permits were required for the described field studies. No specific permissions were required for these locations and activities. The location is not privately-owned or protected in any way and the field studies did not involve endangered or protected species.

### Sample Preparation and Total RNA Extraction

Seeds of castor bean var. ZB306 elite inbred line (provided kindly by Zibo Academy of Agricultural Sciences, Shandong, China) were cultivated in the greenhouse of Xishuangbanna tropical botanical garden (Kunming branch) with the temperature of day at 24–26°C and night at 18–20°C with the humidity controlled at 60–80%. Leaf tissue was collected from a fully expanded young leaf and root tips were collected, washed and dissected. Immature seeds at two different stages, i.e. seed1 at the initial stage (15 days after pollination) and seed2 at the fast oil accumulation stage (35 days after pollination) of seed development, were collected. Endosperm tissue was dissected from the immature seeds (40 days after pollination). The developing seeds did not start to accumulate TAG at the initial stage (seed1) and fast accumulated TAG at the fast oil accumulation stage (seed2, see Figure S2). Total RNA was extracted from the leaf, root tip, immature seed (seed1 and seed2) and endosperm tissues separately using Trizol (TaKaRa, Dalian, China) following the manufacturer’s protocol. The quality of total RNA samples was tested using both the NanoDrop Spectrometer (ND-1000 Spectrophotometer, Peqlab) and agarose gel (1.5%) electrophoresis.

### Small RNA Library Construction and Sequencing

Total RNA samples were firstly processed by 15% denaturing polyacrylamide gel electrophoresis (PAGE). The small RNA fragments in the range of 16–30 nt in length were isolated from the gel and purified by sRNAs gel extraction Kit (TaKaRa Bio, Otsu, Japan). Then, the 5′ and 3′ termini of the small RNA were linked with proprietary adapters sequentially and RT-PCR was performed to amplify RNA to DNA, which can be used as templates to produce sequencing libraries. At last, approximately 20 µg sequencing libraries were produced and Illumina Solexa Genome Analyzer was employed to sequence the generated libraries.

### Small RNA Sequencing Analysis

After sequencing, we trimmed the adaptor sequences, filtered out the low quality tags and eliminated contamination of adaptor sequences. Non-coding RNAs including rRNA, tRNA, snRNA and snoRNA were identified by reads alignment to the Pfam 10.1 (http://www.sanger.ac.uk/software/Rfam) and GeneBank databases. After removing non-coding RNAs, the clean small RNA sequences ranging from 16–28 nt were collected and mapped to the castor bean genome for getting the unique reads with abundance and position on the genome using SOAP 2.0 program (http://soap.genomics.org.cn/). The unique RNA sequences that perfectly matched the castor bean genome were subjected to subsequent analysis. Sequence reads overlapping with exons and introns of mRNA were excluded to avoid DNA contamination or mRNA degradation products.

### Identification of Conserved, Isoform and Novel miRNAs

In order to determine conserved miRNAs, the trimmed unique reads were aligned against the mature or precursor of conserved castor bean miRNAs in the miRBase [48]. Only the small RNA sequences that perfectly matched known castor bean miRNAs were considered to be conserved miRNAs. To find new conserved miRNAs, the remaining reads were aligned with mature plant miRNA sequences in miRBase allowing at most two mismatches. According to the genomic positions of new conserved miRNA candidates identified, we retrieved the flanking genomic sequences around matched loci to form possible precursors of candidate miRNAs with the Mfold program [49]. Those candidate sequences containing a typical RNA stem-loop with at least 18 bp in matched regions and having folding energy no greater than −18 kcal/mol were considered as new conserved miRNAs. Meanwhile, we inspected stem-loop structures for each miRNAs identified in castor bean and defined the star miRNA sequences based on Dicer-cleavage rules as implemented in the miRDeep software tool [50].

With the purpose of identifying miRNA isoforms, the sequence reads from all libraries that perfectly mapped in the annotated miRNA precursor sequences but not representing annotated miRNA mature and star sequences, were not shifted more than four positions from their original mature or star 5′ position and have a total number of reads 50% greater than the total reads of their reference miRNA were considered as isoform miRNAs in castor bean. If no reference miRNA for a variant was previously detected in all libraries, the variant with the highest frequency was considered.

To identify the novel miRNAs, the unannotated reads that were identical to genome sequence were collected and the flanking sequences around matched position were retrieved. The MIREAP pipeline (https://sourceforge.net/projects/mireap/) was used to analyze their characteristic hairpin structure of miRNA precursor. Those reads which could meet criteria including having a characteristic hairpin structure and the Dicer cleavage site with a maximum free energy of ∼25kcal/mol, minimal matched base pairs of miRNA and miRNA* exceeding 16 nt, the sequence length of 20–23 nt and the reads abundance >100, were considered as novel miRNAs. The filtered pre-miRNA sequences were folded again using Mfold and checked manually.

### Validation of miRNAs Newly Identified

To validate castor bean miRNAs newly identified in this study, a modified oligo (dT) primers RT-PCR approach as described by Fiedler et al. [51] was performed. Briefly, after total miRNAs were extracted from plant tissues, polyA tails to all transcript miRNAs were added, and then transcript miRNAs with polyA tails were reversely transcribed into cDNAs using a set of 12 modified oligo(dT) primers containing a unique sequence tag at the 5′ end and two bases at the 3′ end. This step reaction converts all miRNAs into cDNAs with ∼90bp length. Further, RT-PCR amplification is achieved using a primer specific to the miRNA in interest and a primer specific to the tag.

In our study, total miRNA was isolated from leaves, root tips and developing seeds of castor bean using Plant MicroRNA Extraction Kit (BIOTEKE, Beijing, China), following the manufacturer’s instructions. MiRNA reverse transcription reactions were performed using One Step miRNA 1st cDNA Synthesis Kit (HaiGene Biotech, Haerbin, China) in a 20 µL reaction solution containing 1000 ng miRNAs, 4 µL 4x One Step miRNA RT solution, 2 µL 10x miRNA RT Primers, and RNase- free water was used to adjust the total volume of the reverse transcription reaction to 20 µL. The miRNA reverse transcription reactions were incubated in an Eppendorf Mastercycler (Eppendorf North America, Westbury, NY) for 60 min at 37°C, followed by 5 min at 95°C, and then 4°C until further use. For PCR amplification, 86 specific primers were designed based on mature miRNA sequences for amplifying 95 miRNAs new indentified (see Table S4). The RT-PCR reactions were performed in a 10 µL volume containing 1 µL diluted reverse transcription product, 1×PCR buffer, 0.2 mM dNTPs, 2.0 U EasyTaq DNA polymerase (TransGen Biotech, Beijing, China), and 0.5 µM specific miRNA primer and universal primer (5′-TTACCTAGCGTATCGTTGAC-3′) on Eppendorf Mastercycler. The PCR reaction conditions used were as follows: 2 min at 95°C, followed by 38 cycles of denaturation for 5 s at 95°C, annealing for 5s at 55–60°C, extension for 35s at 70°C, and then 4°C. PCR amplification products were confirmed on 1.5% agarose gel.

### Differential Expression Analysis

To investigate the differentially expressed miRNAs among castor bean leaf, root, seed1, seed2 and endosperm, miRNAs considered for this analysis were the conserved miRNAs (Table 2). Firstly, each miRNAs read count was normalized against the total number of miRNA reads in each given sample. Subsequently, the fold-change (log2(sample1/sample2) and P-value were calculated from the normalized expression, and significantly difference of a given miRNA was determined by the P≤0.001 and fold-change ≥1 in two samples.

### Prediction of miRNA Targets

The whole genome and transcript databases of castor bean (http://castorbean.jcvi.org/index.php) provide a rich resource for predictions of miRNA targets. The putative target sites of miRNA candidates were identified by aligning the miRNA sequences with the genome and transcript database of castor bean. Allen et al.’s and Schwab et al’s criteria [39], [40] were used in our analysis, i.e.: each G:U wobble pairing was assigned 0.5 point; each indel was assigned 2.0 points; all other noncanonical Watson-Crick pairings were each assigned 1.0 point; no more than two adjacent mismatches in the miRNA/target duplex with a minimum free energy (MFE) of the miRNA/target duplex 75% greater than the MFE of the miRNA bound to it’s perfect complement.

## Supporting Information

Figure S1 The second structures of newly identified 95 miRNAs including 23 conserved (*) miRNAs and 72 novel pre-miRNAs in castor bean.
(DOC)

Figure S2 **Developing seeds of castor bean and lipid (triacylglycerols, TAG) accumulation at two different developmental stages.**
(DOC)

Table S1 **The conserved miRNAs identified from castor bean and their distribution among miRNA families.**
(DOC)

Table S2 **The expressional differentiation of conserved miRNAs identified between seed1/leaf, seed1/root, seed1/seed2, seed1/endosperm, respectively.**
(XLS)

Table S3 **Novel rco-miRNAs identified and their expression levels in castor bean.**
(XLS)

Table S4 Putative targets for the conserved 23 miRNAs newly identified (*) and 72 novel miRNAs in castor bean.
(XLS)

Table S5 **The 86 primers designed for RT-PCR amplification of 95 miRNAs newly identified in this study.**
(XLS)
