# Supplementary material for: Transcriptome-Wide Identification and Characterization of MicroRNAs from Castor Bean (Ricinus communis L.)
Source: PLoS One. 2013 Jul 24;8(7):e69995. doi: 10.1371/journal.pone.0069995 (PMC3722108; doi:10.1371/journal.pone.0069995)

**Figure S1.** The second structures of newly identified 95 miRNAs including 23 conserved (*) miRNAs and 72 novel pre-miRNAs in castor bean.

rco-miR167d*
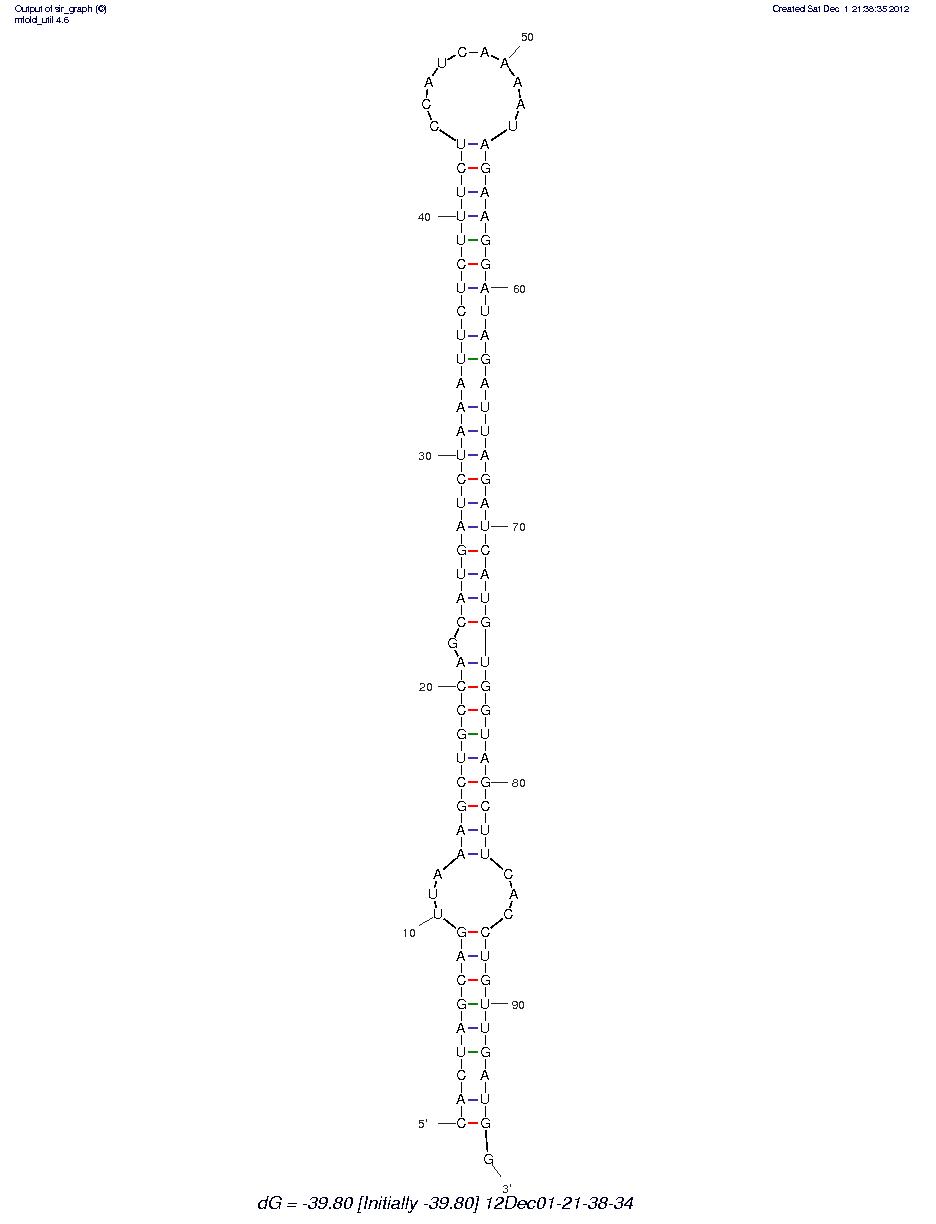


rco-miR169d*


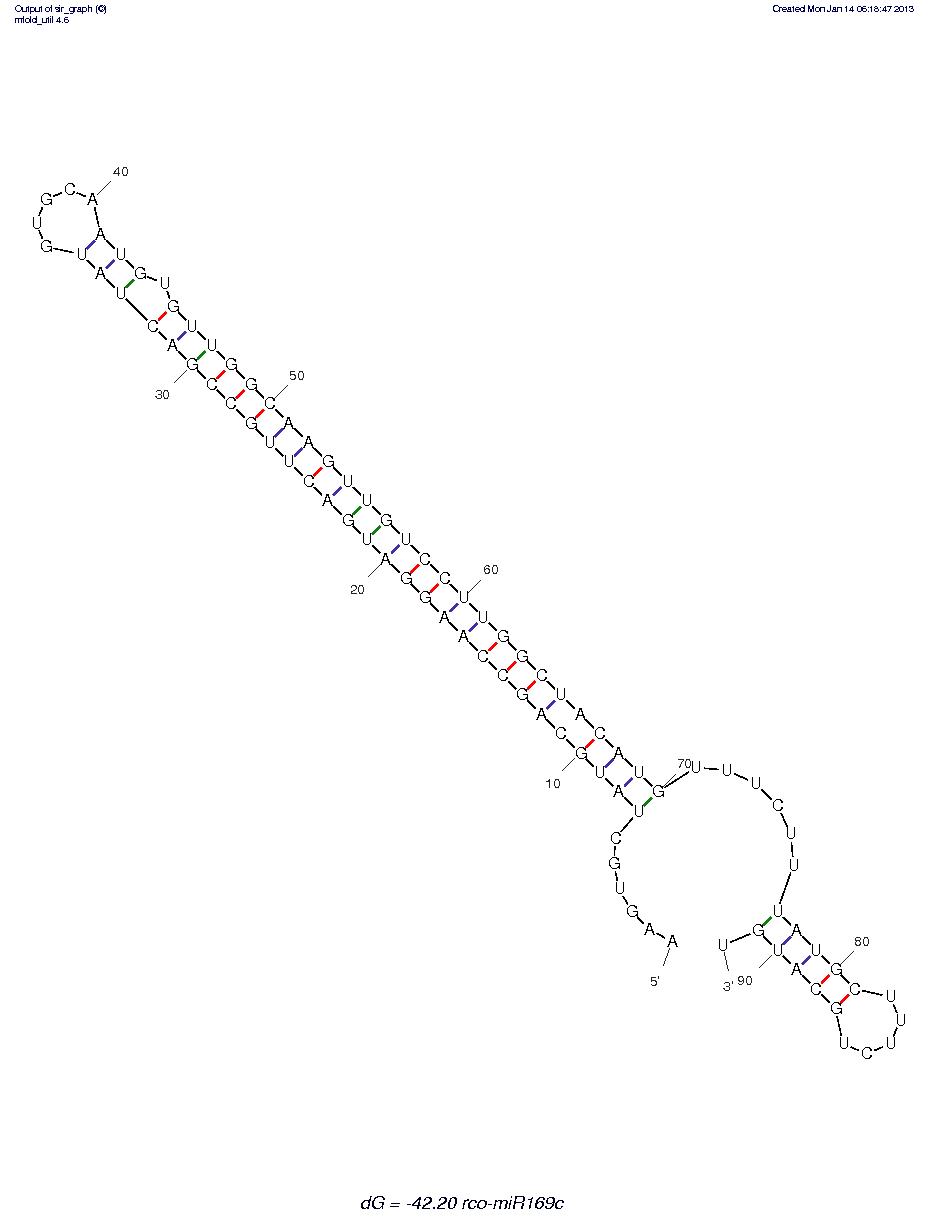


rco-miR169e*


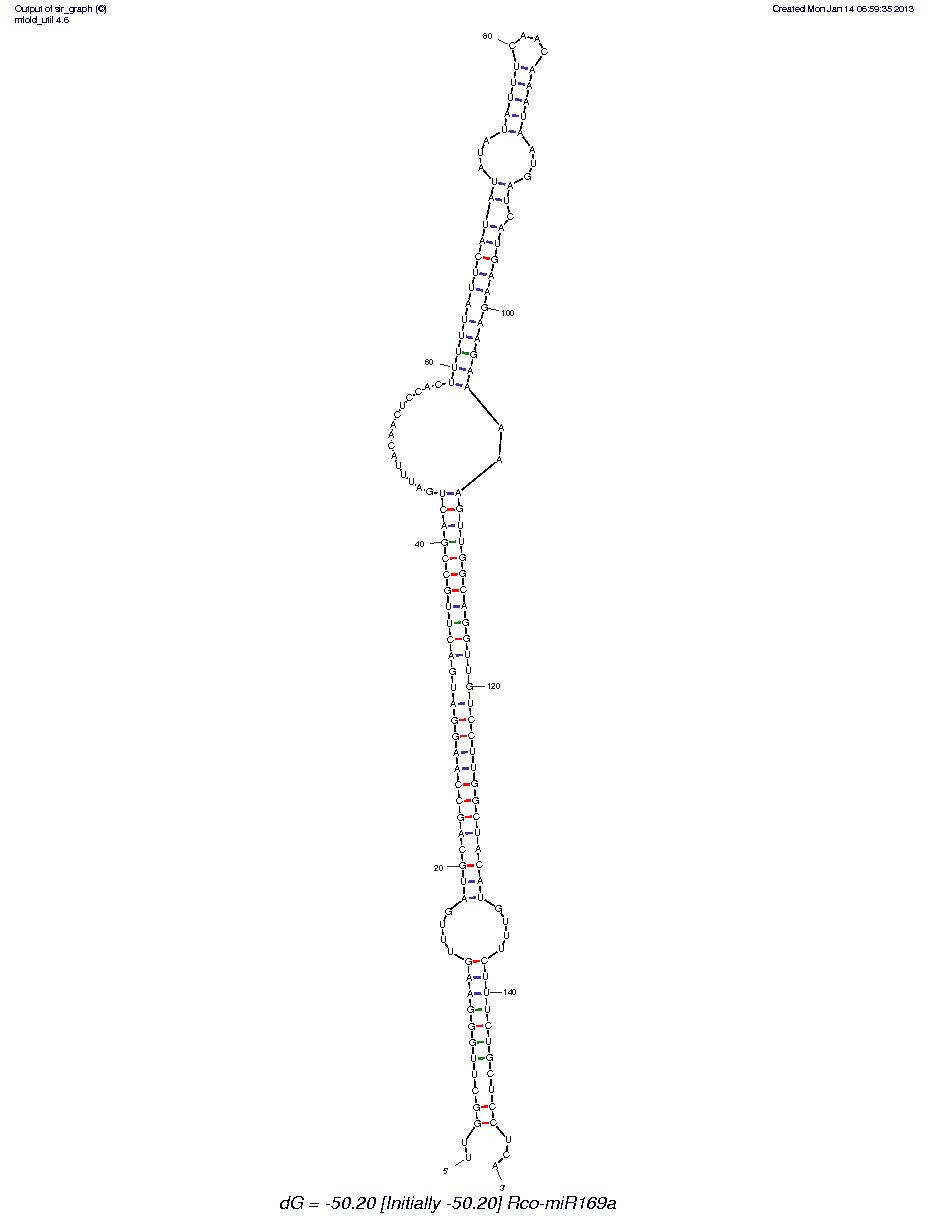


rco-miR169f*


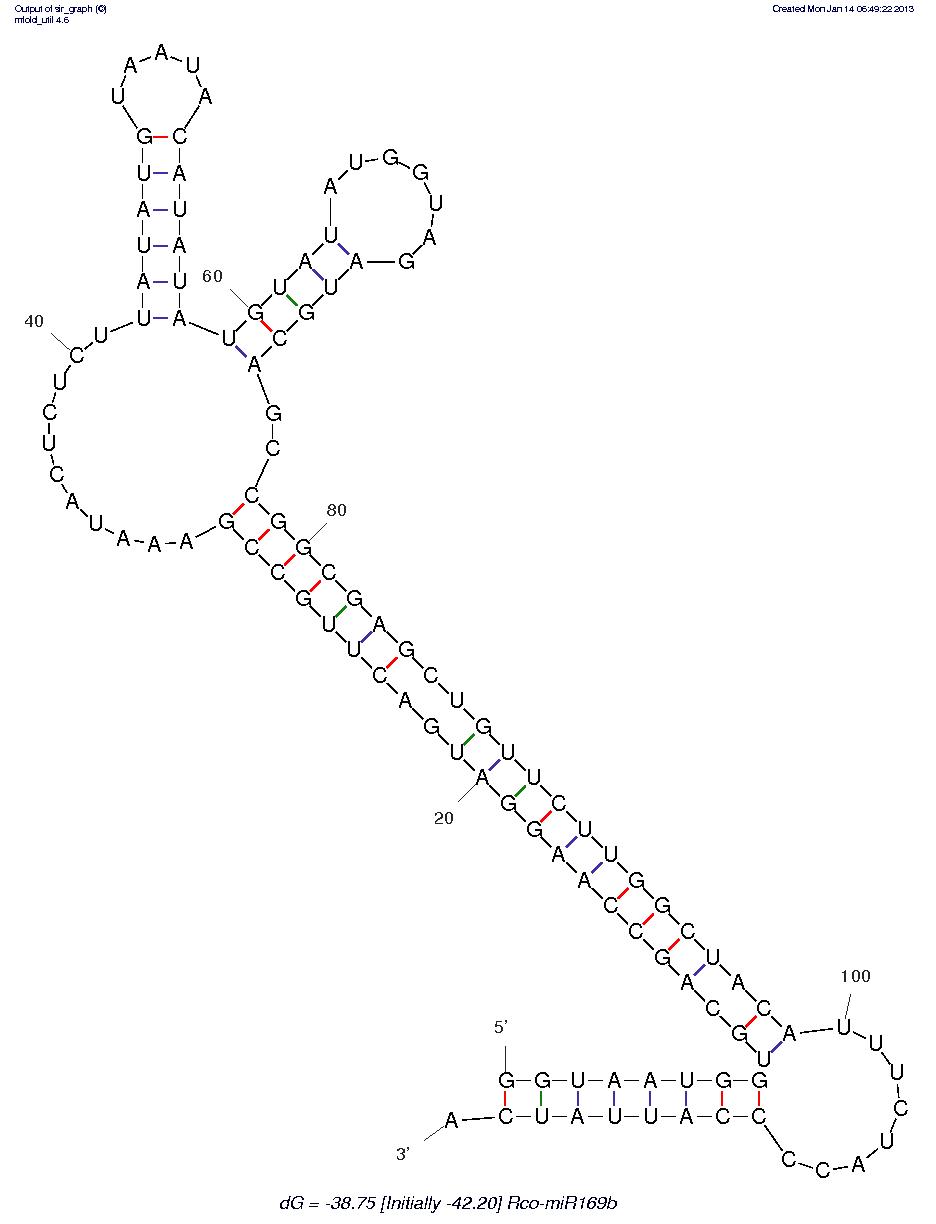


rco-miR169g*


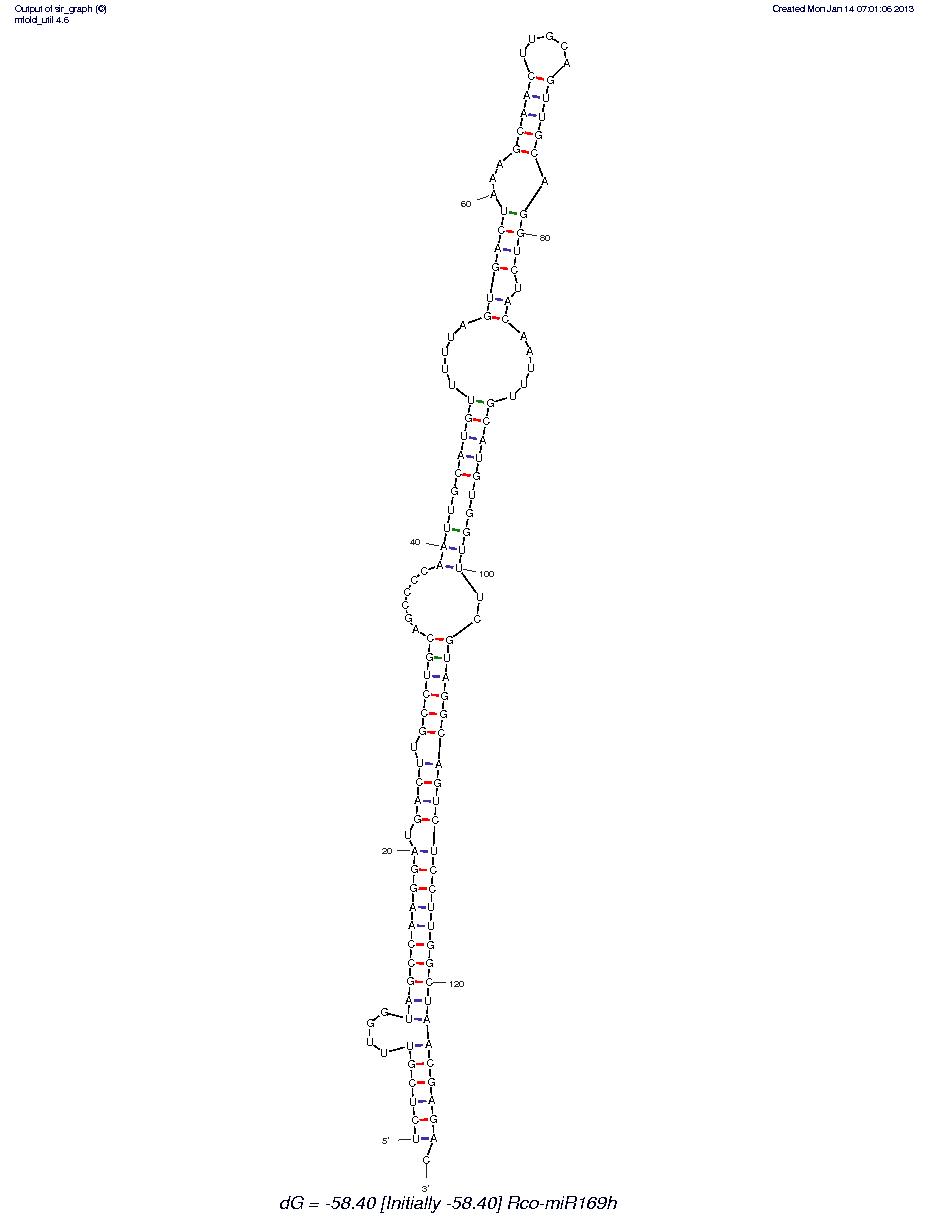


rco-miR169h*
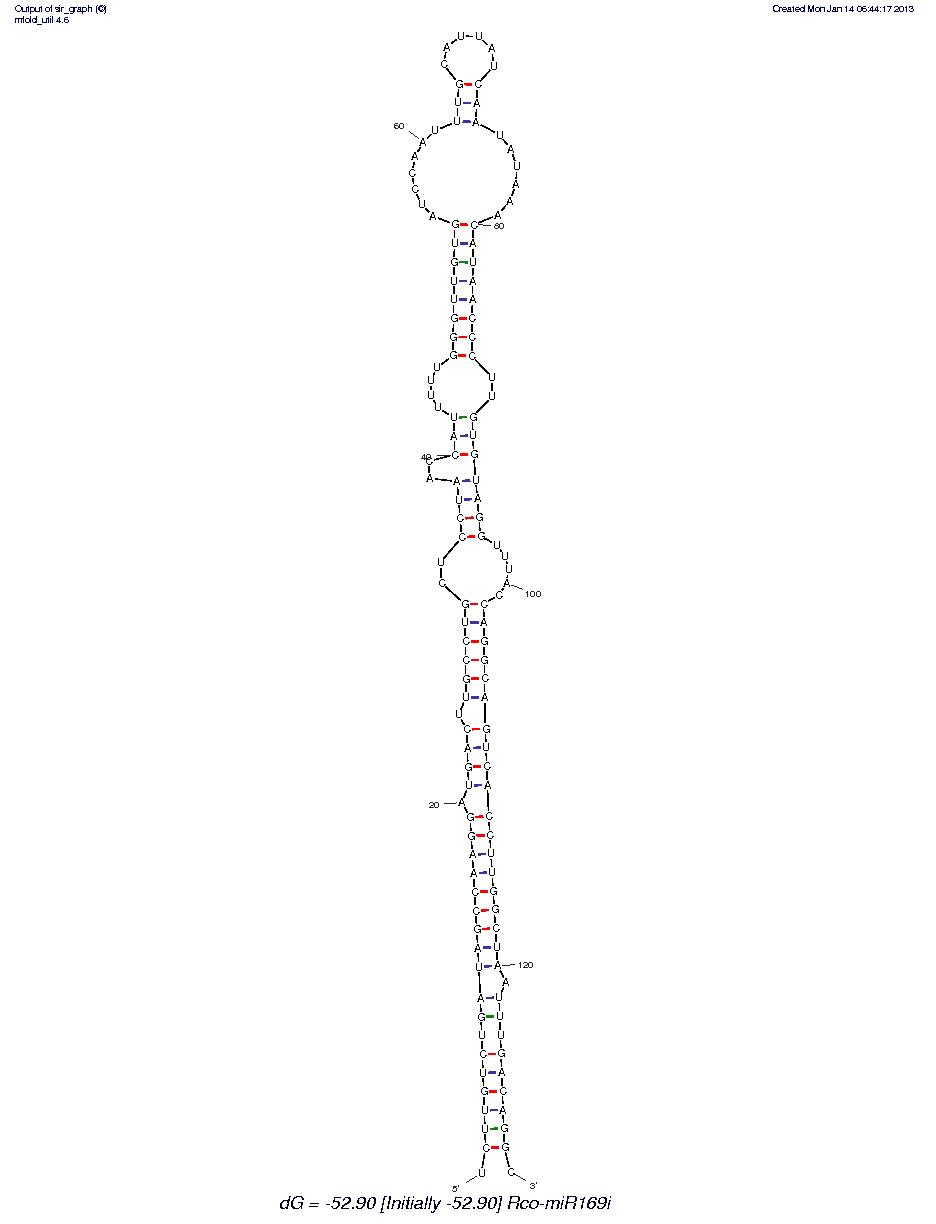


rco-miR169i*


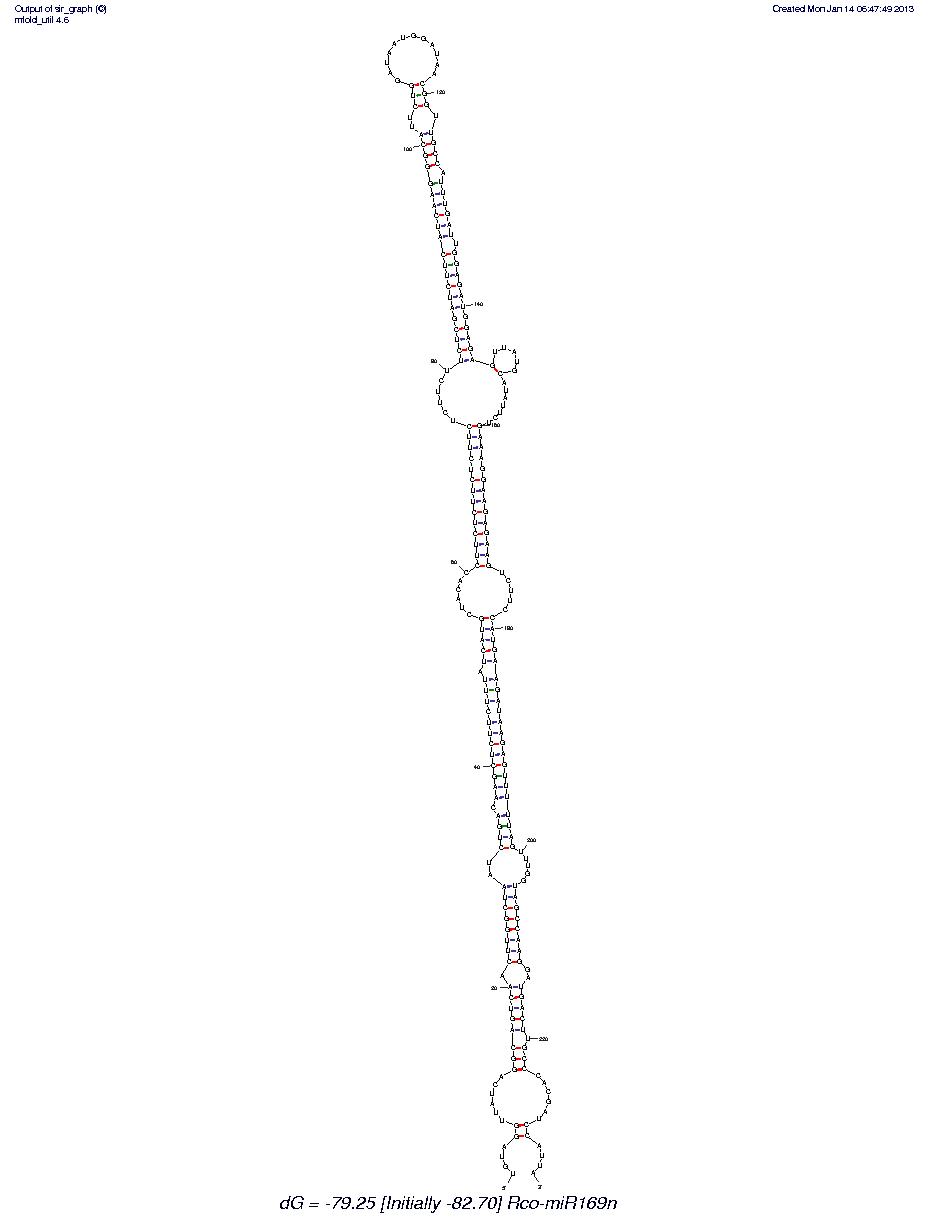


rco-miR169j*


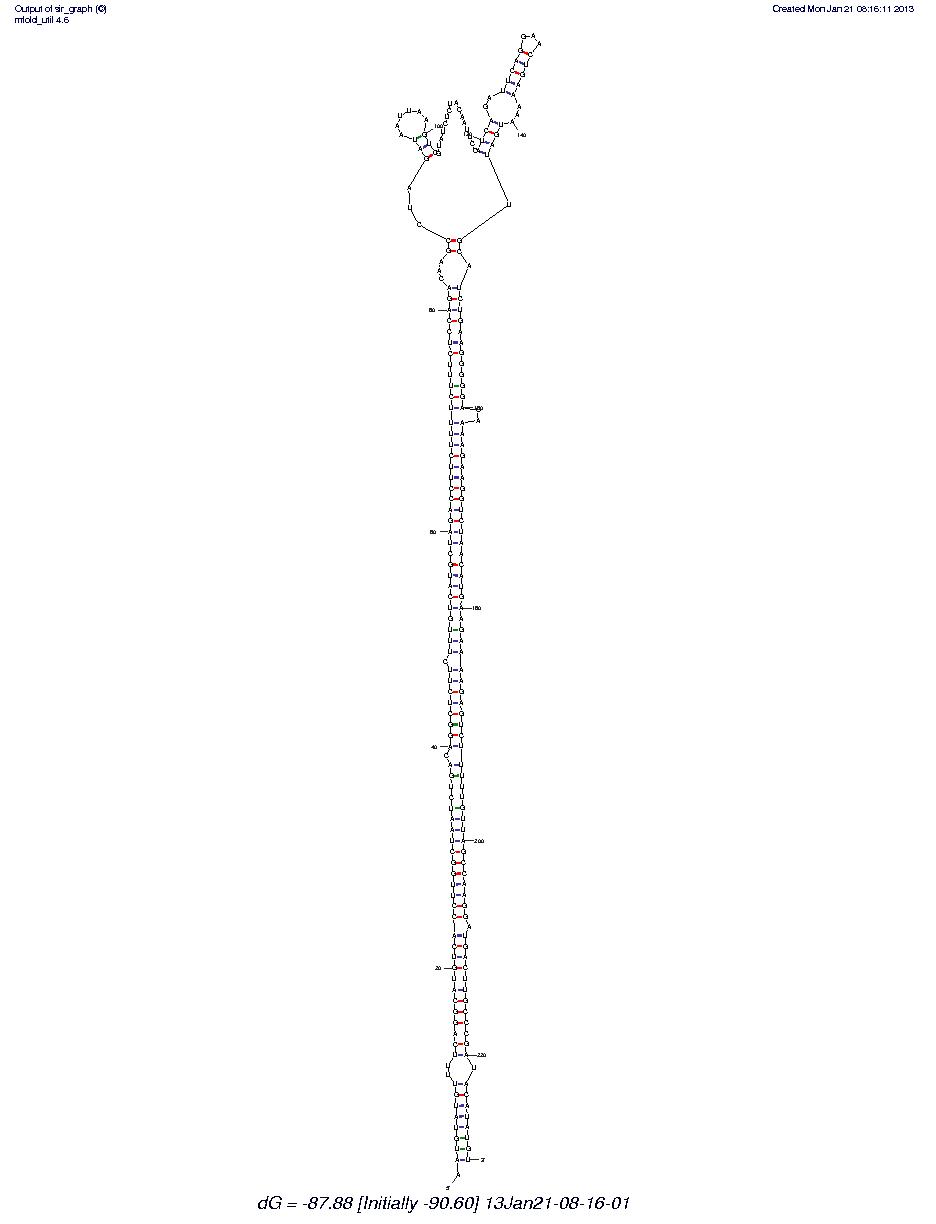


rco-miR169k*


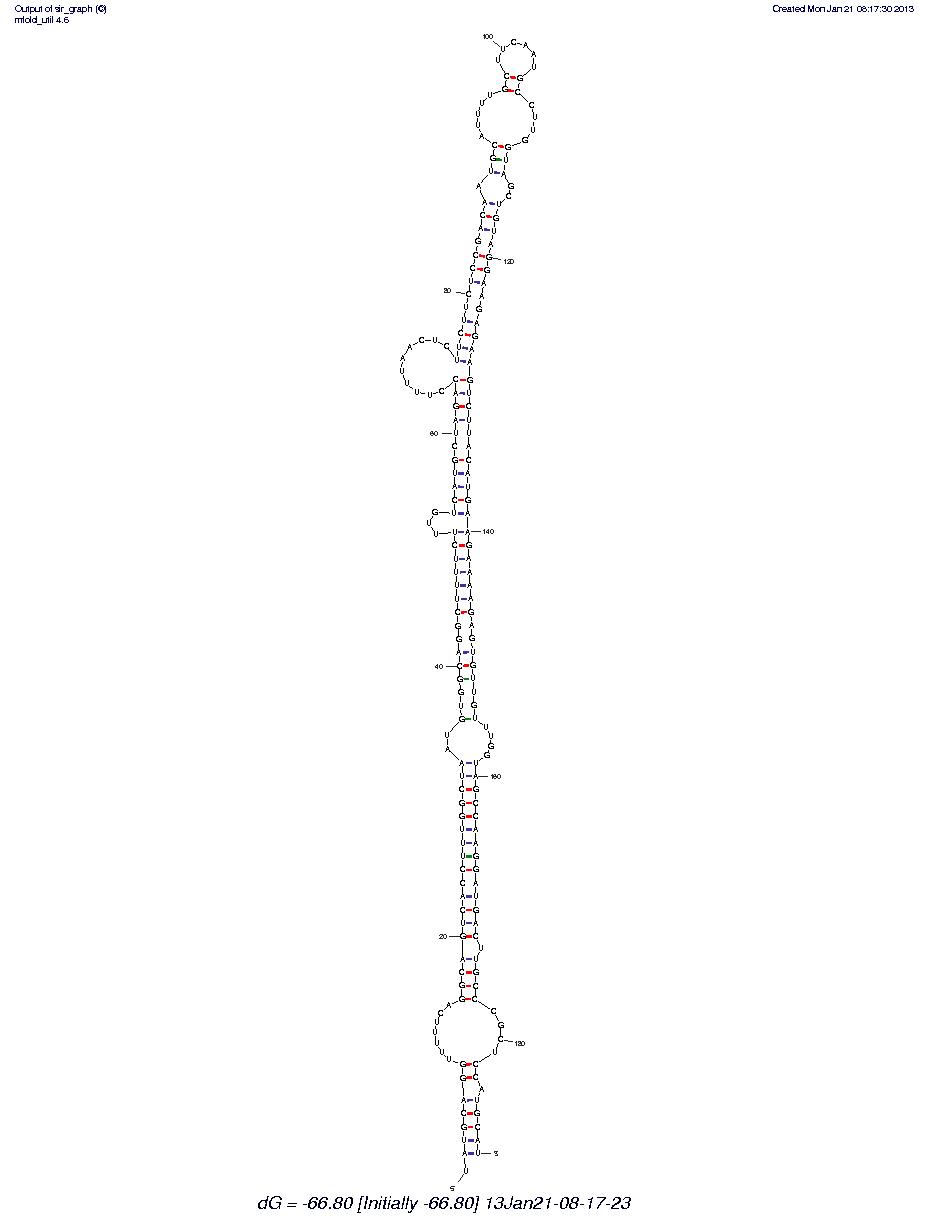


rco-miR169l*


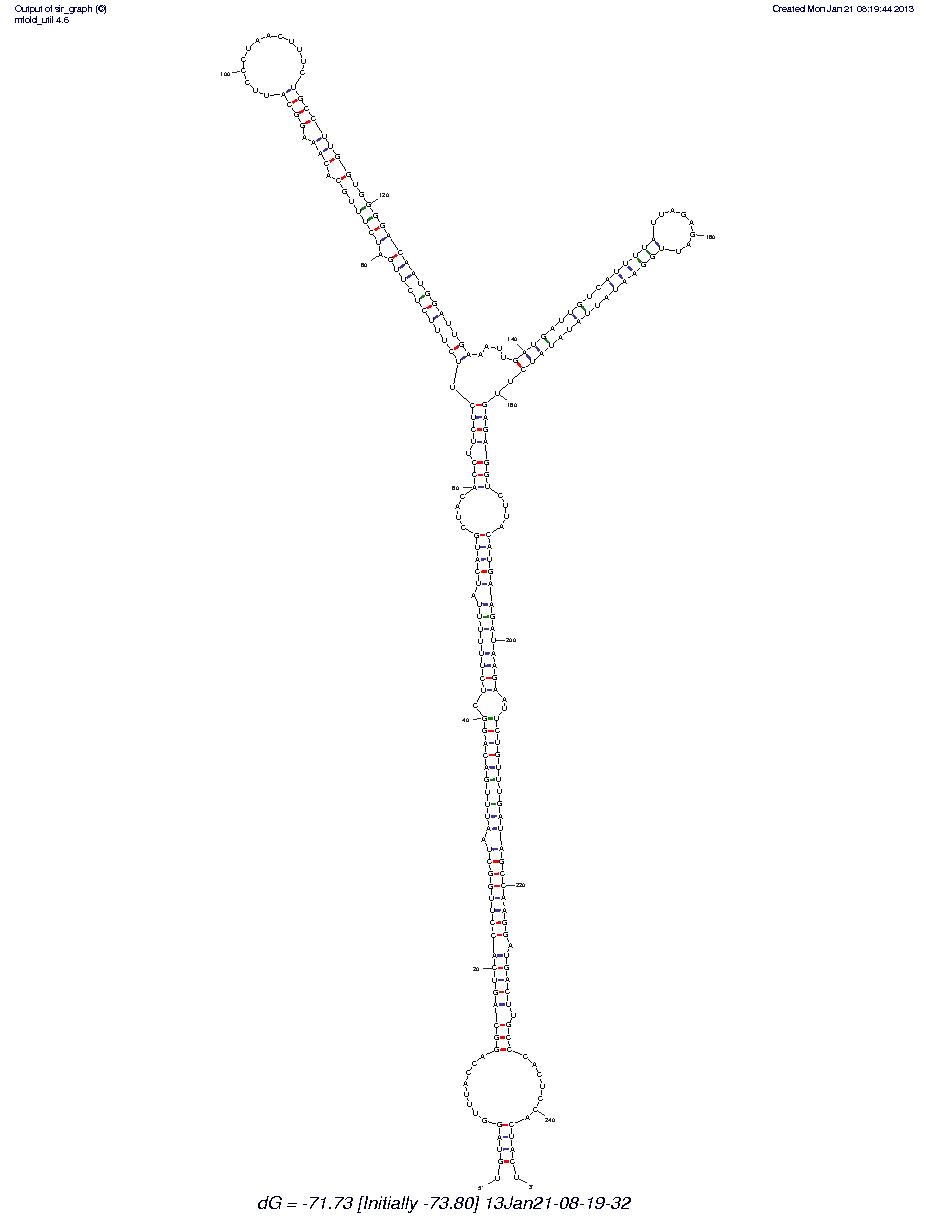


rco-miR171h*


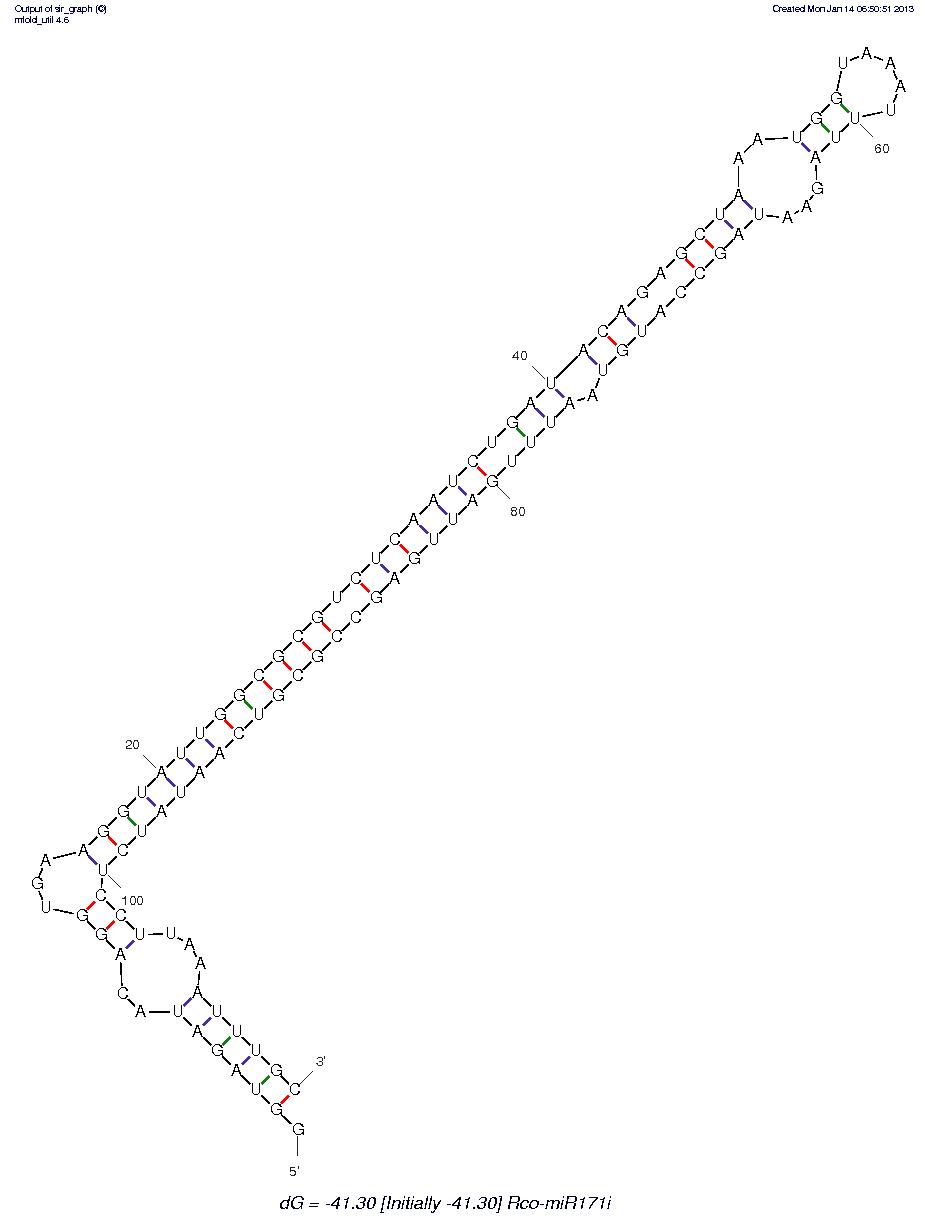


rco-miR171i*


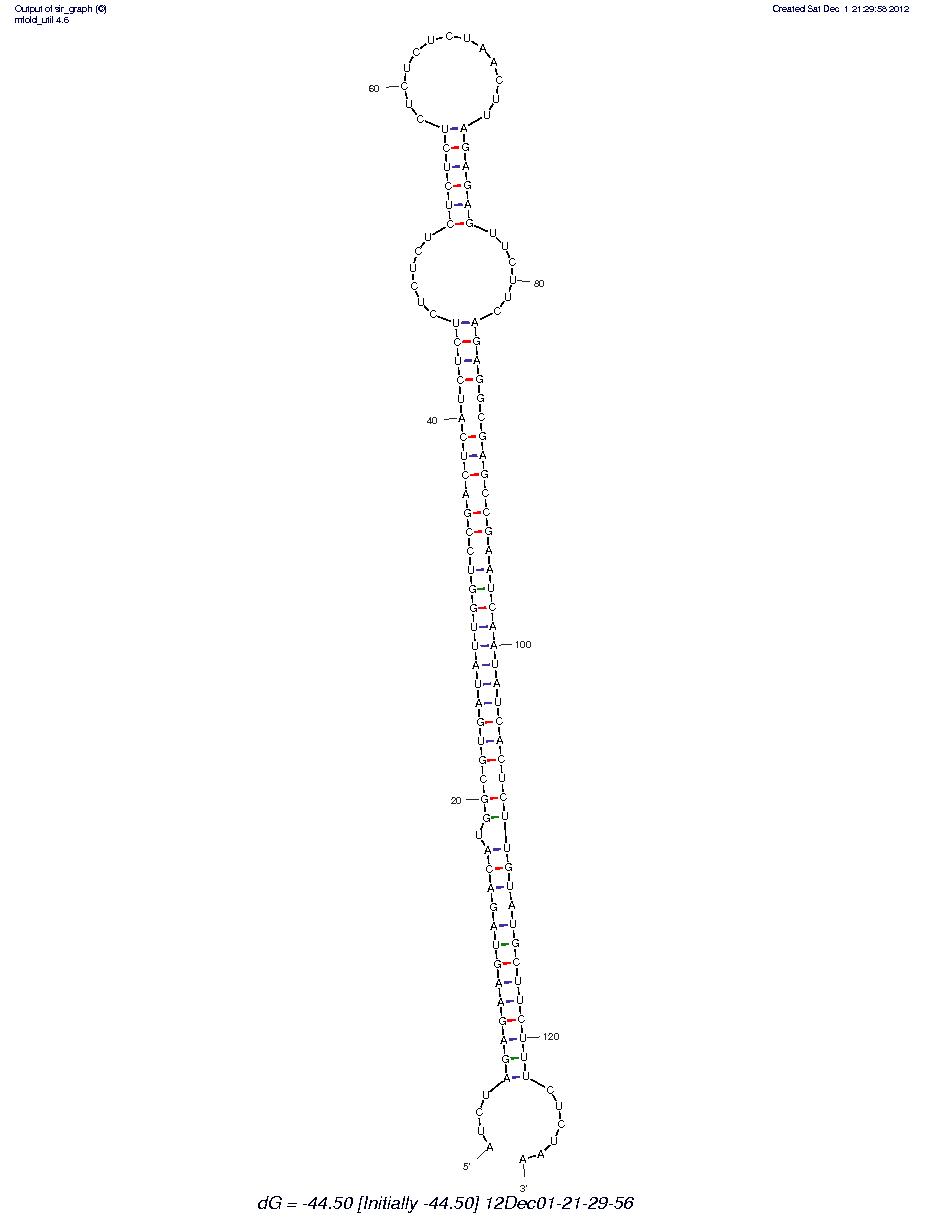


rco-miR172b*


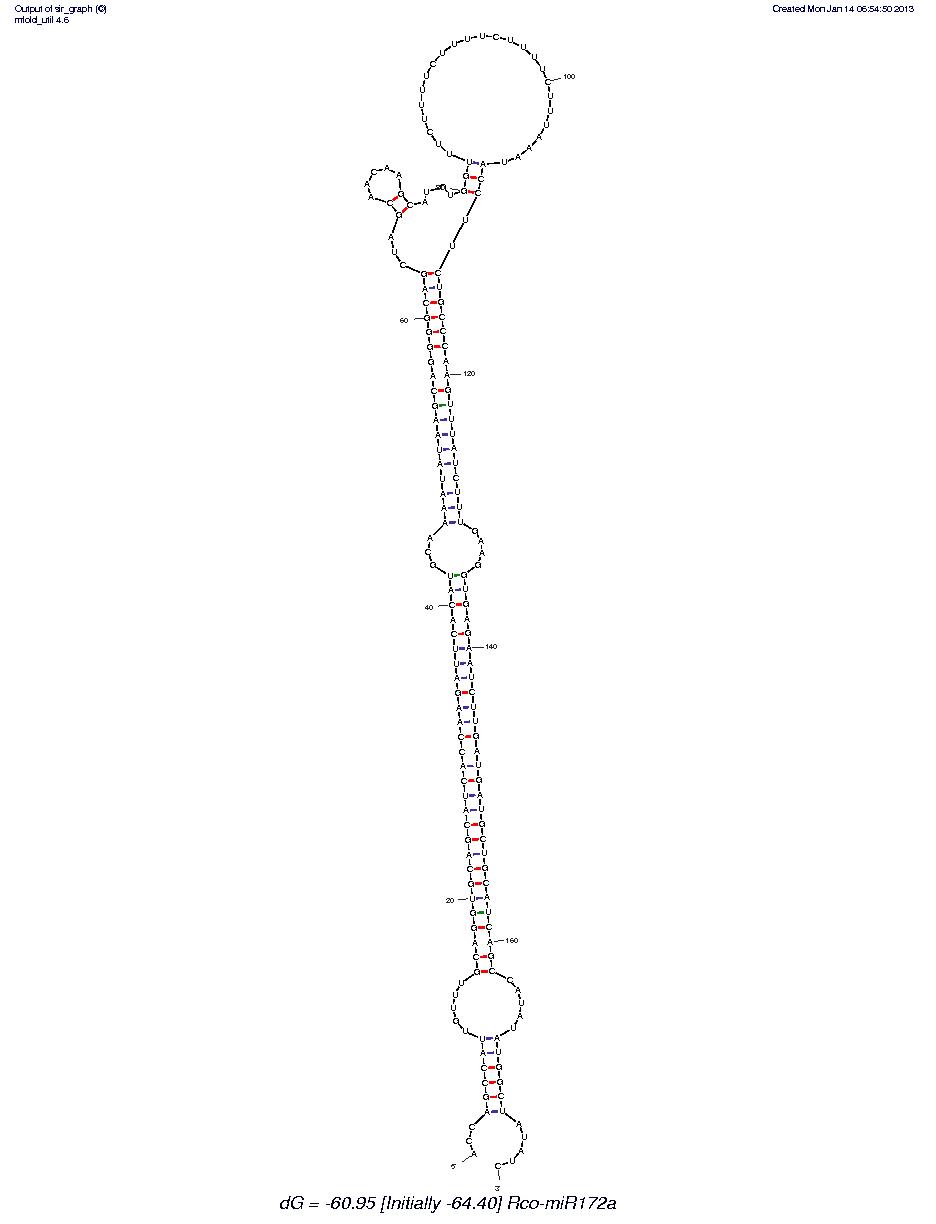


rco-miR172c*


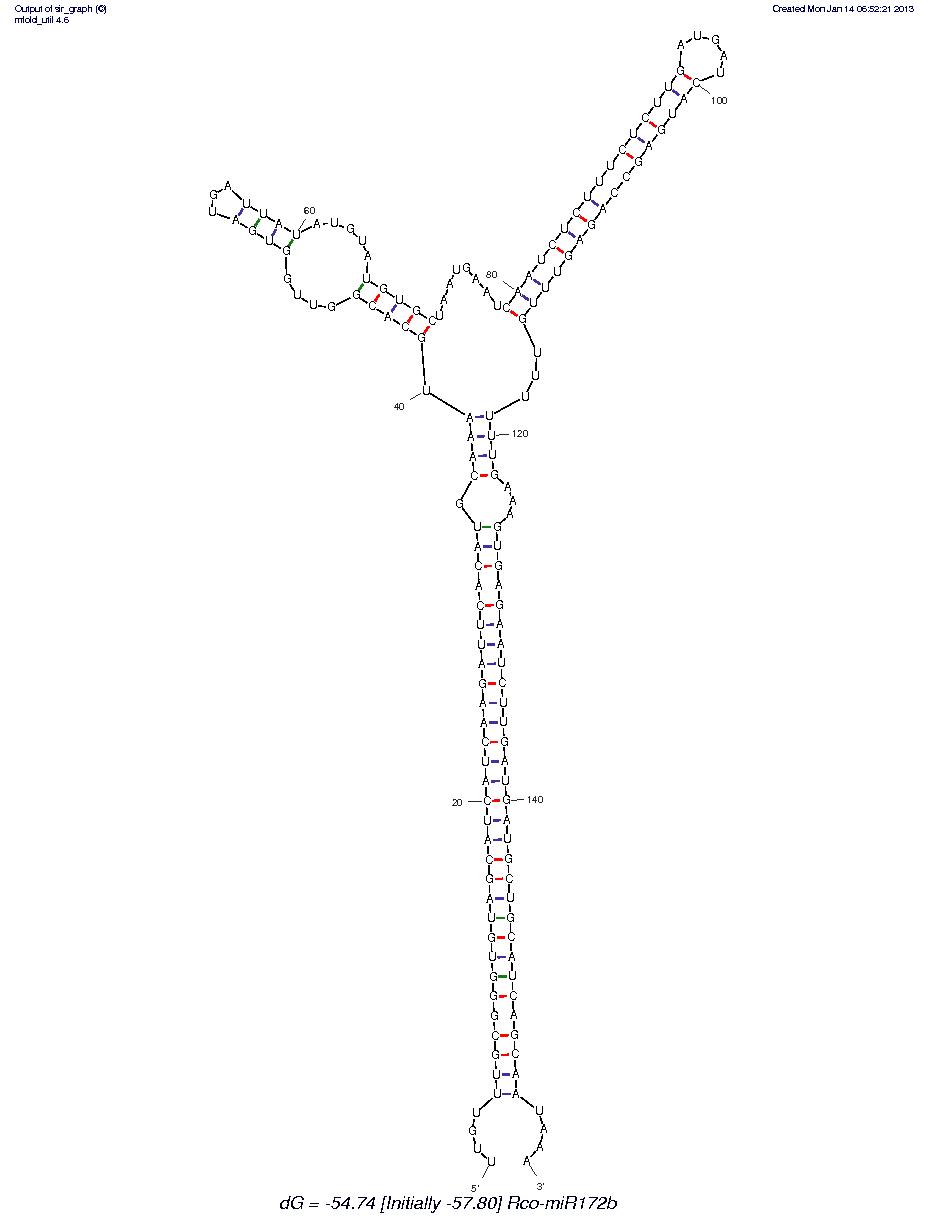


rco-miR172d*


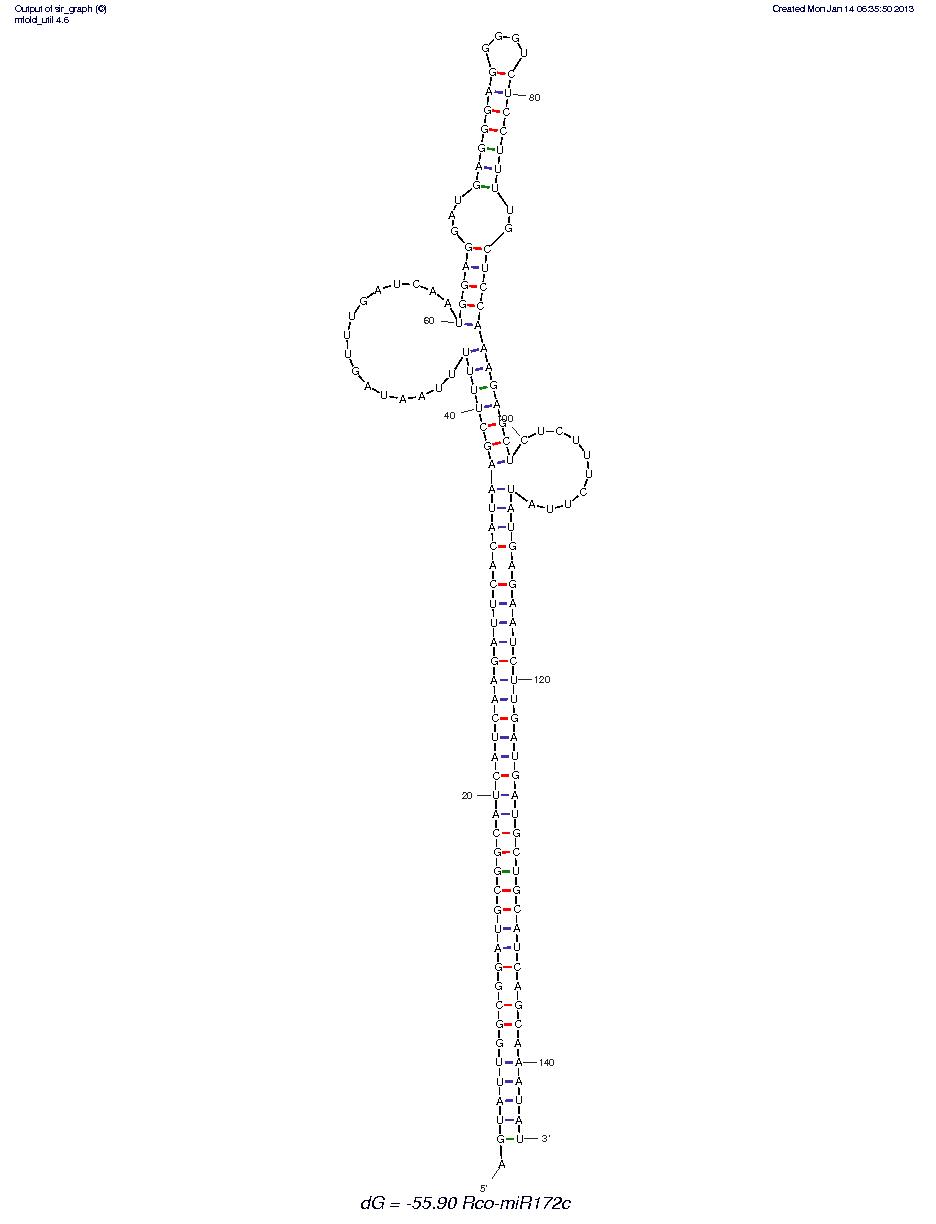


rco-miR393b*


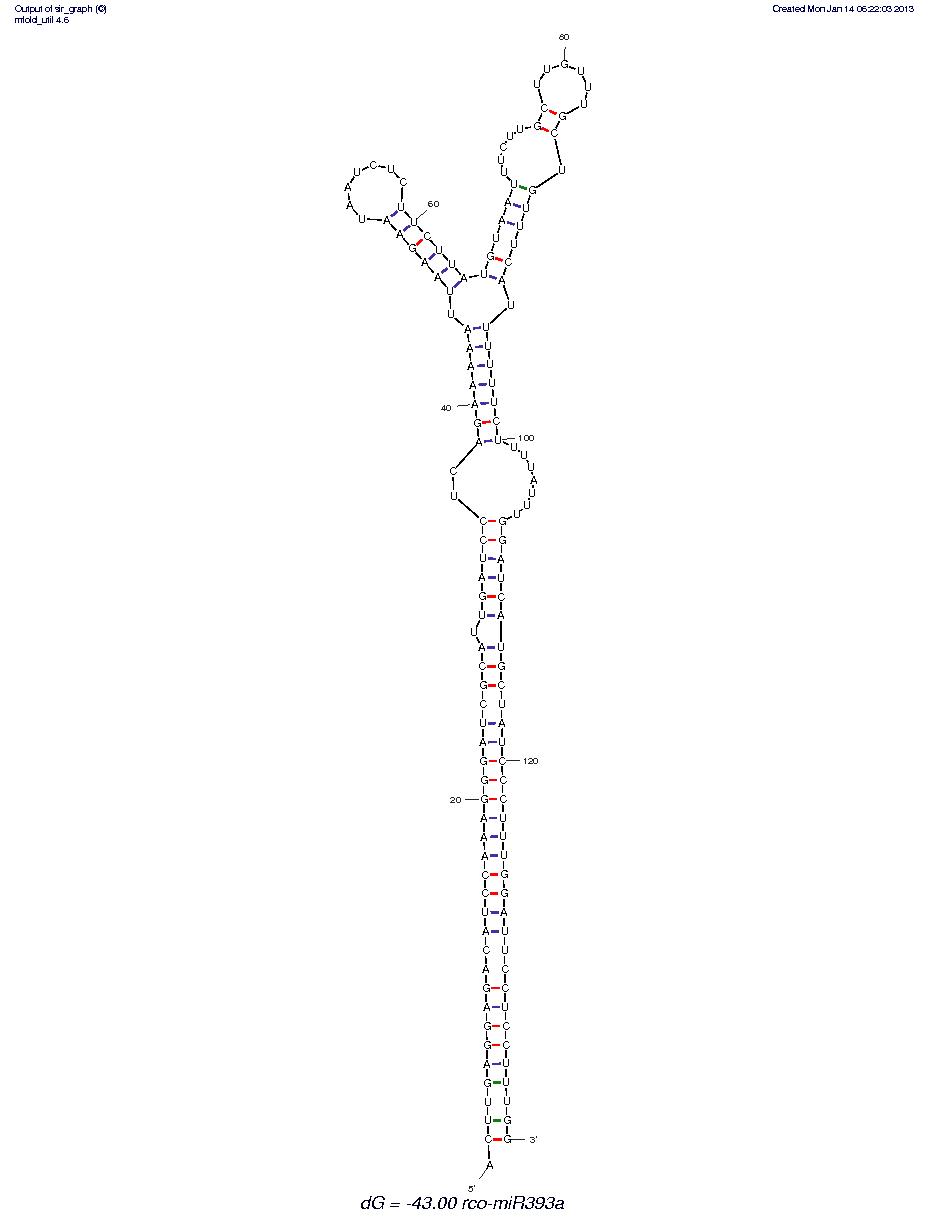


rco-miR394a*


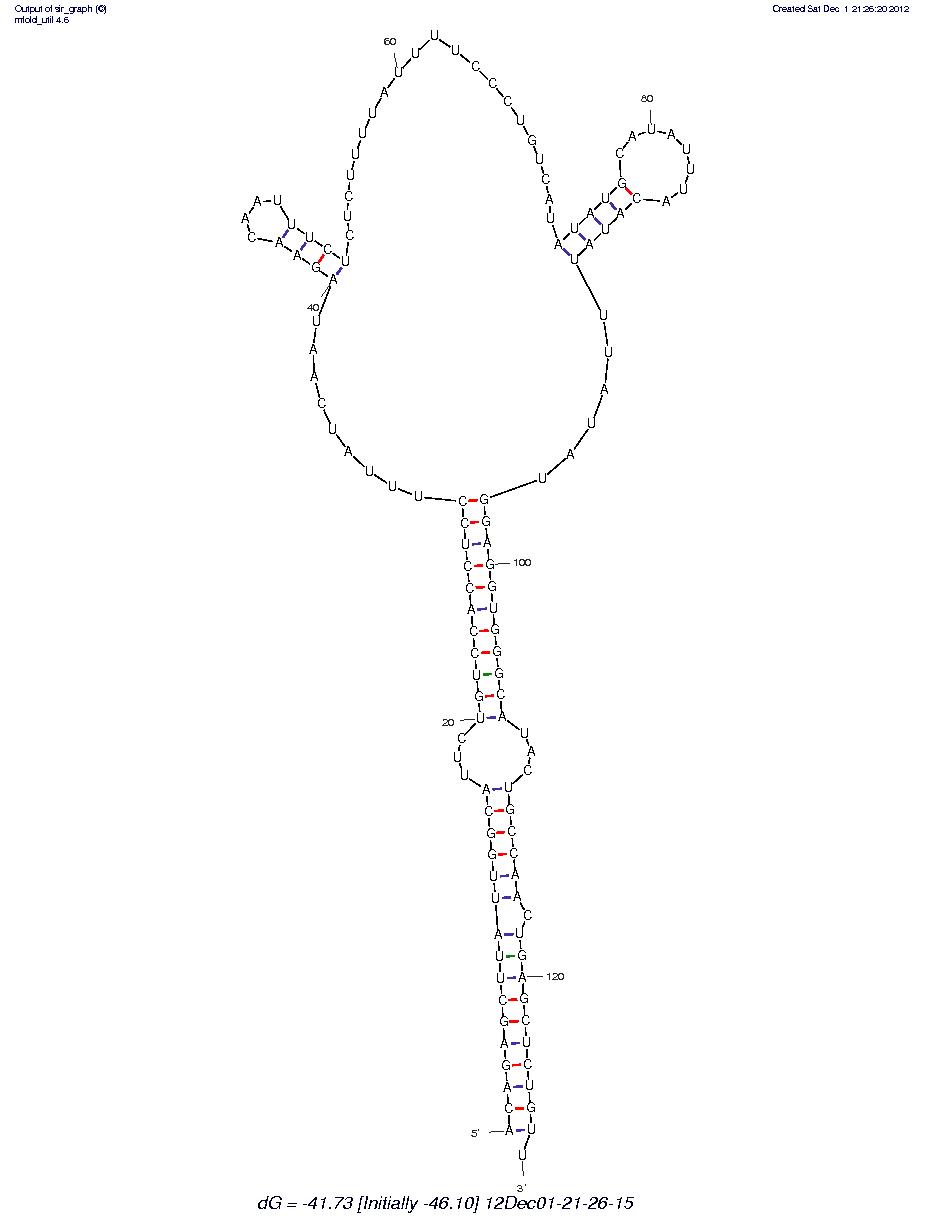


rco-miR394b*


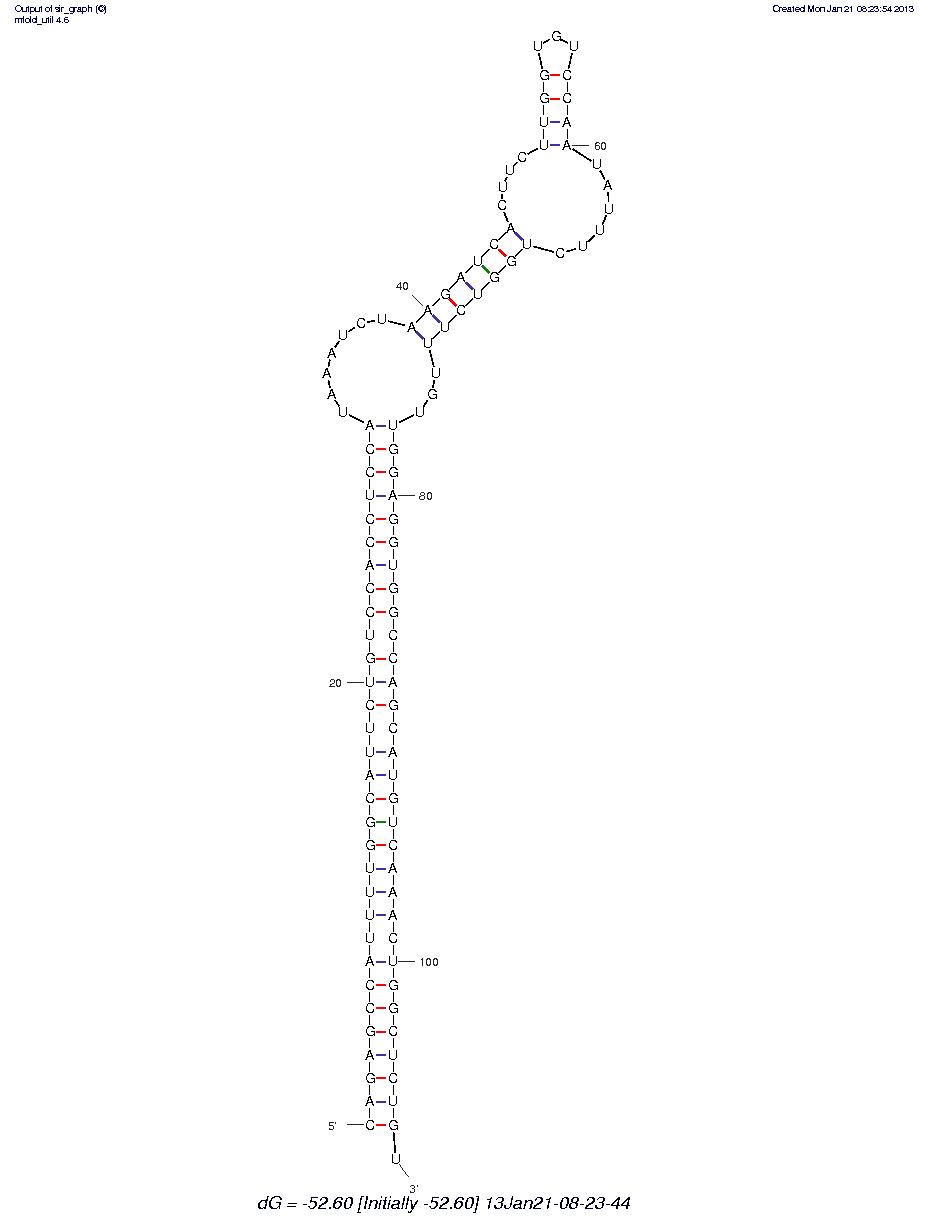


rco-miR396b*


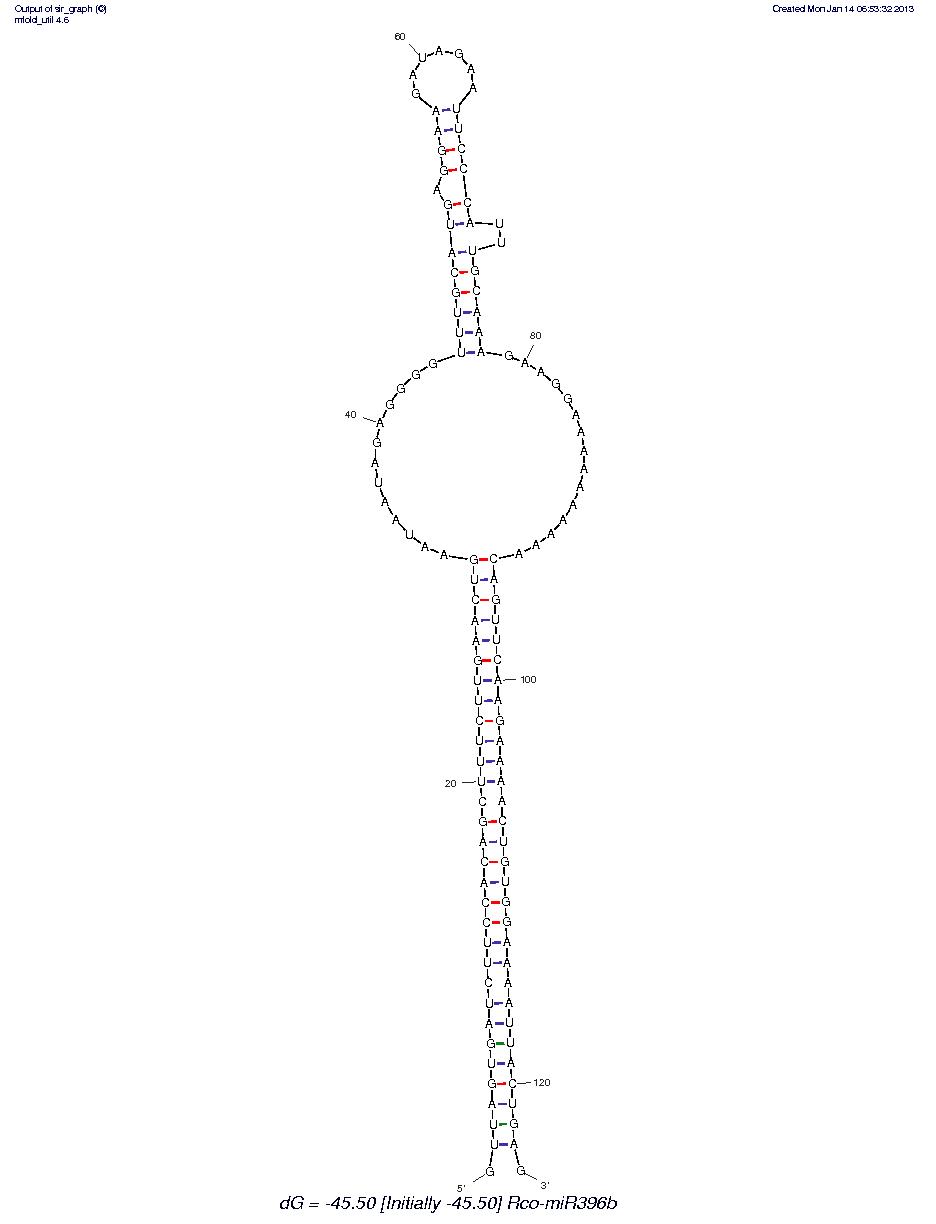


rco-miR396c*


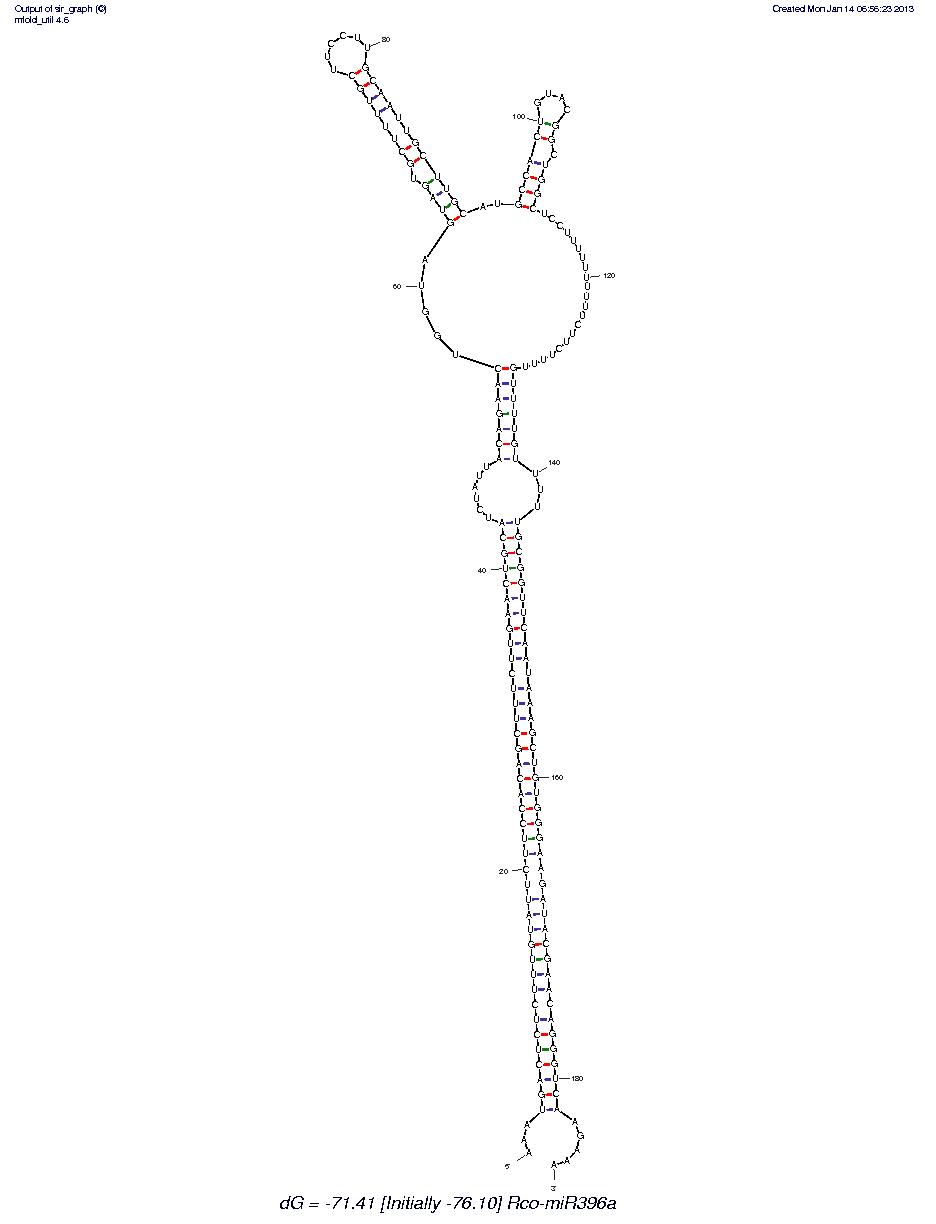


rco-miR482*


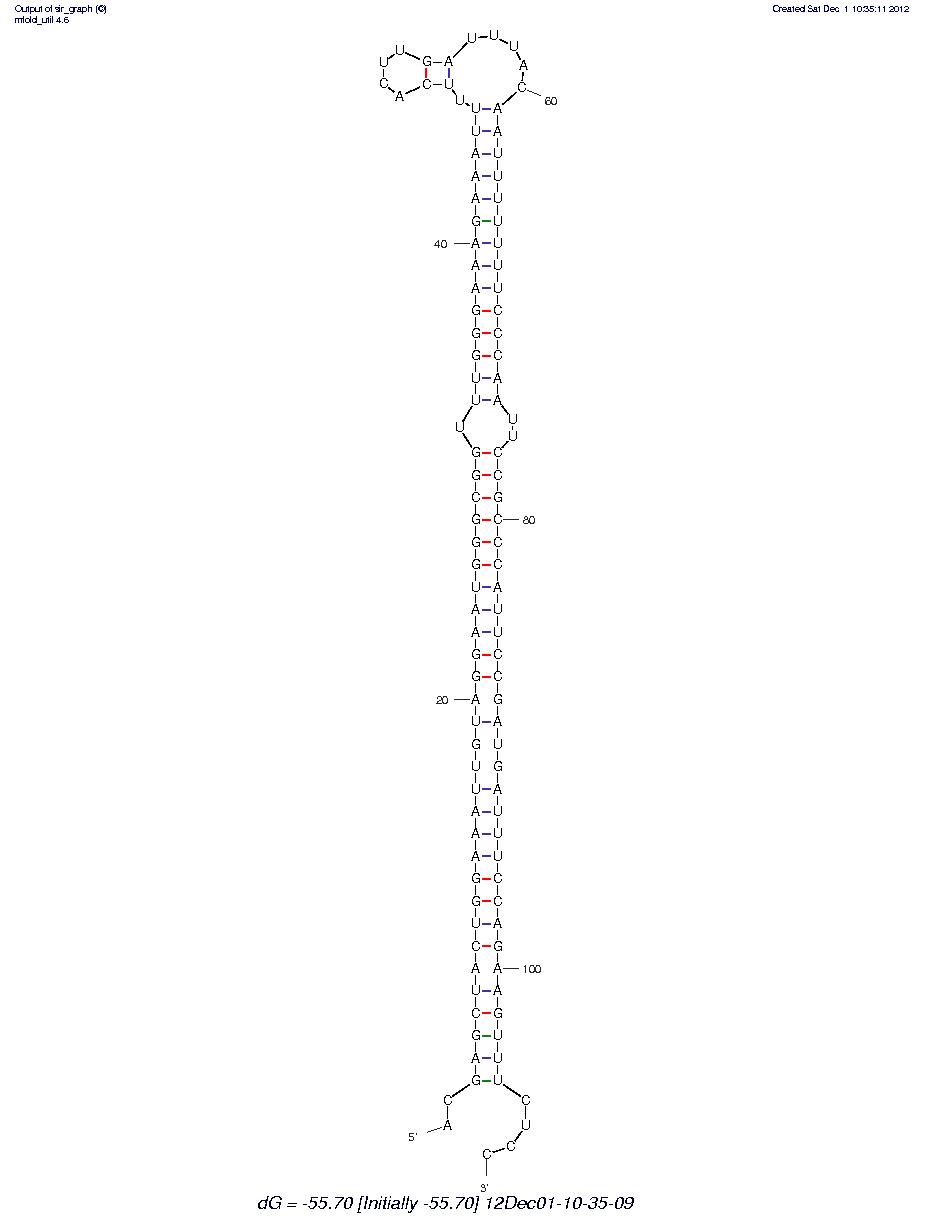


rco-miR827*


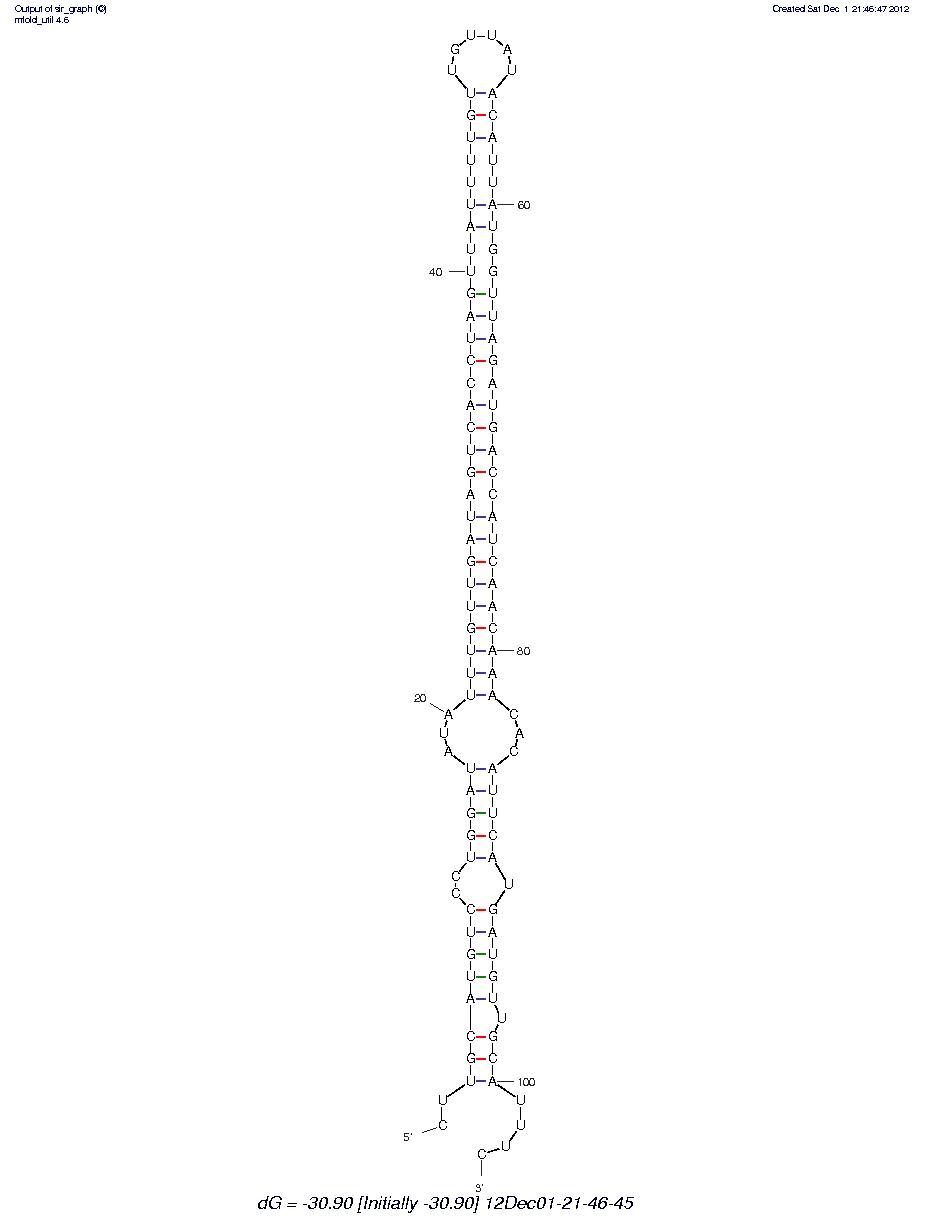


rco-miR2111*


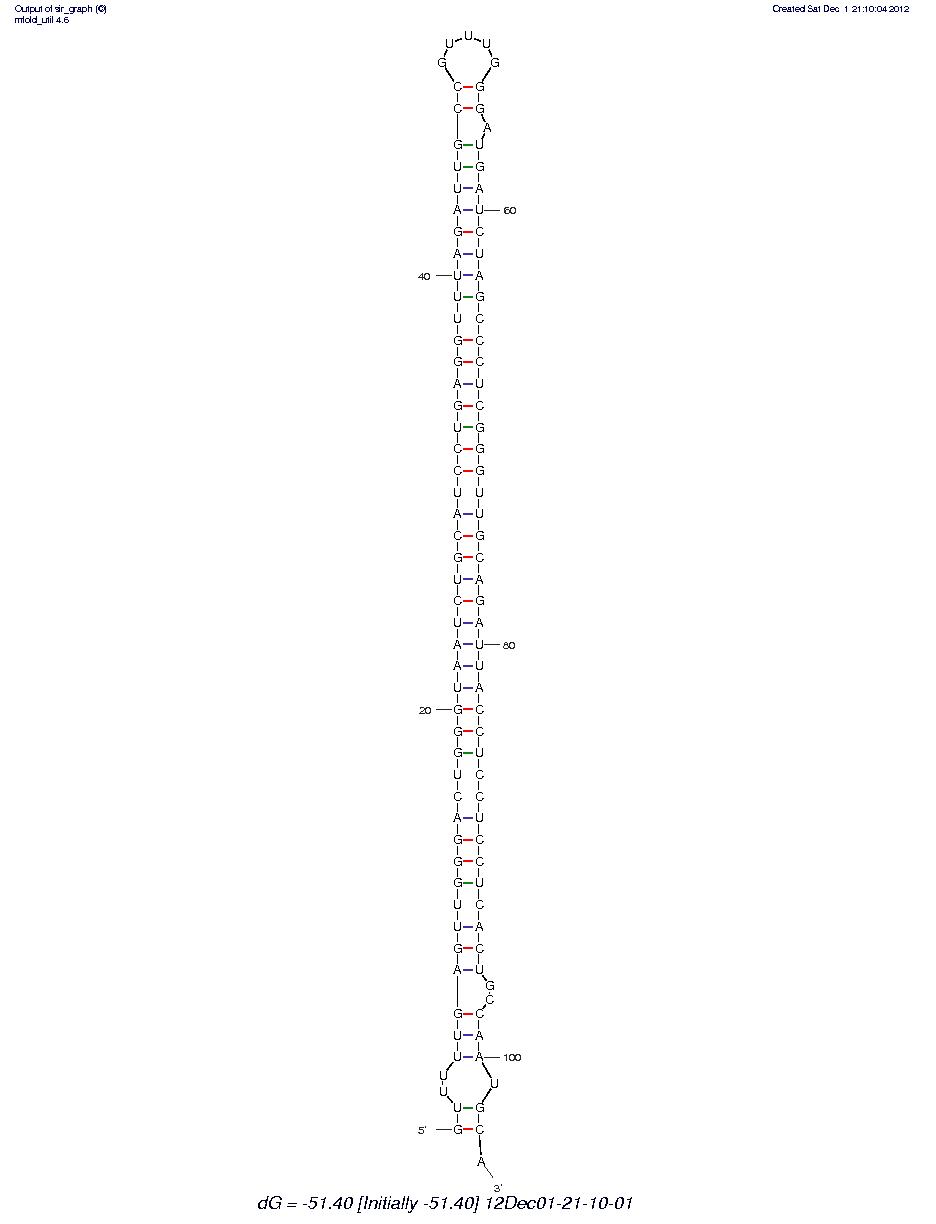


rco-miR4414*
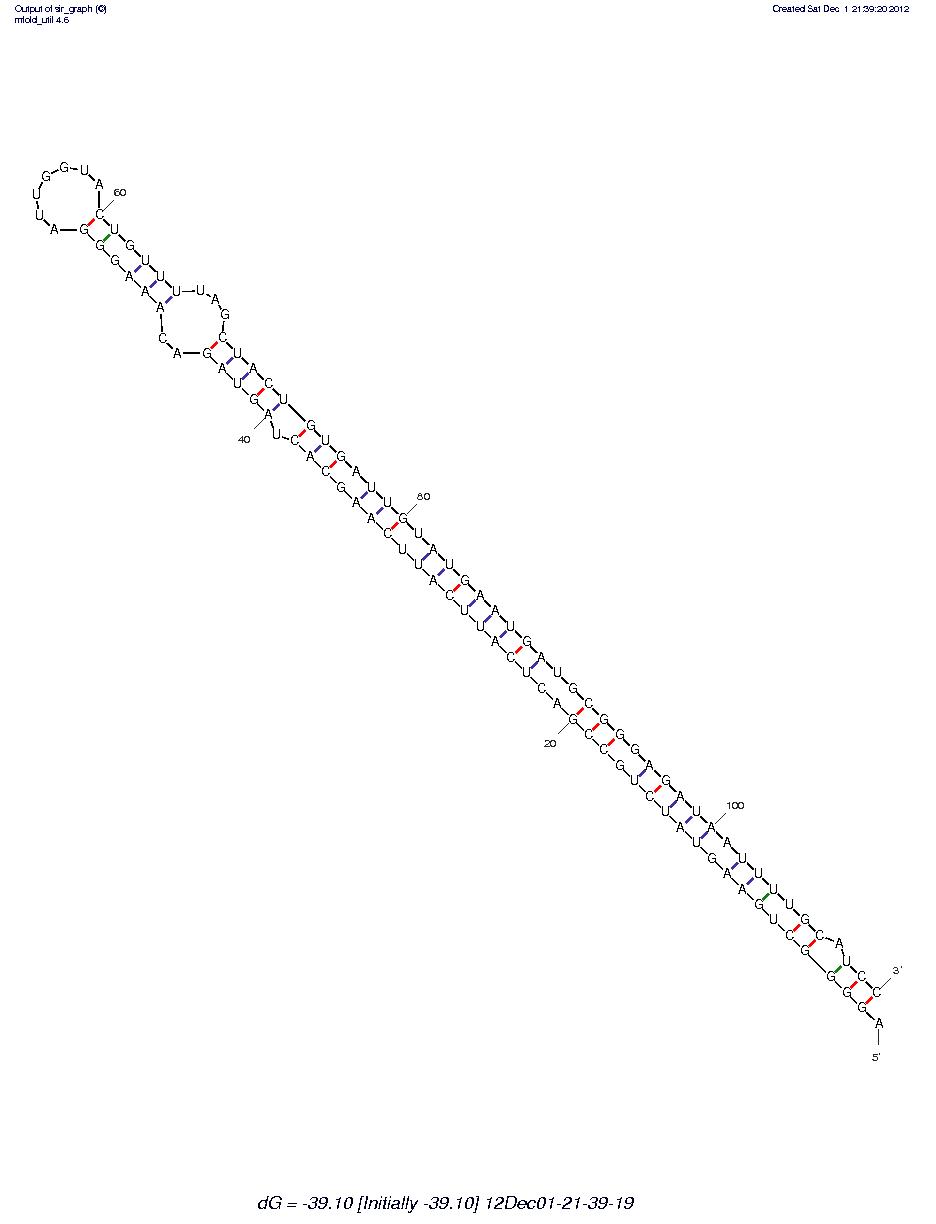


rcomiR001


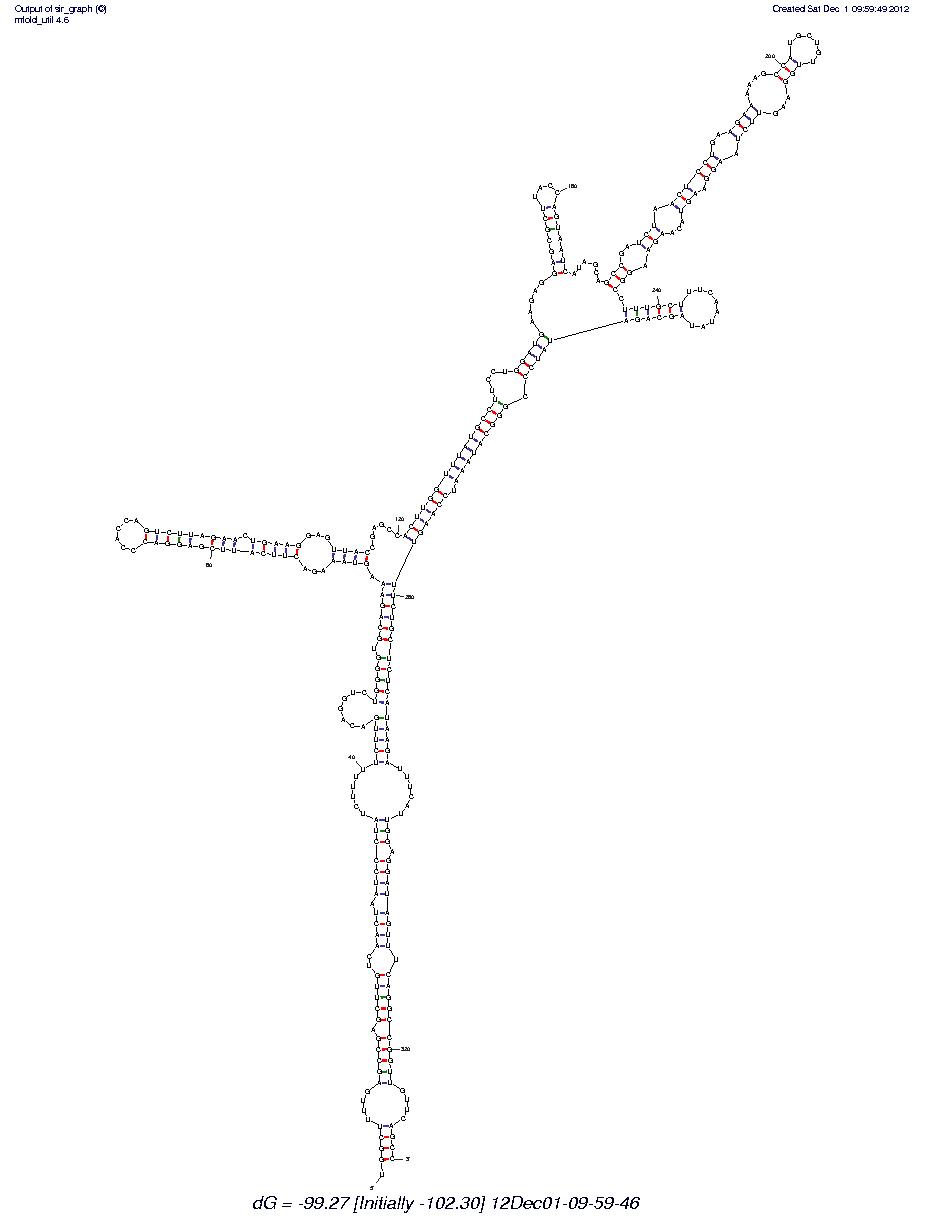


rcomiR002


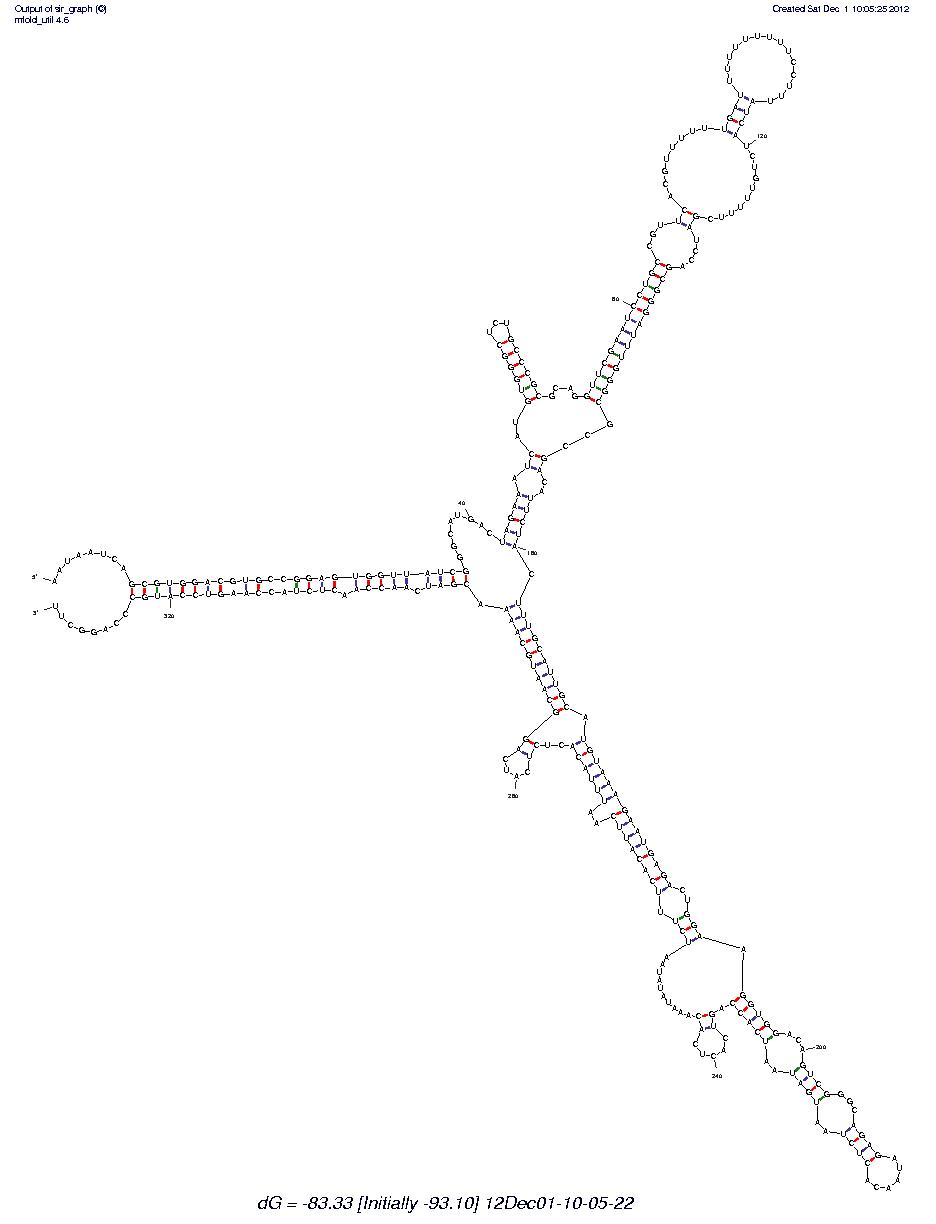


rcomiR003


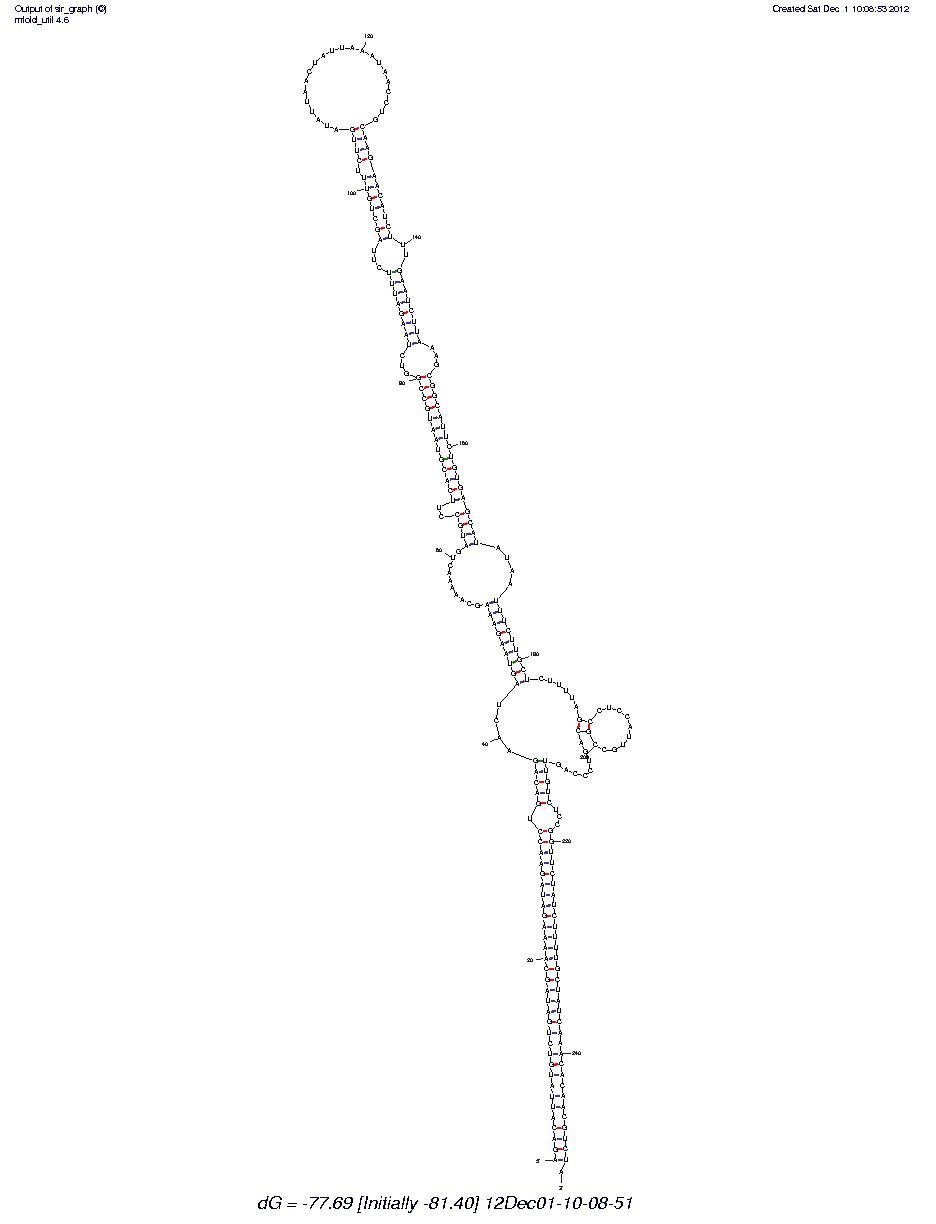


rcomiR004


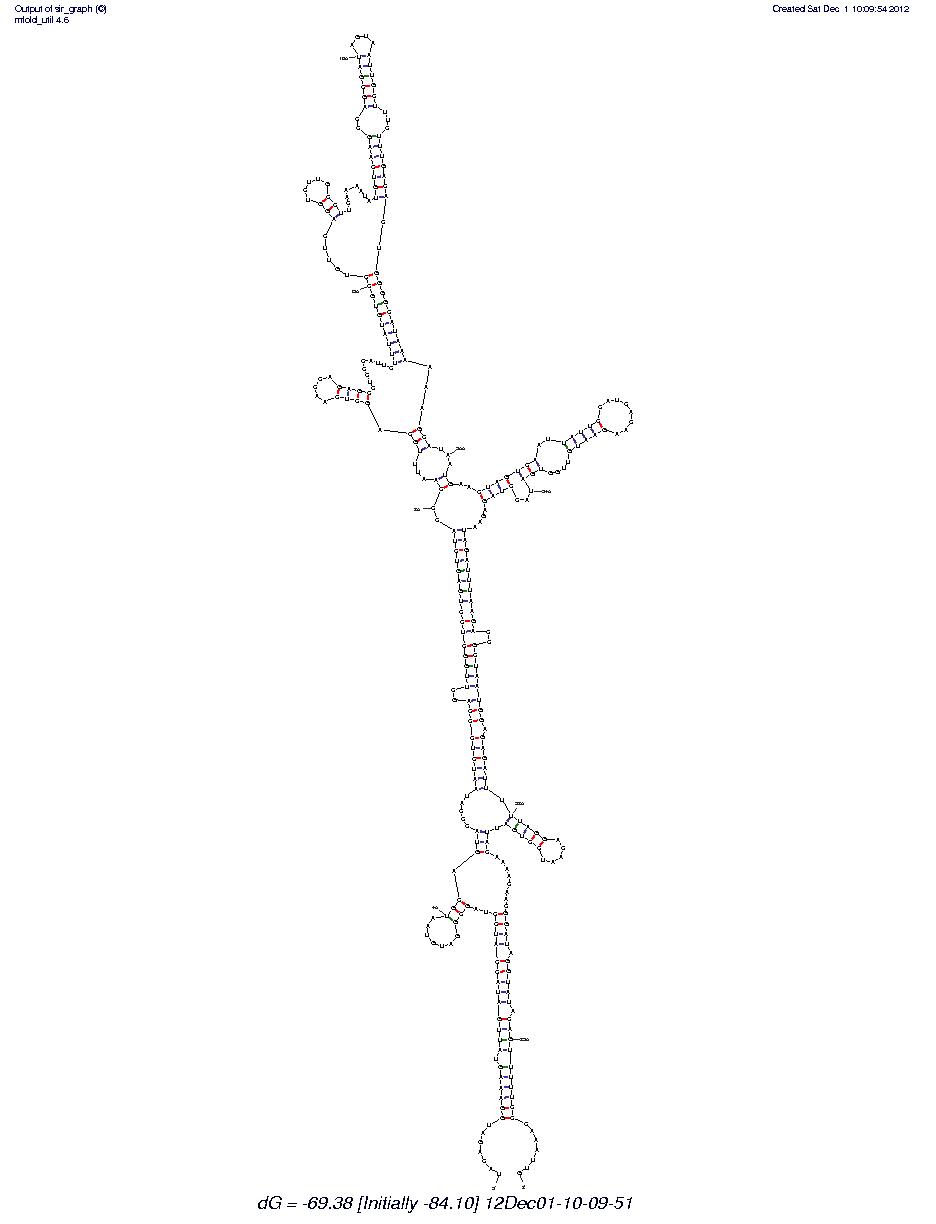


rcomiR005
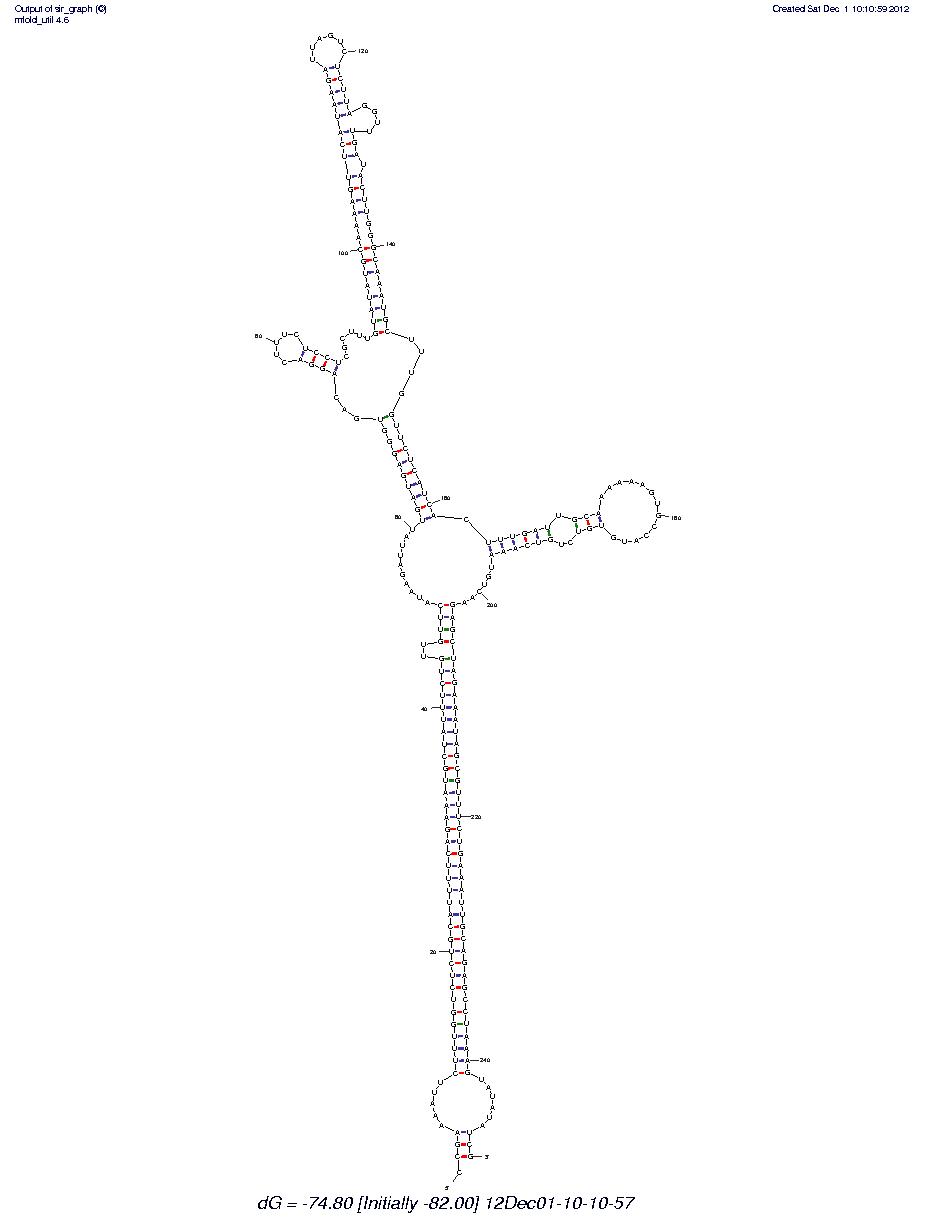


rcomiR006


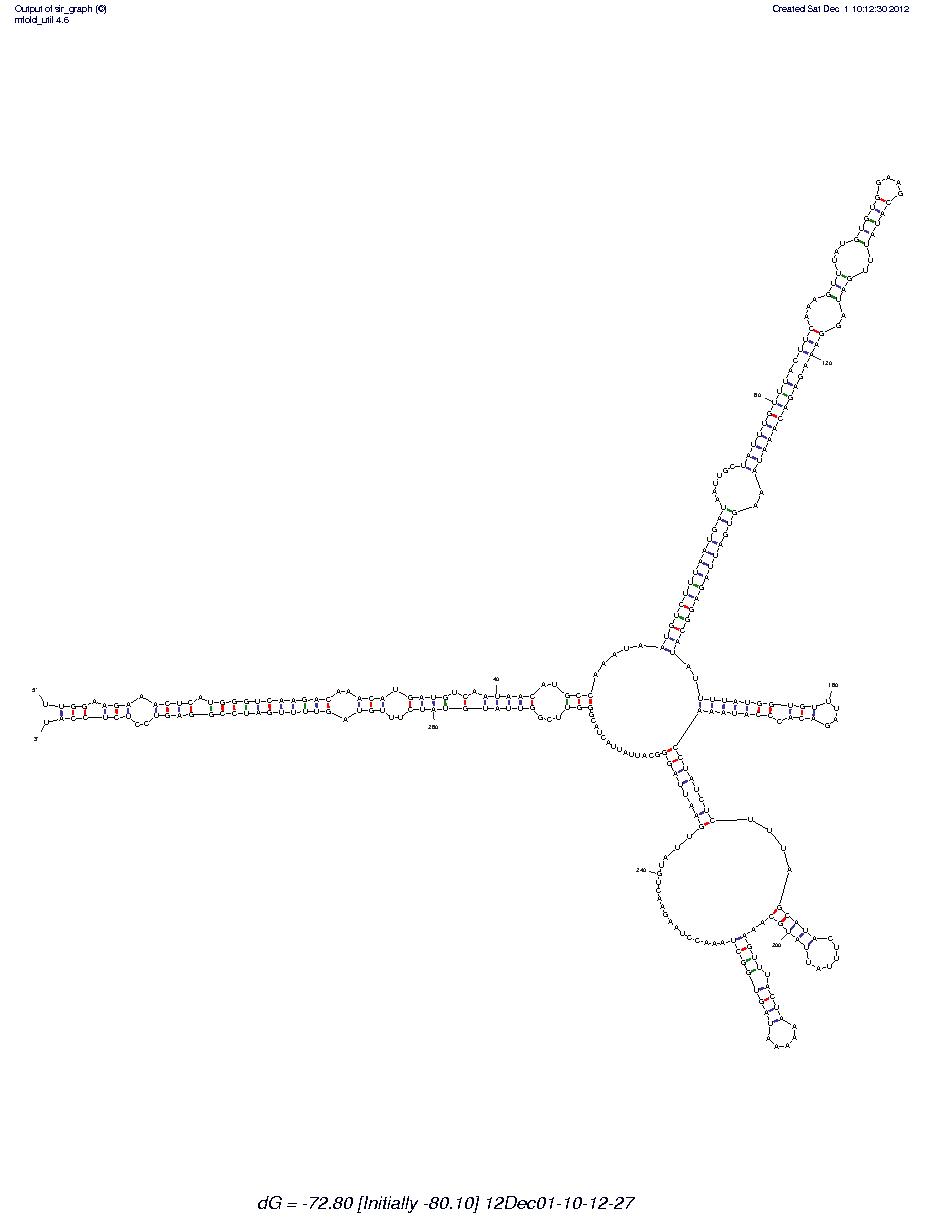


rcomiR007


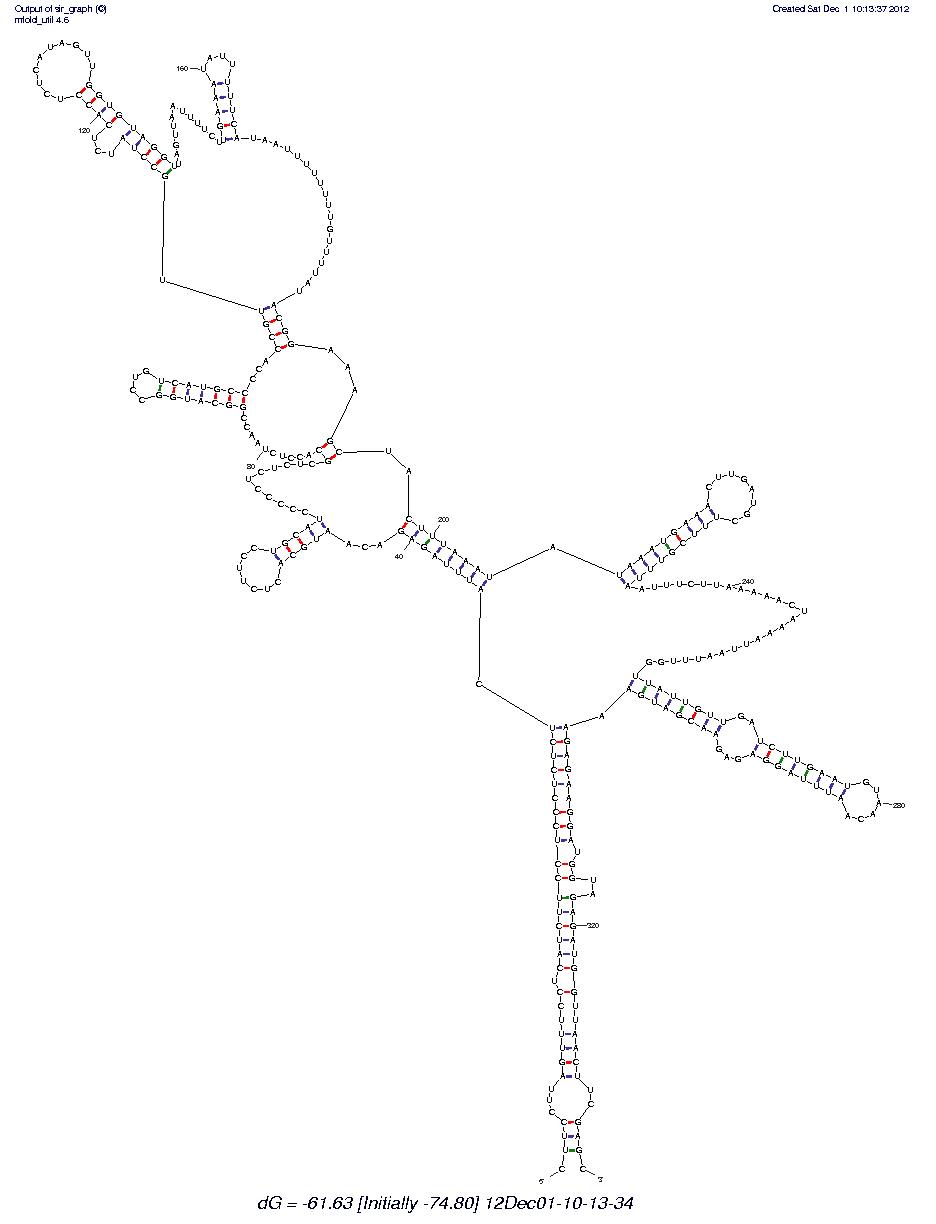


rcomiR008


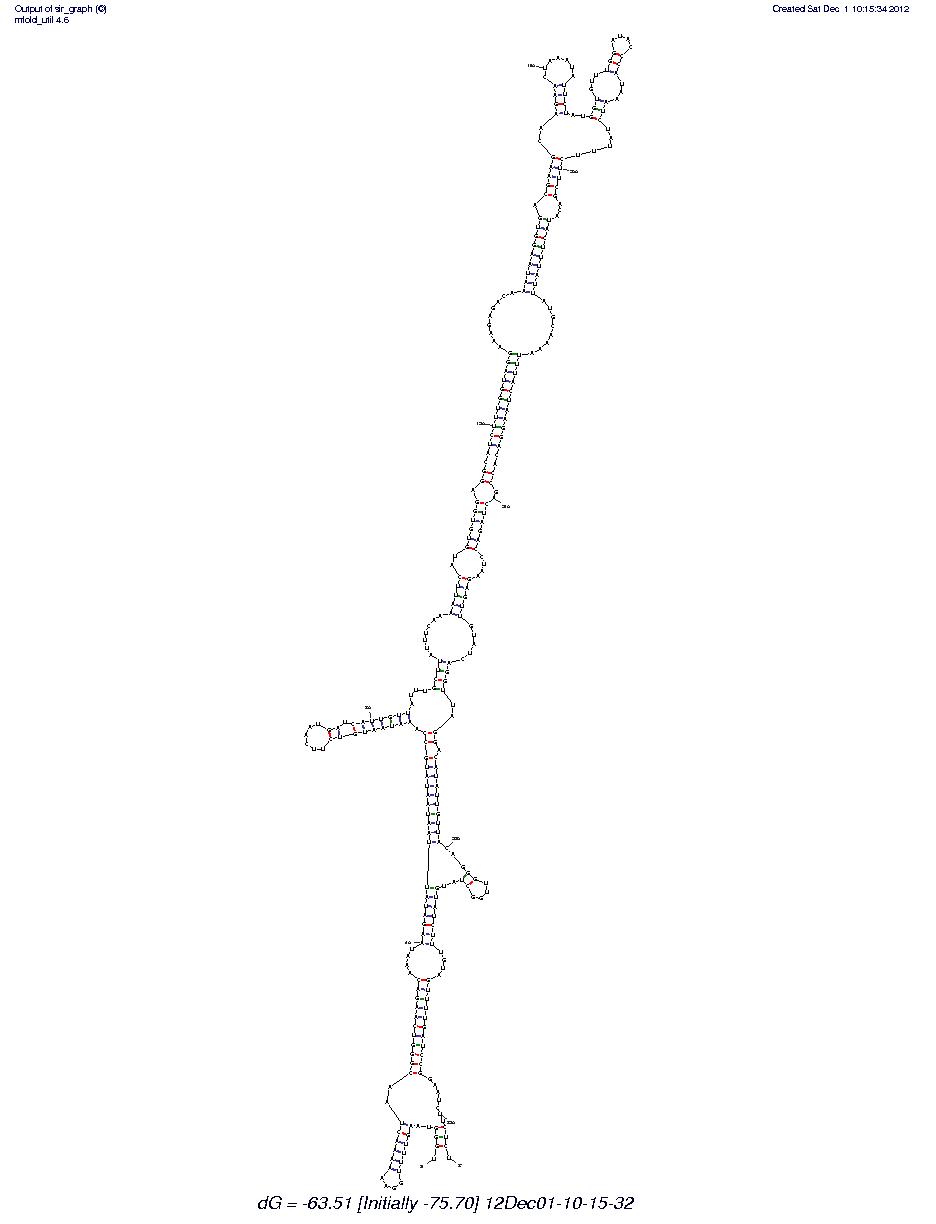


rcomiR009


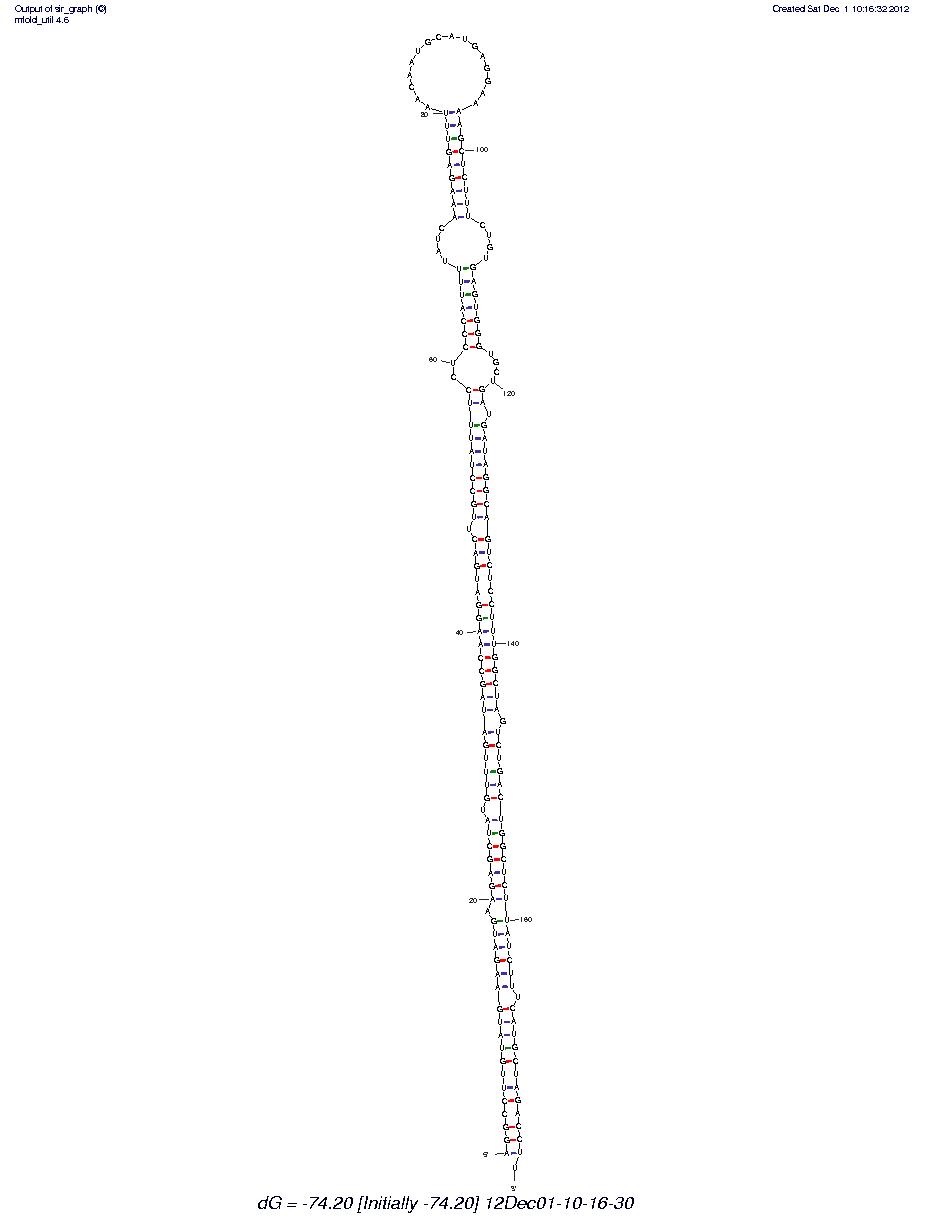


rcomiR010


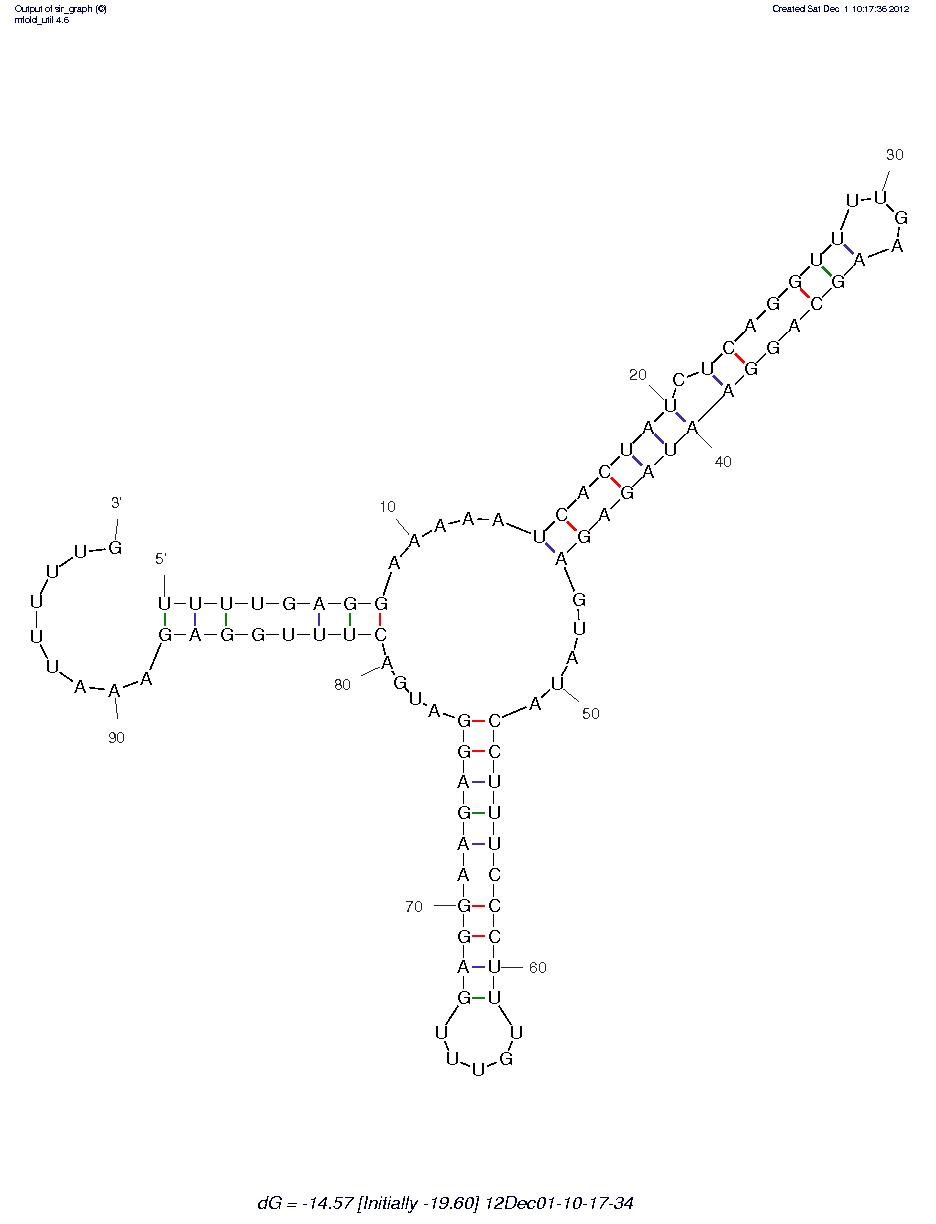


rcomiR011


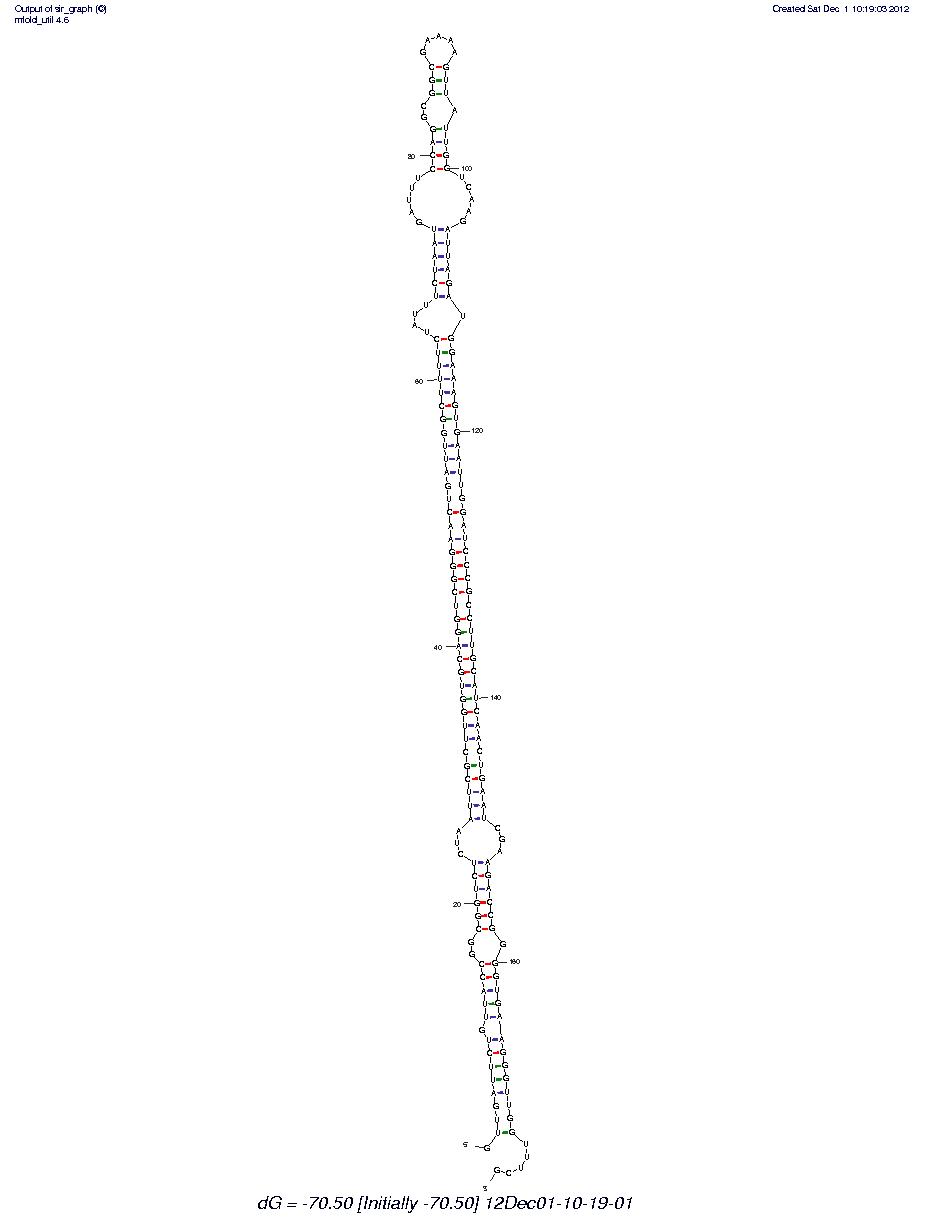


rcomiR012


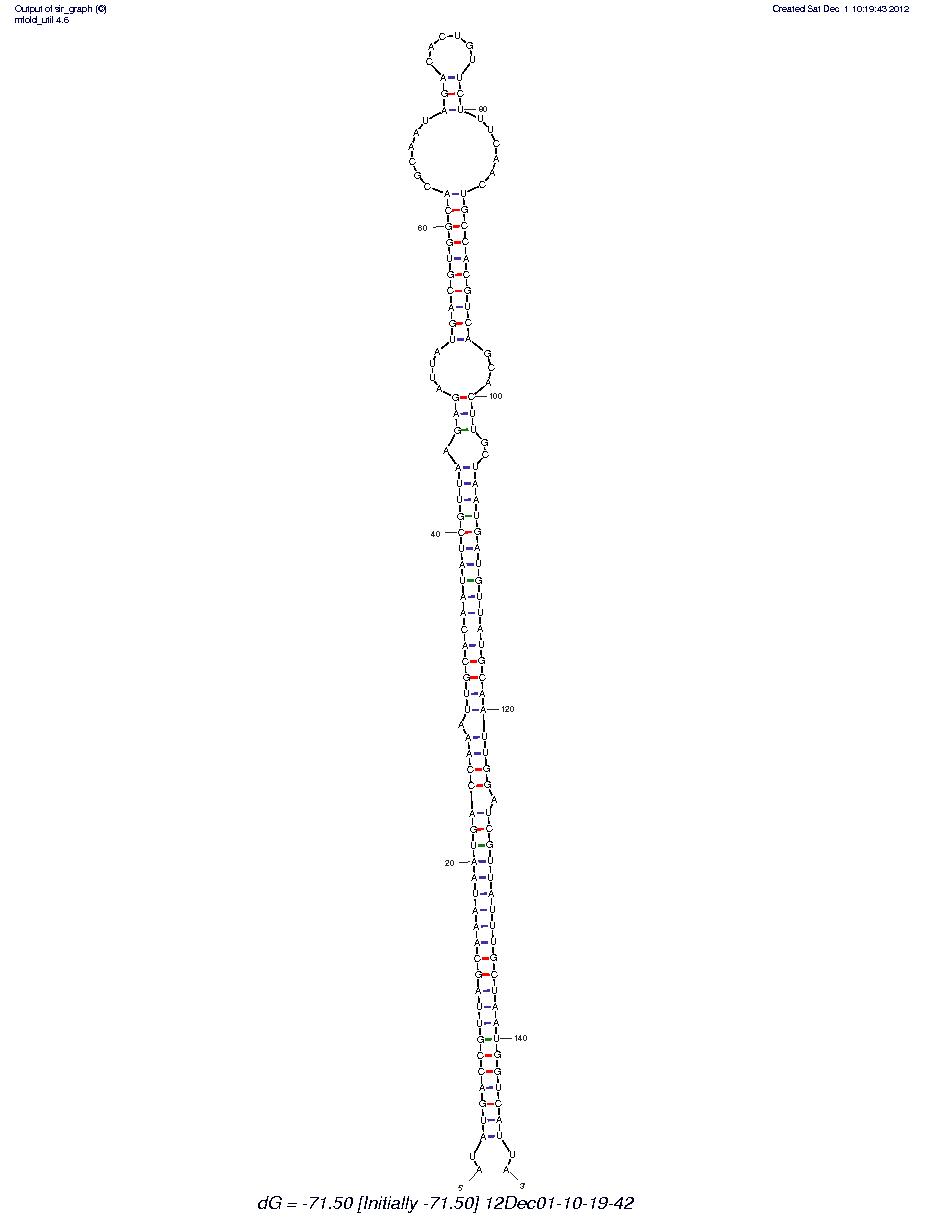


rcomiR013


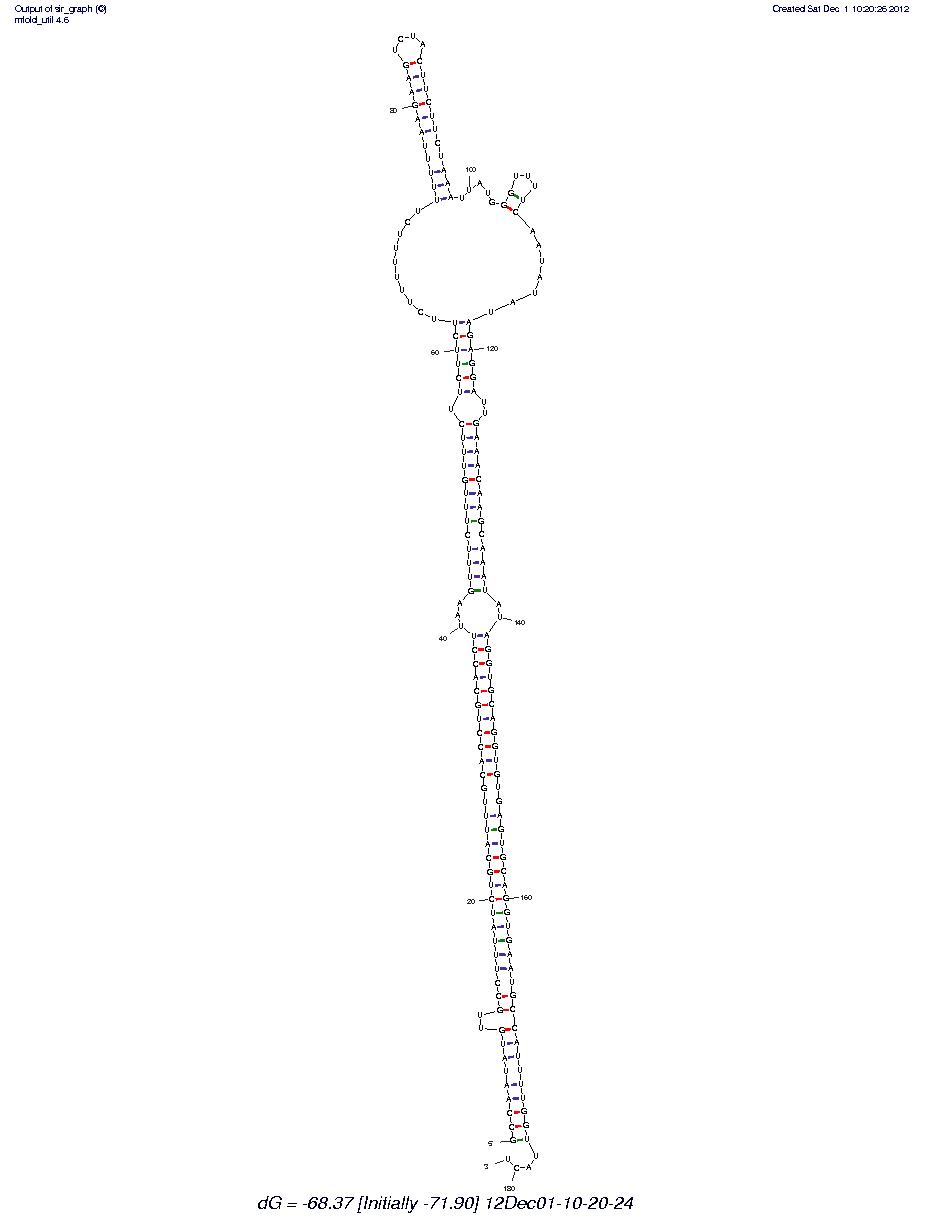


rcomiR014


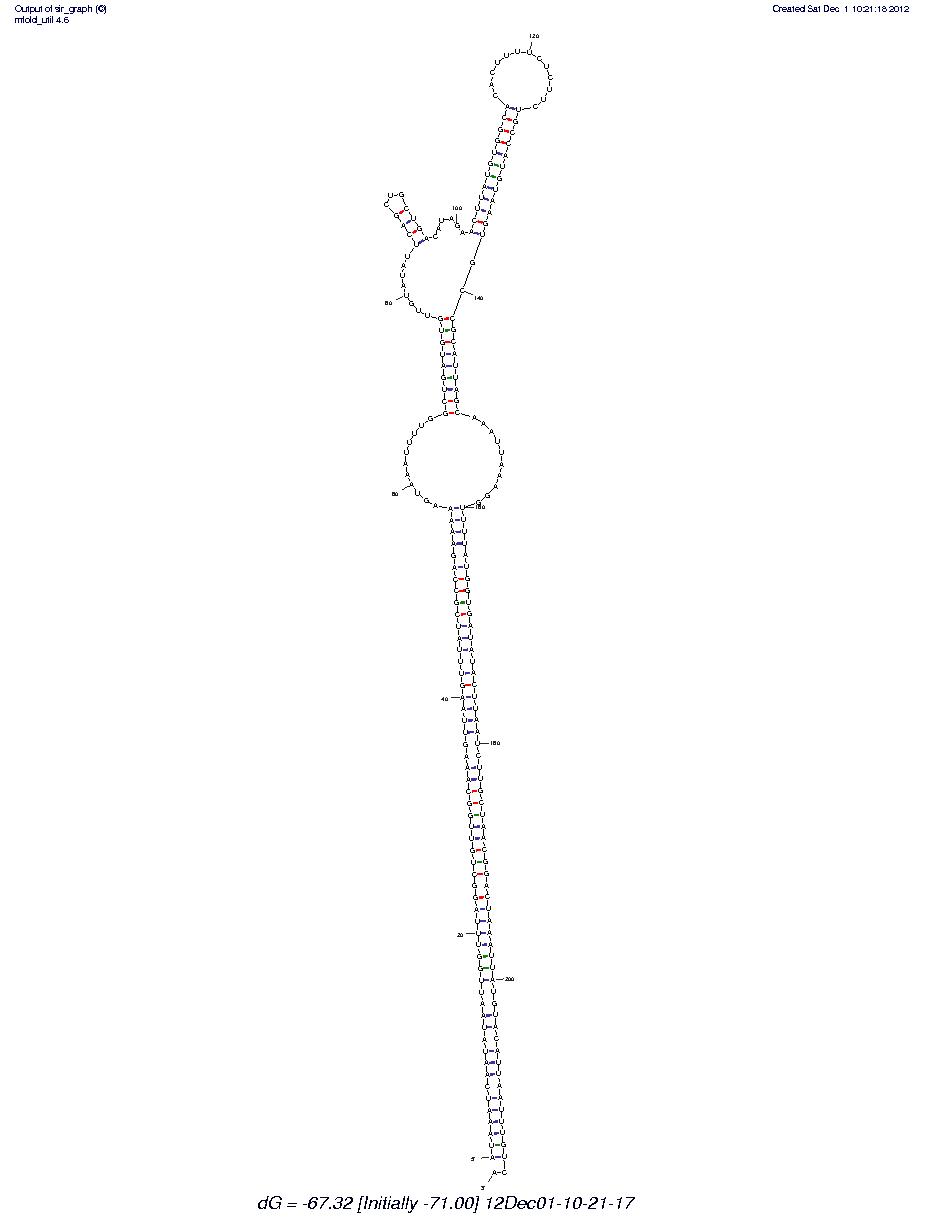


rcomiR015


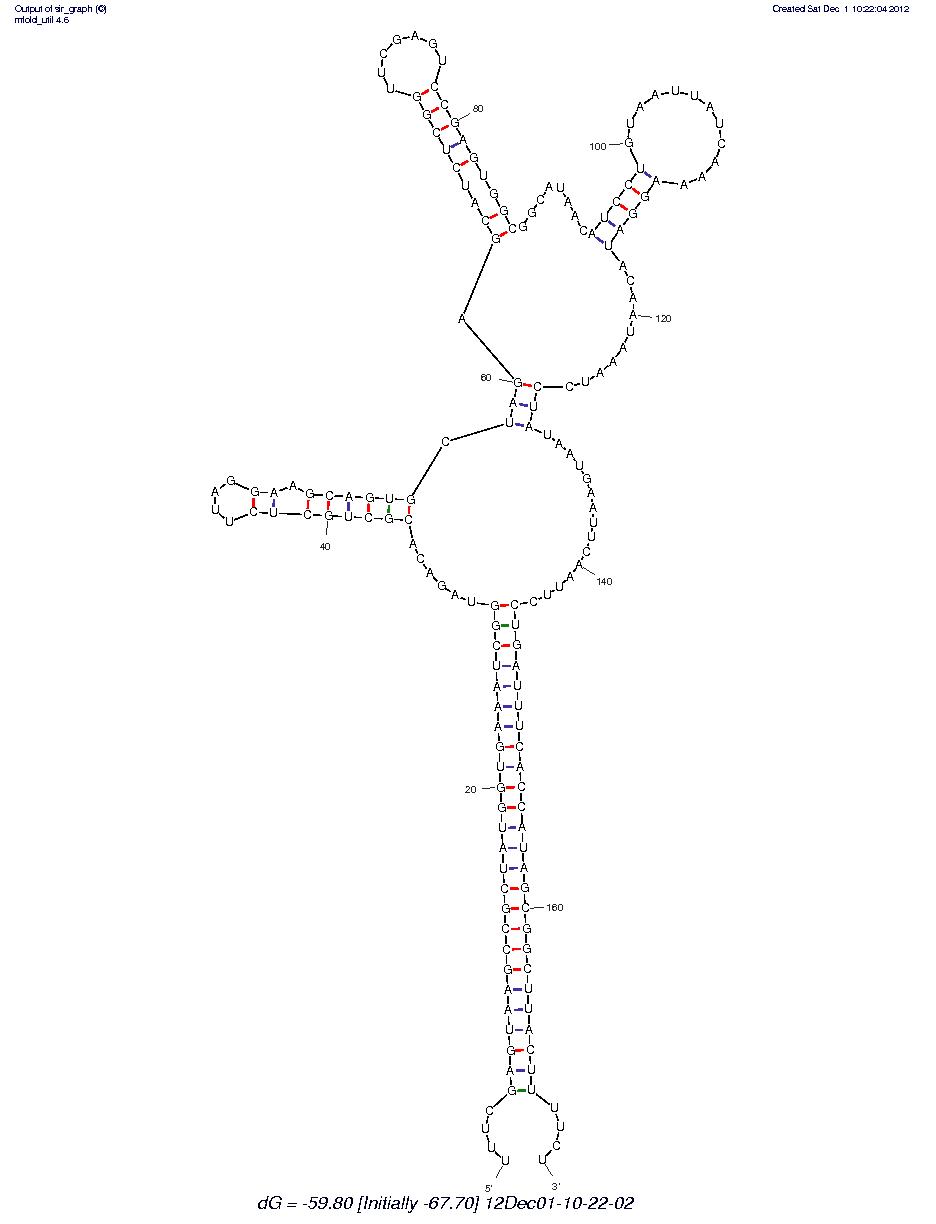


rcomiR016


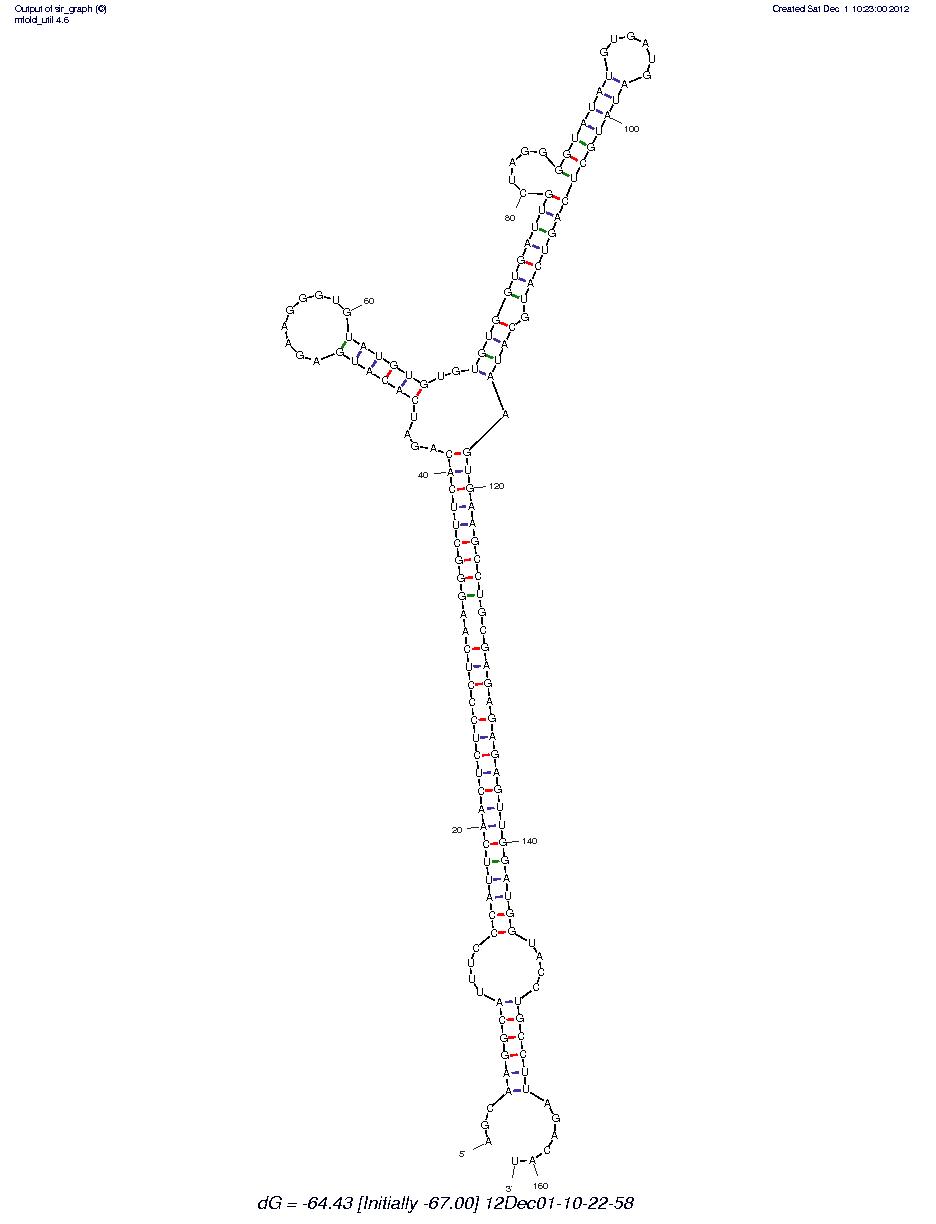


rcomiR017


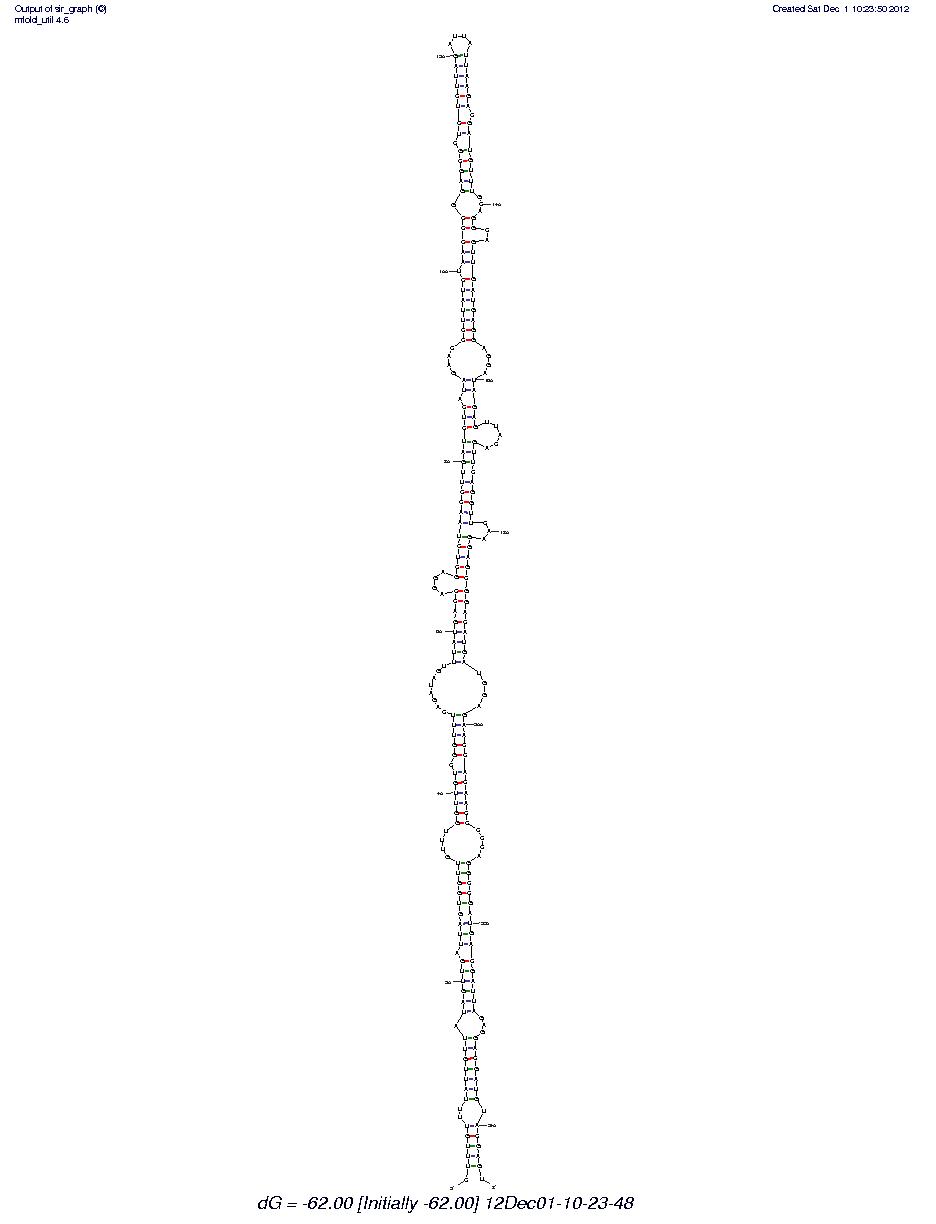


rcomiR018
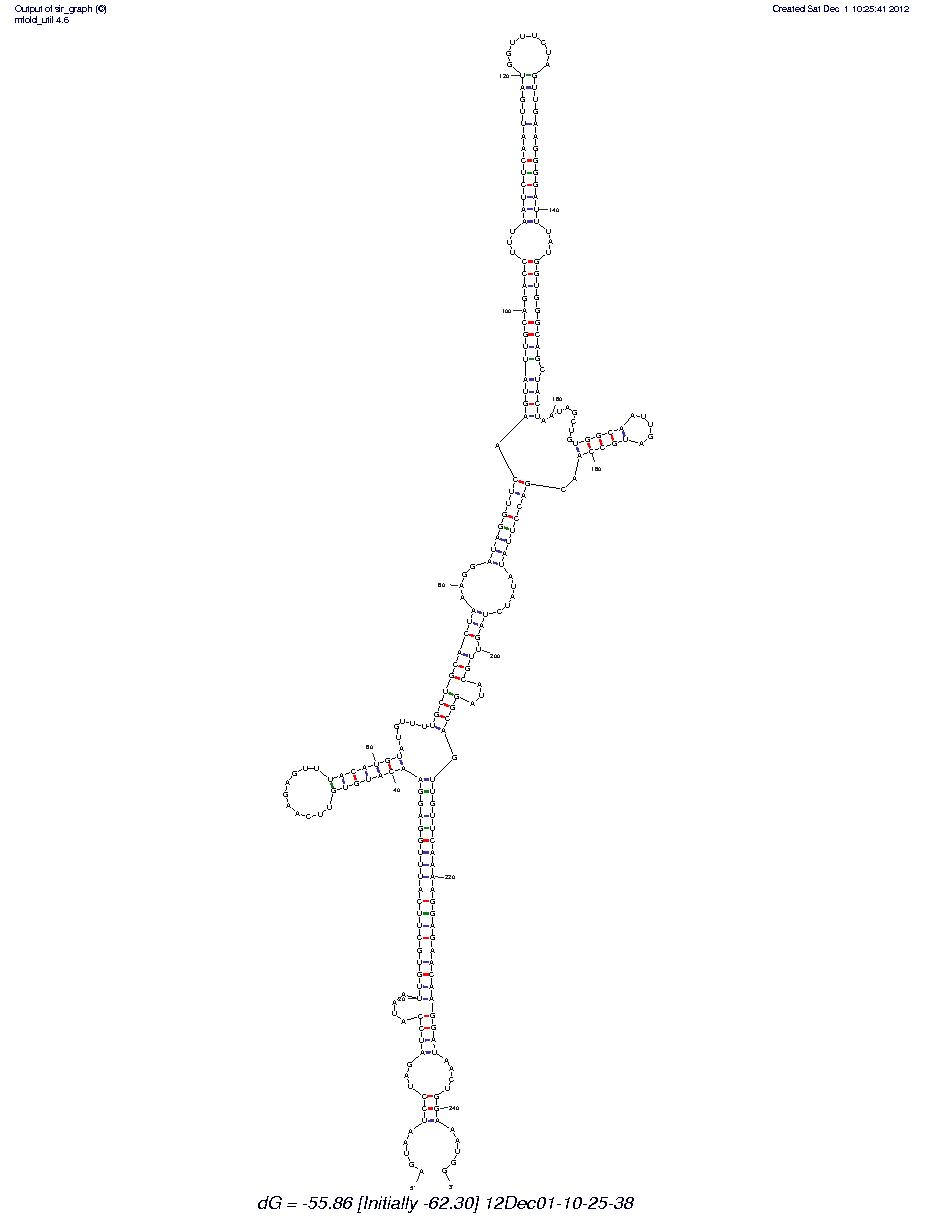


rcomiR019
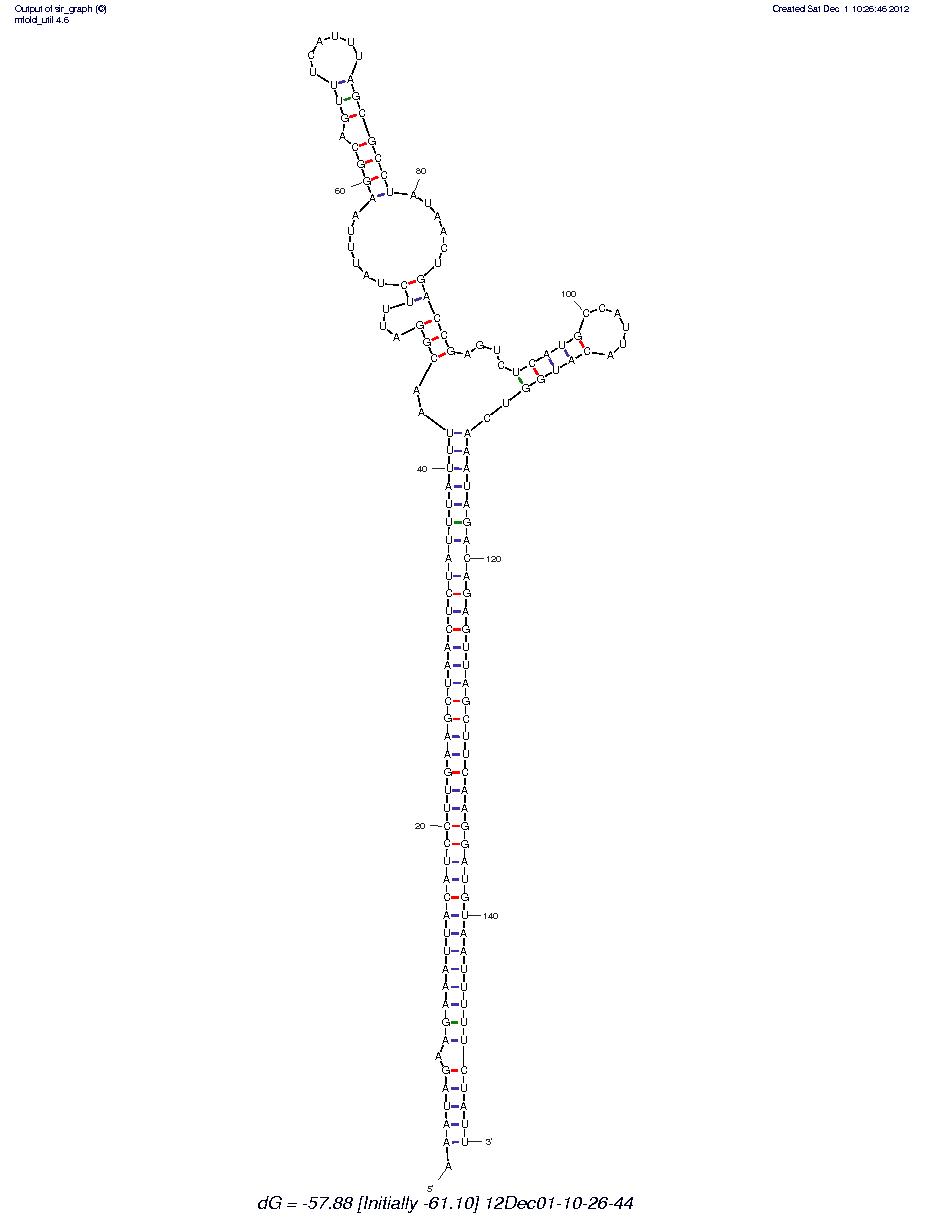


rcomiR020


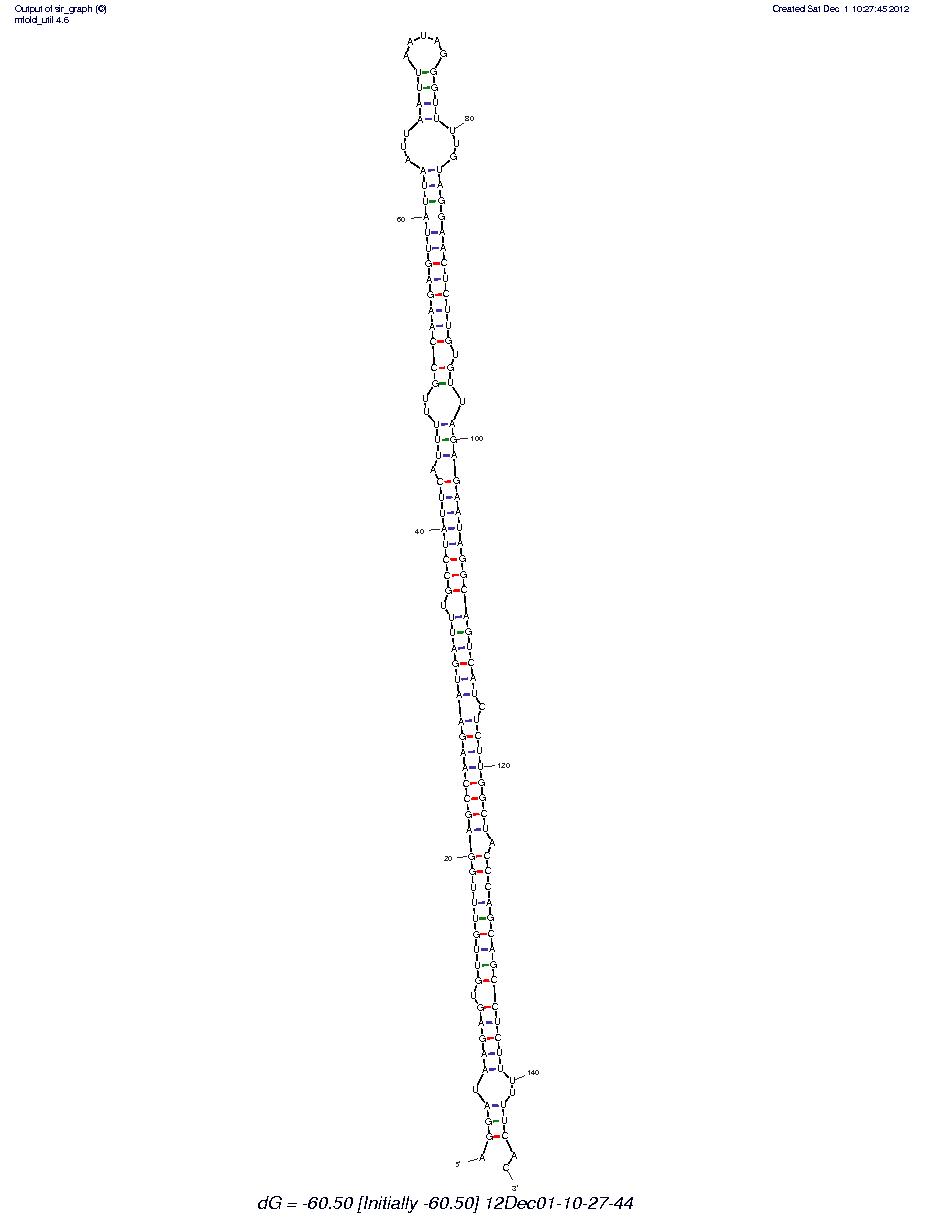


rcomiR021


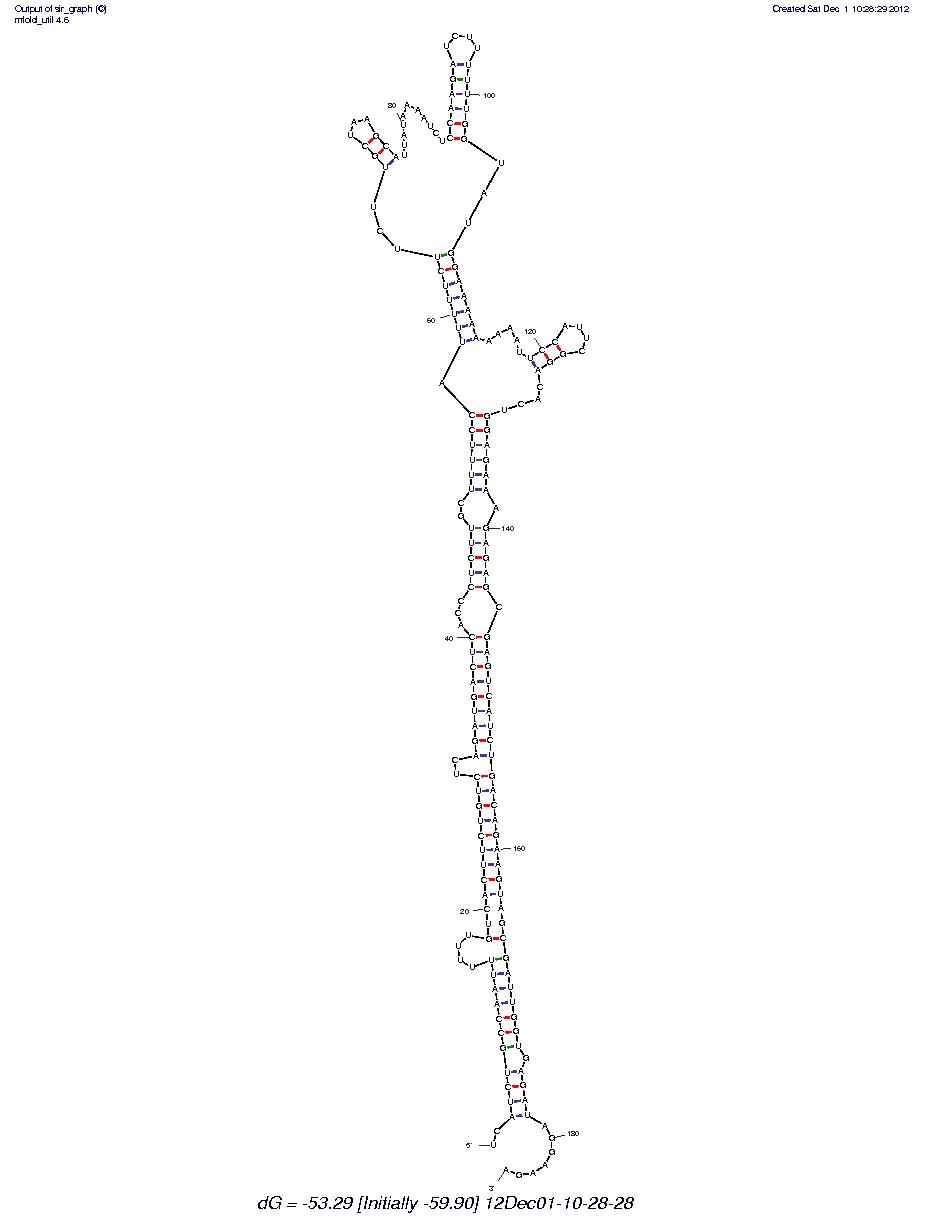


rcomiR022
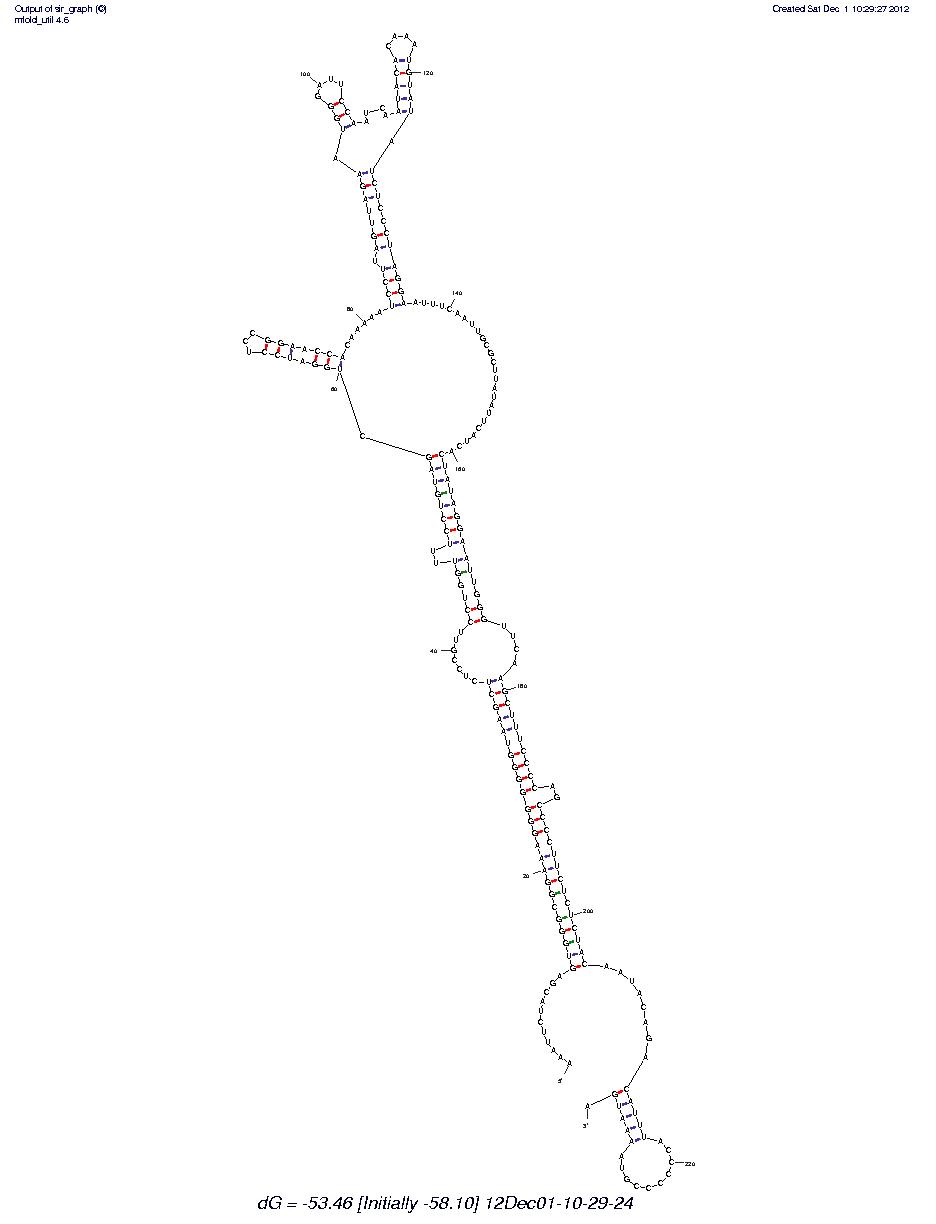


rcomiR023


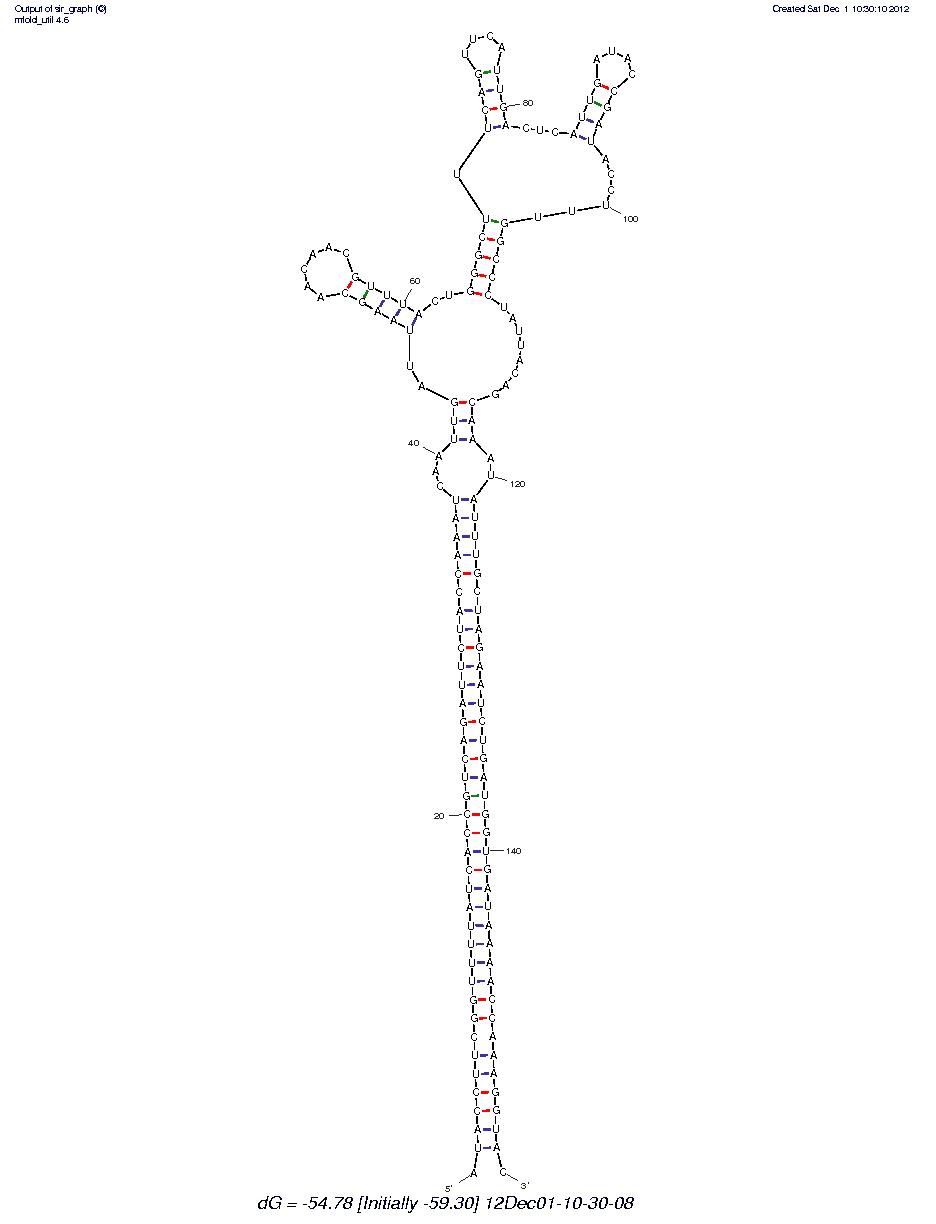


rcomiR024


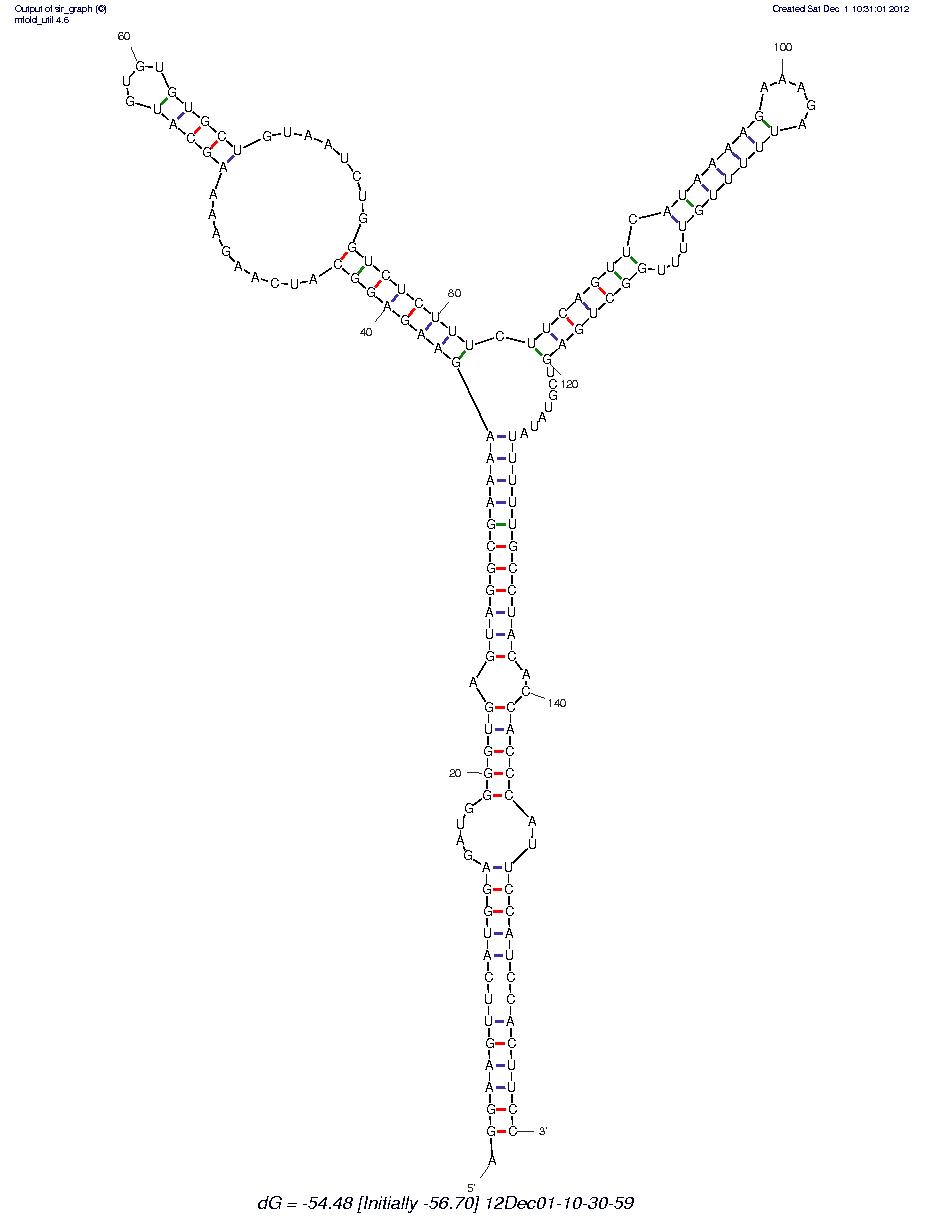


rcomiR025


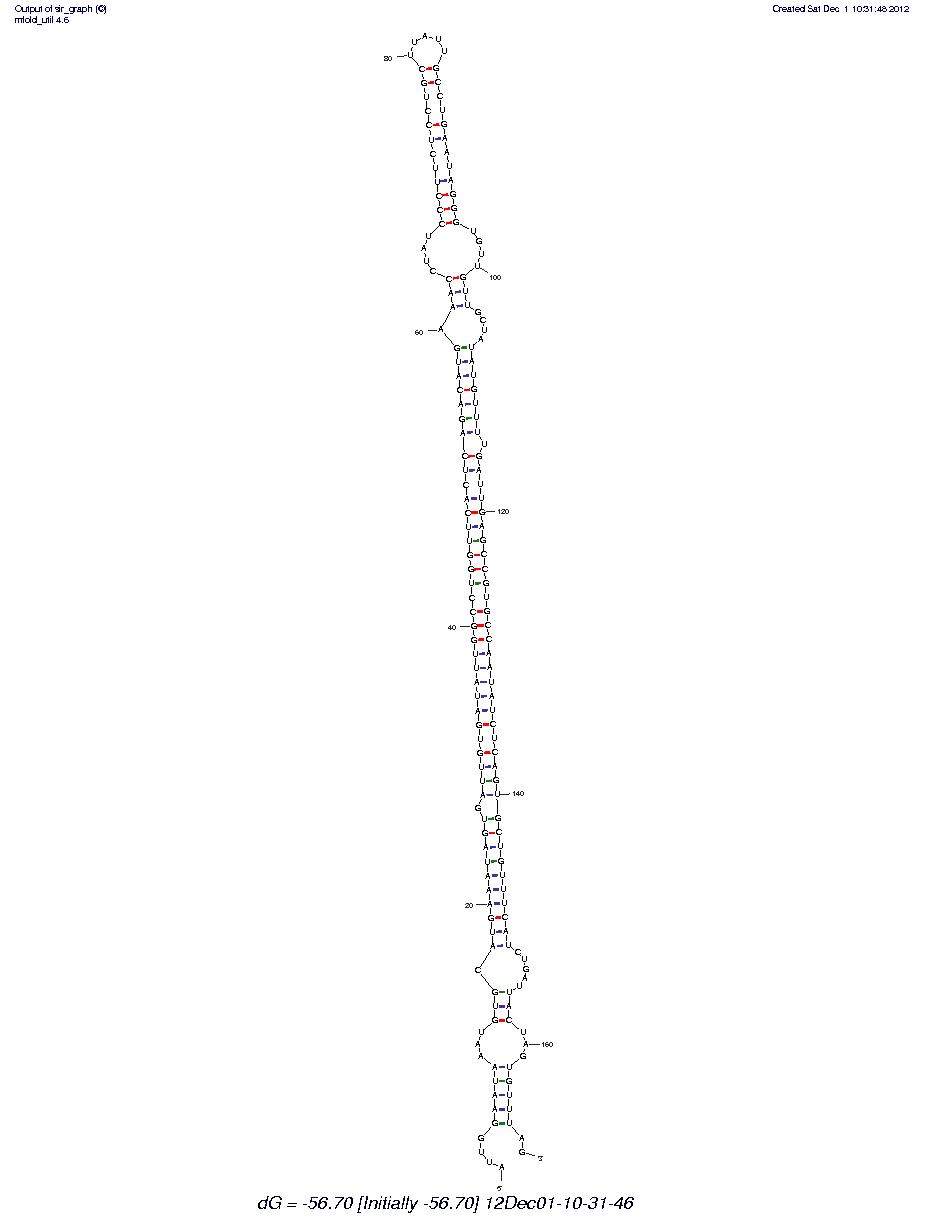


rcomiR026


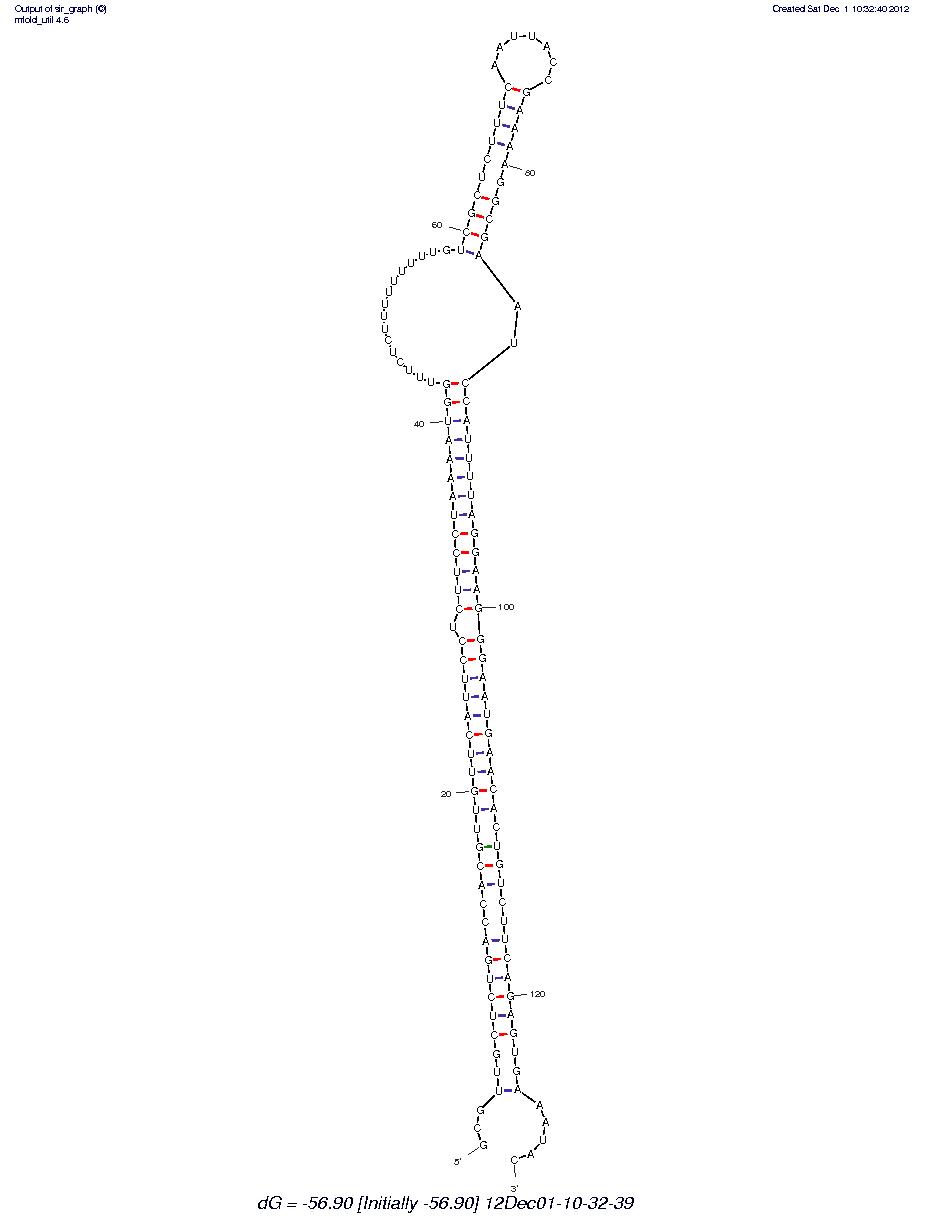


rcomiR027


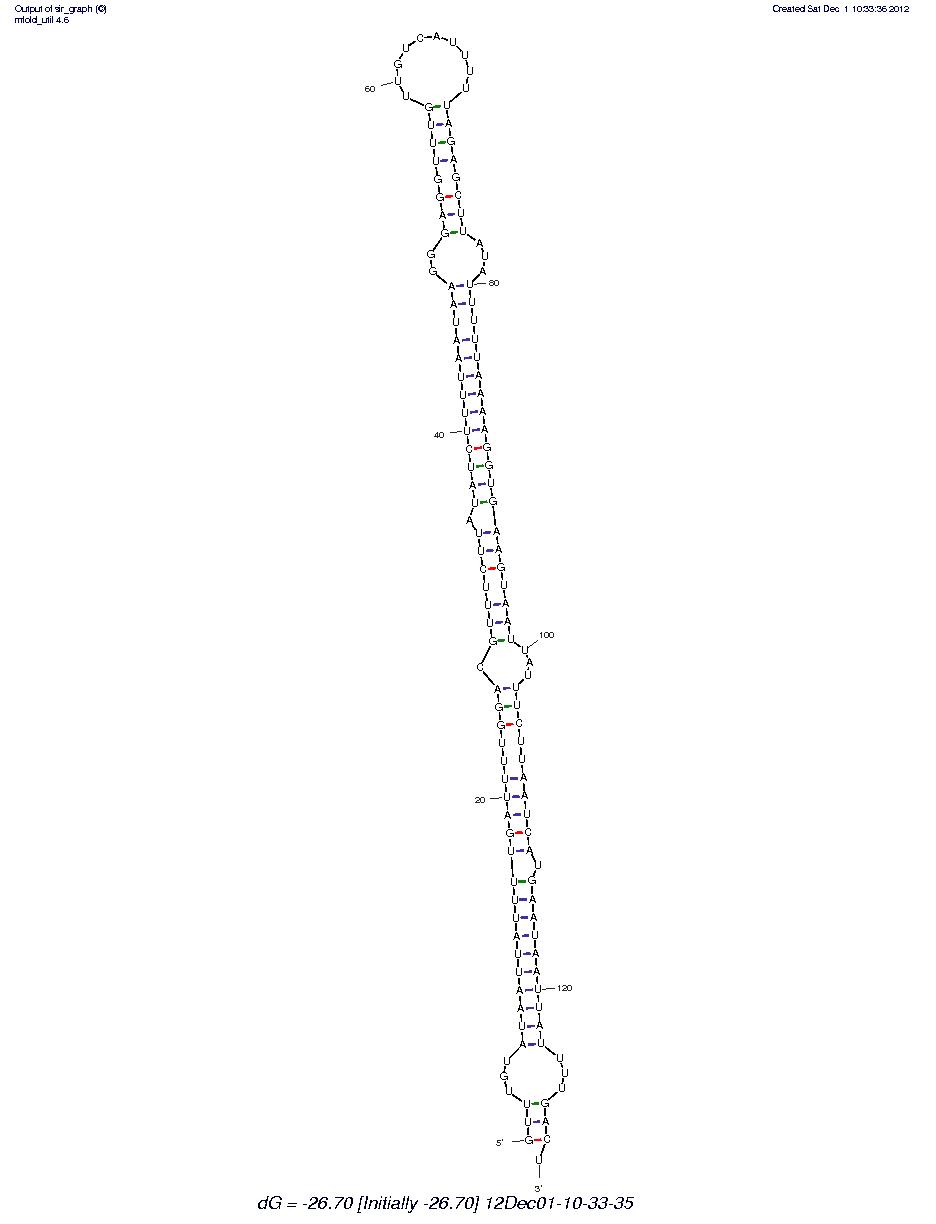


rcomiR028
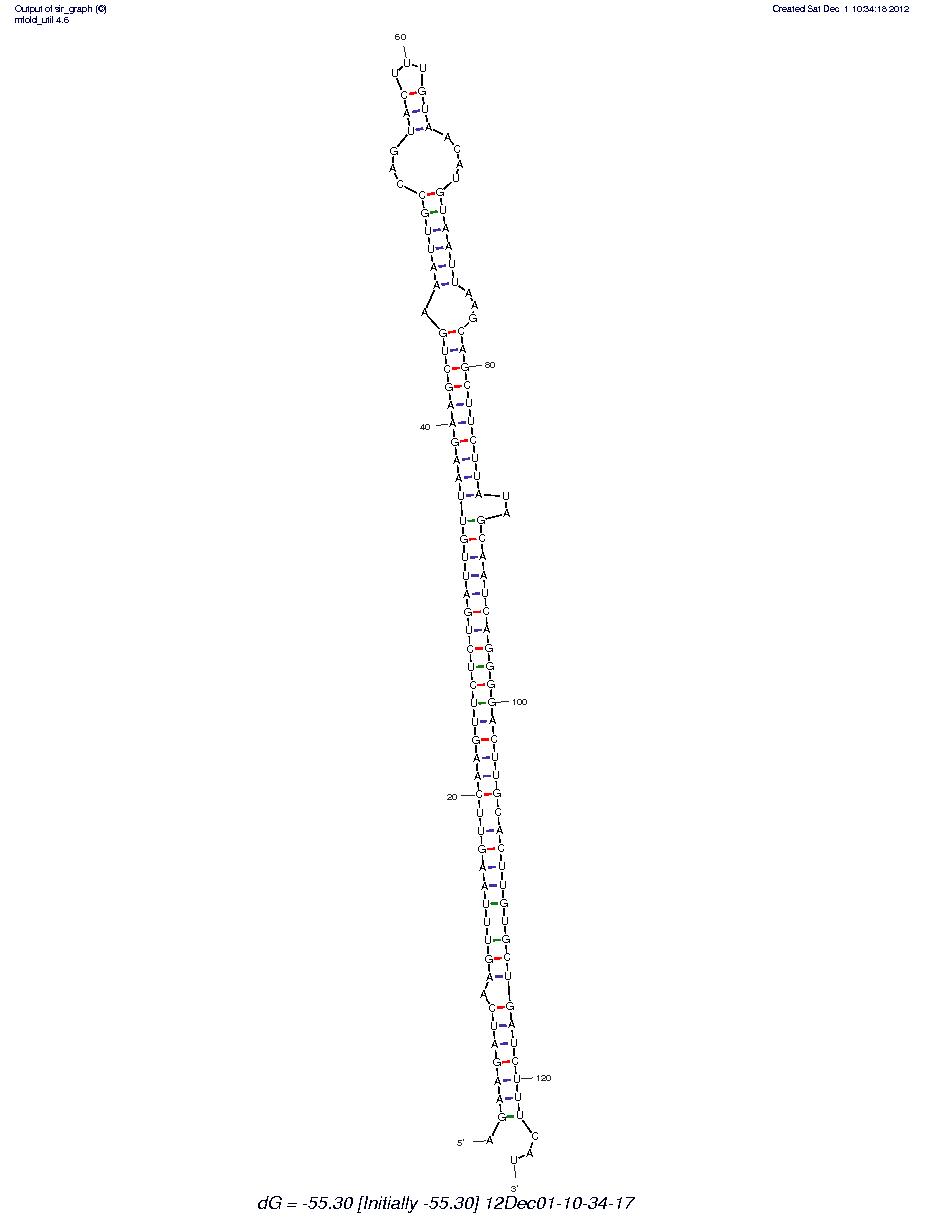


rcomiR029


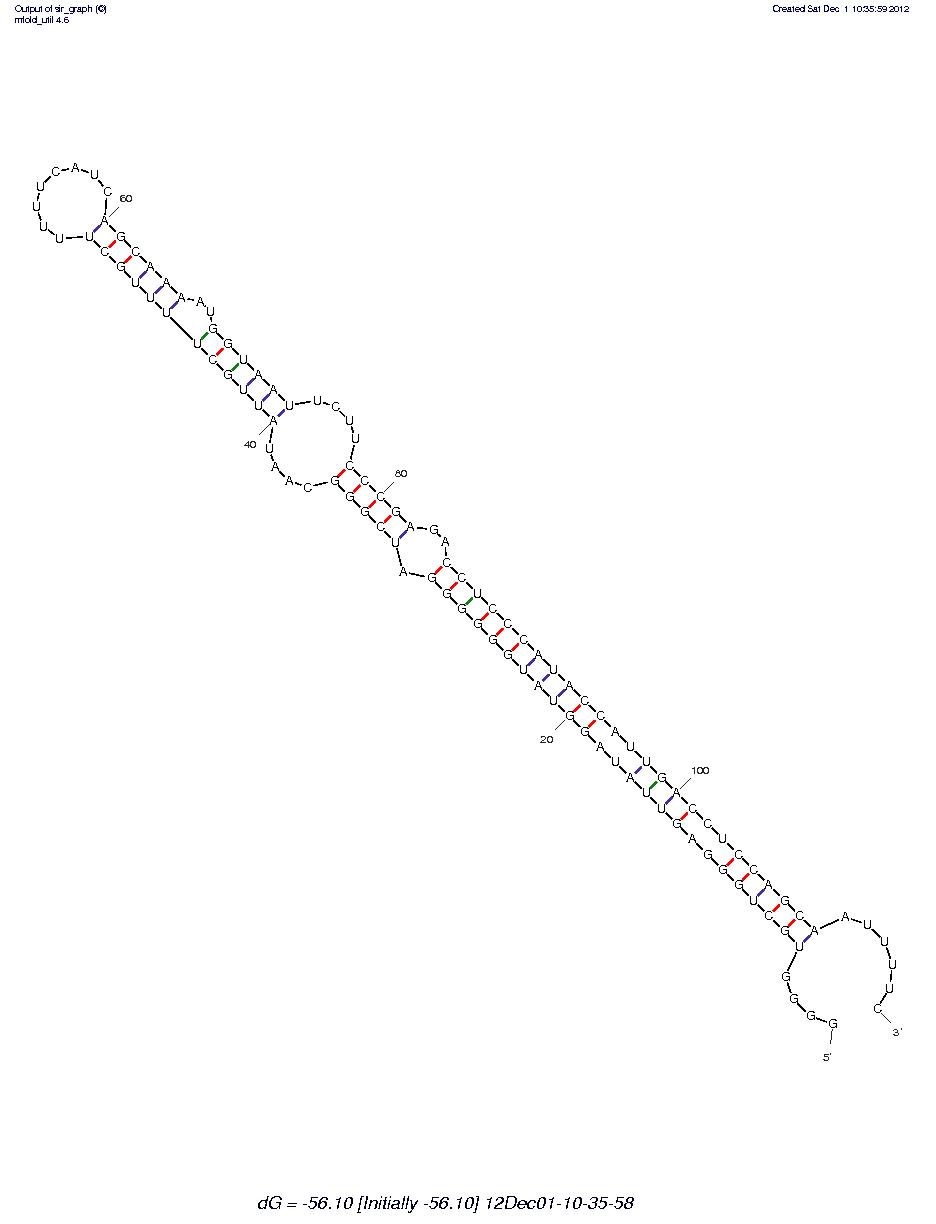


rcomiR030


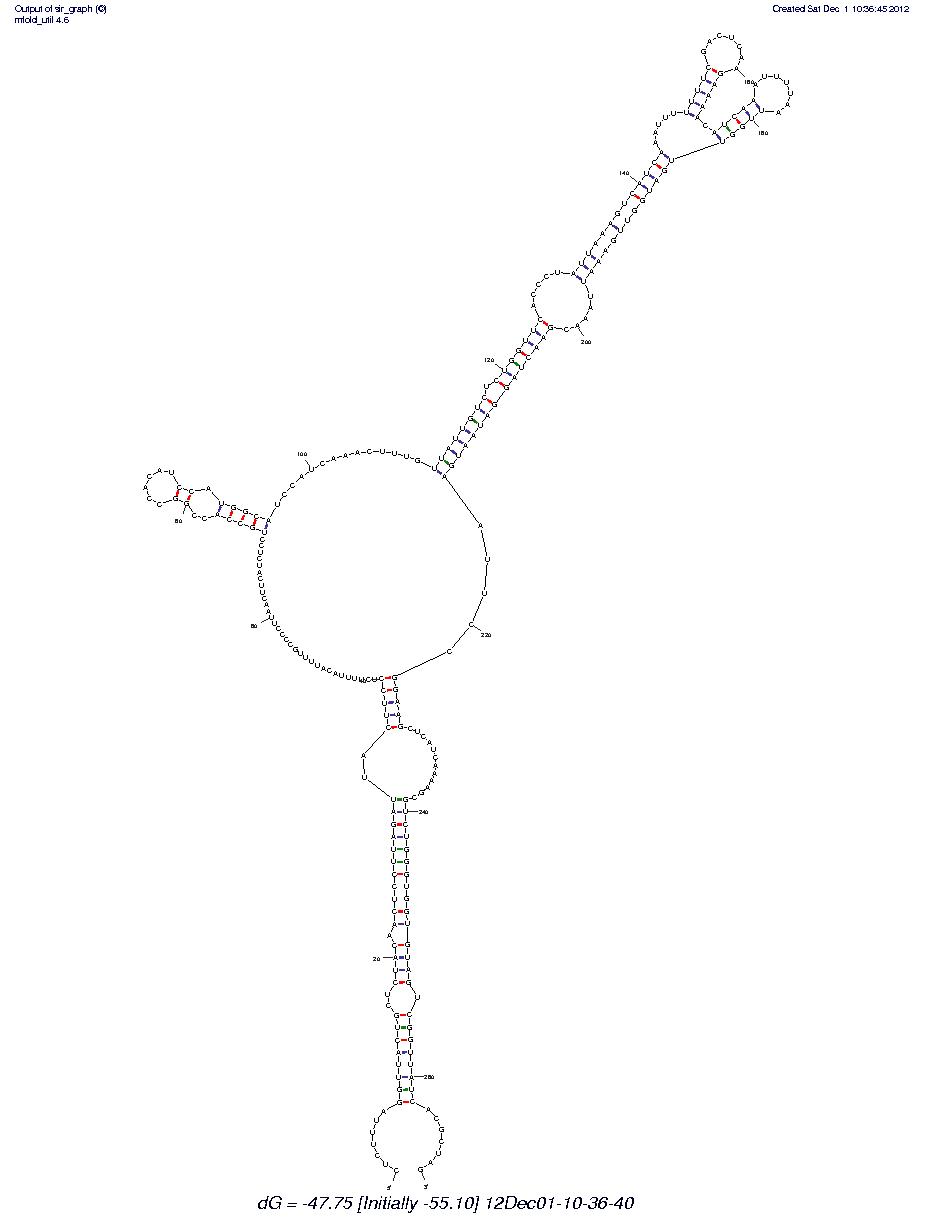


rcomiR031


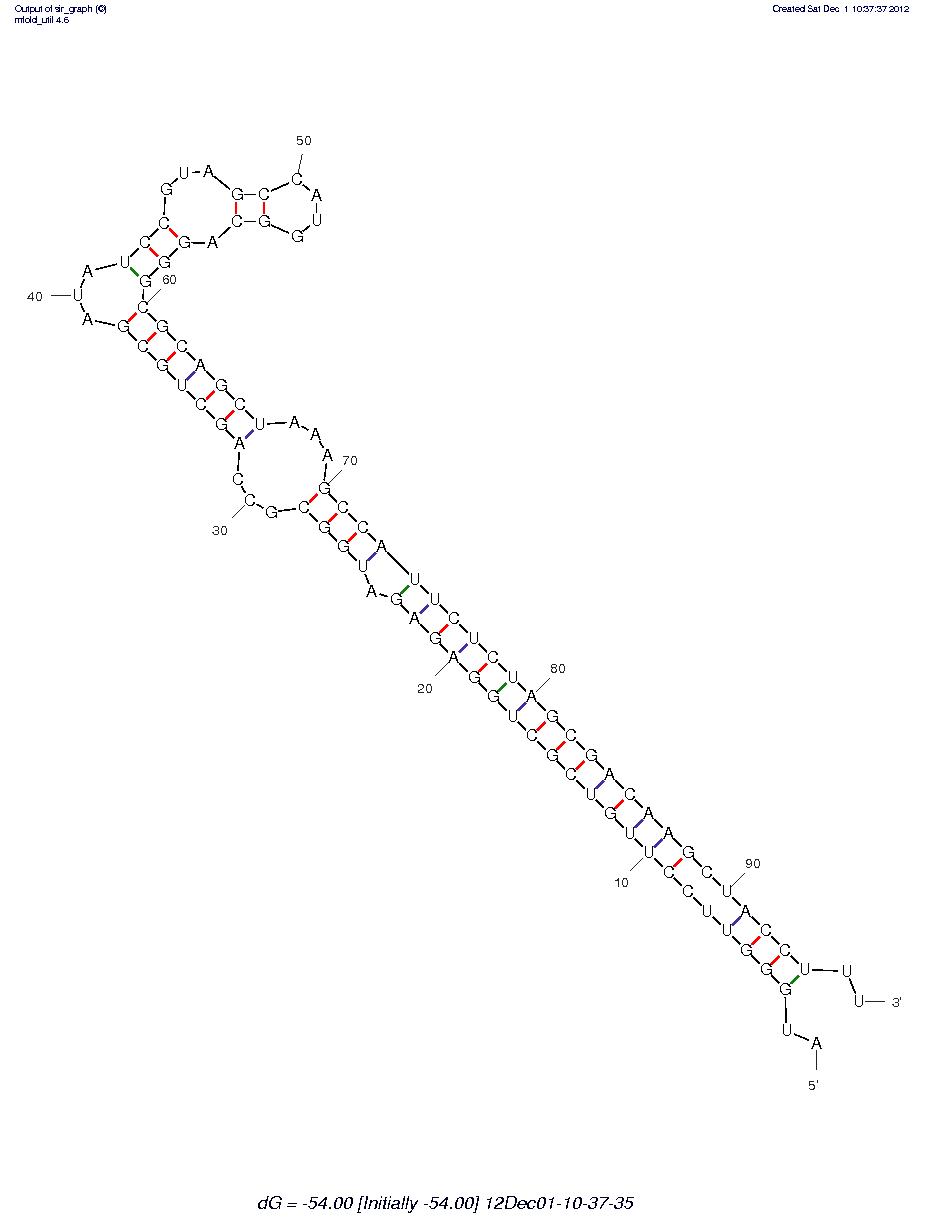


rcomiR032


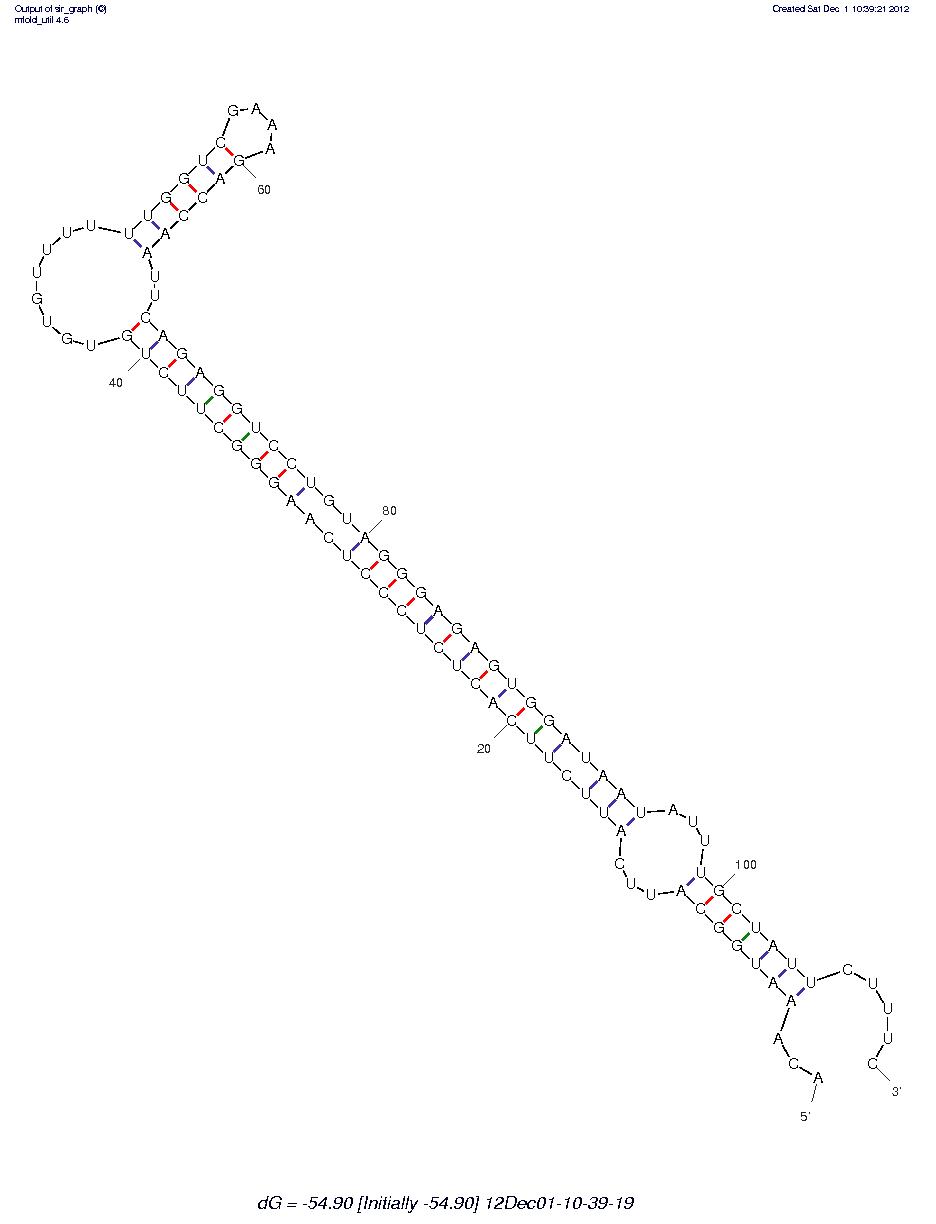


rcomiR033


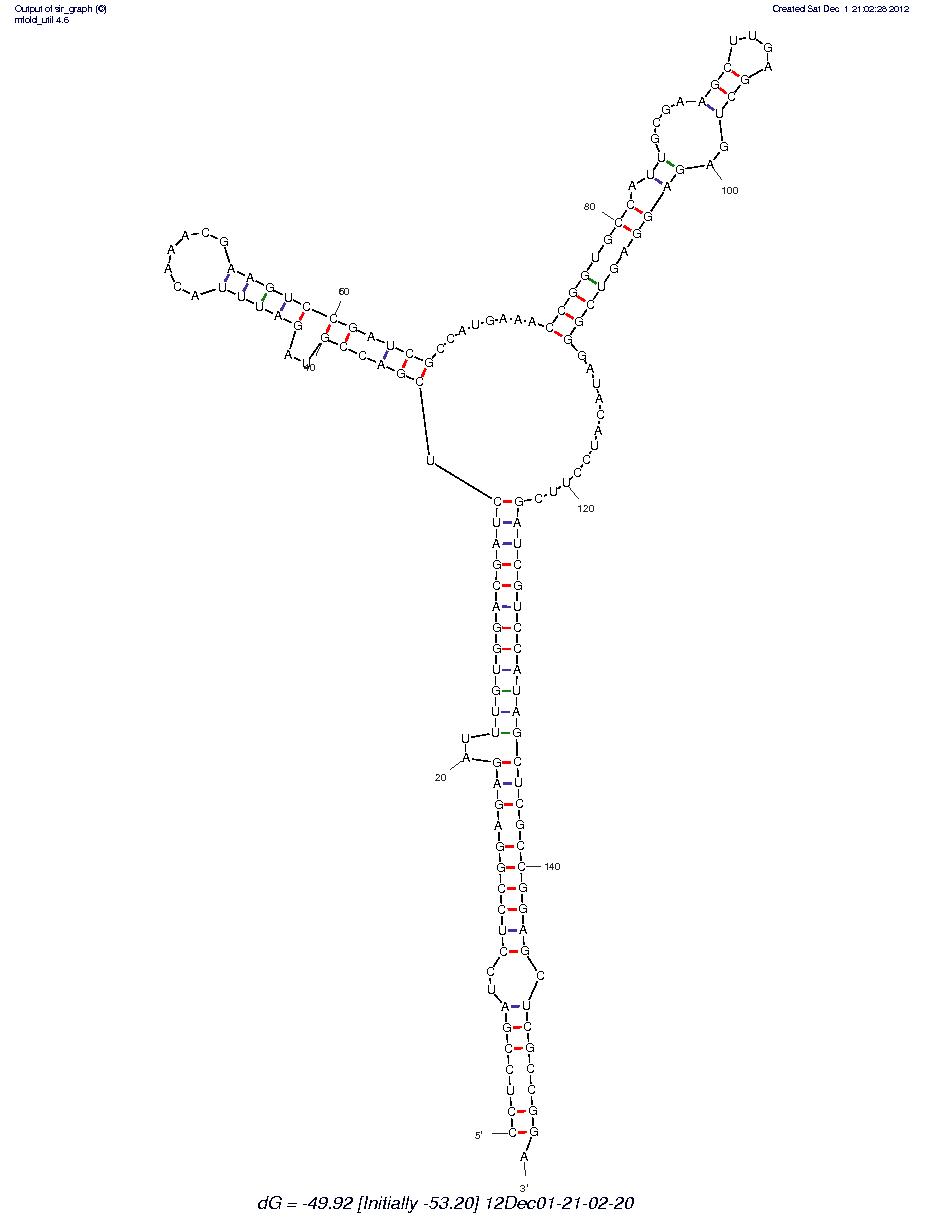


rcomiR034


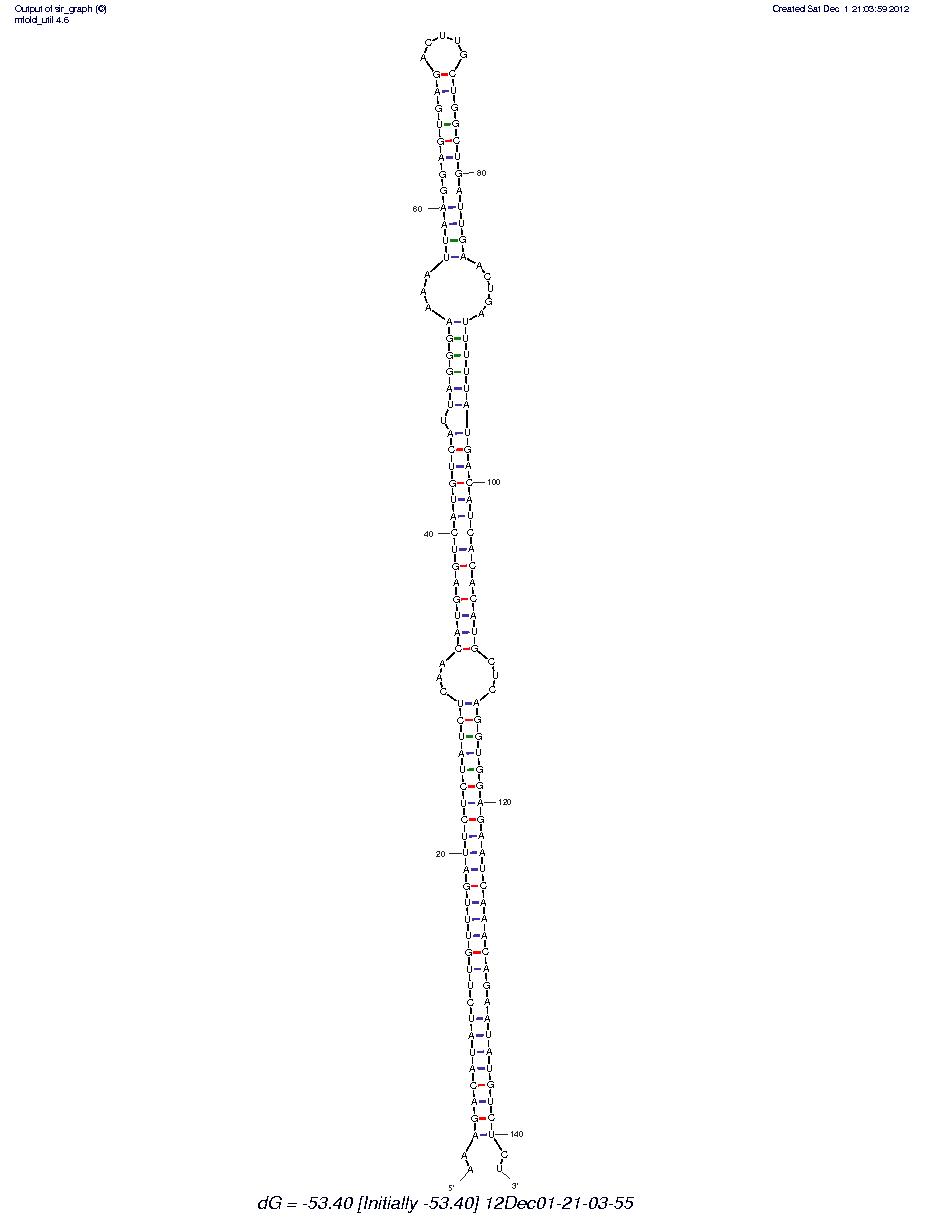


rcomiR035


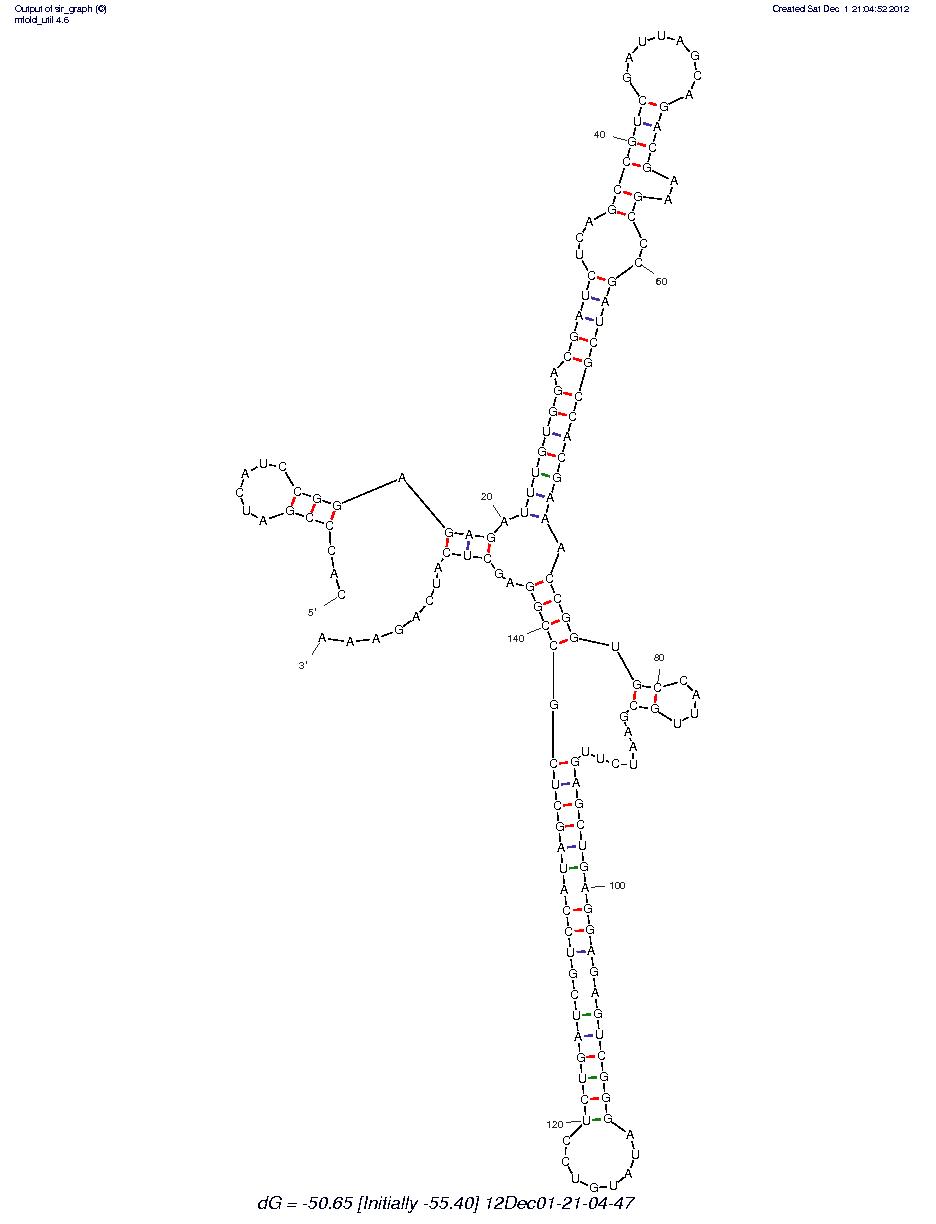


rcomiR036


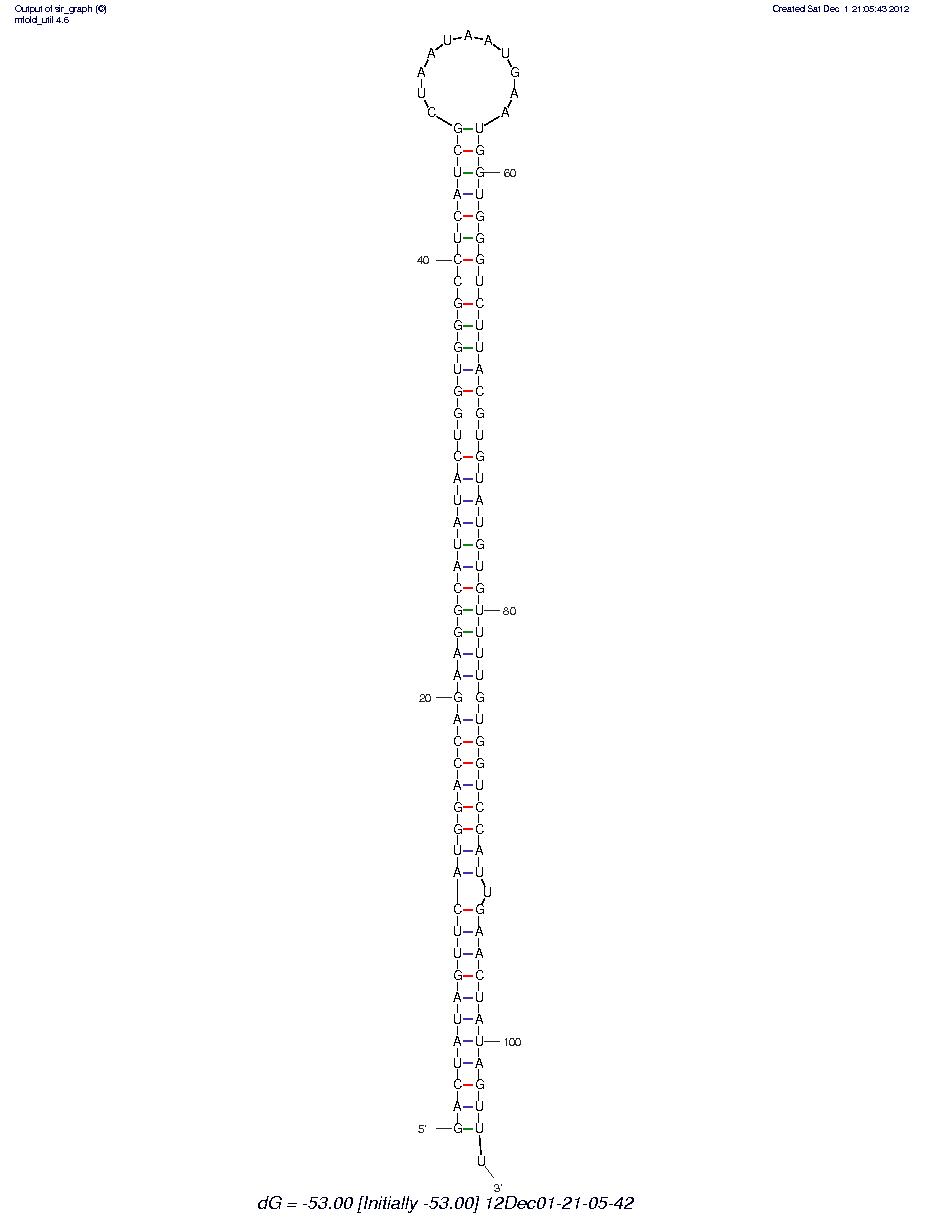


rcomiR037


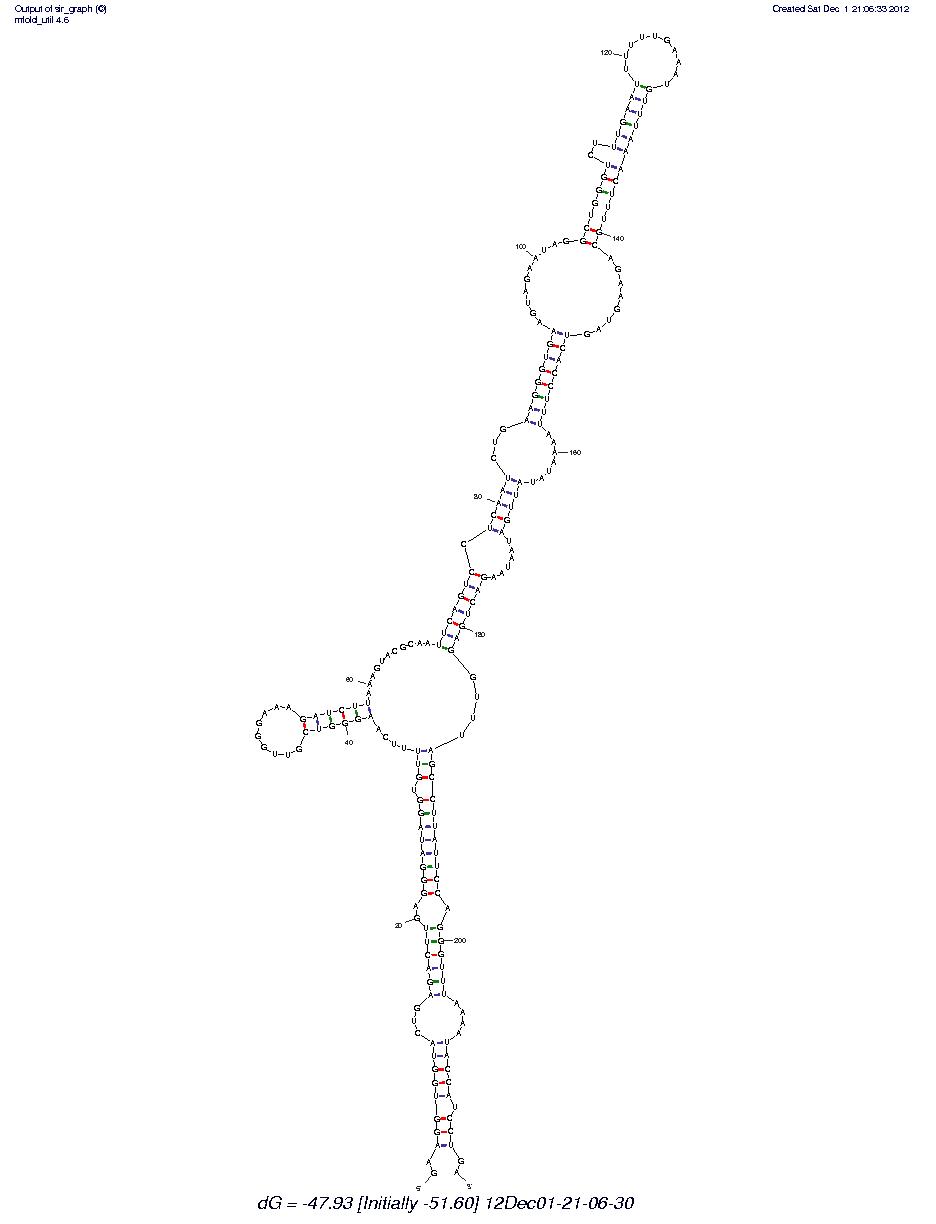


rcomiR038


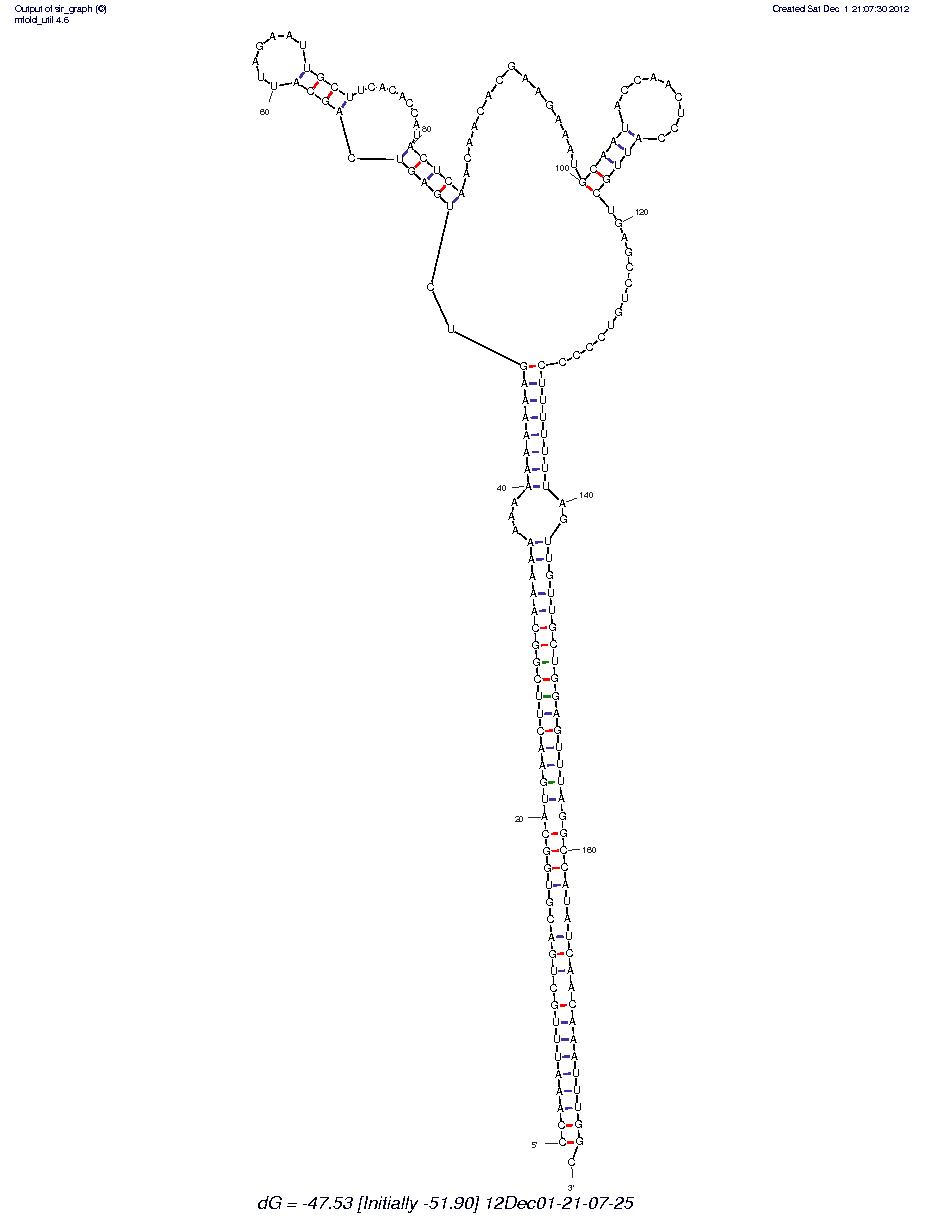


rcomiR039


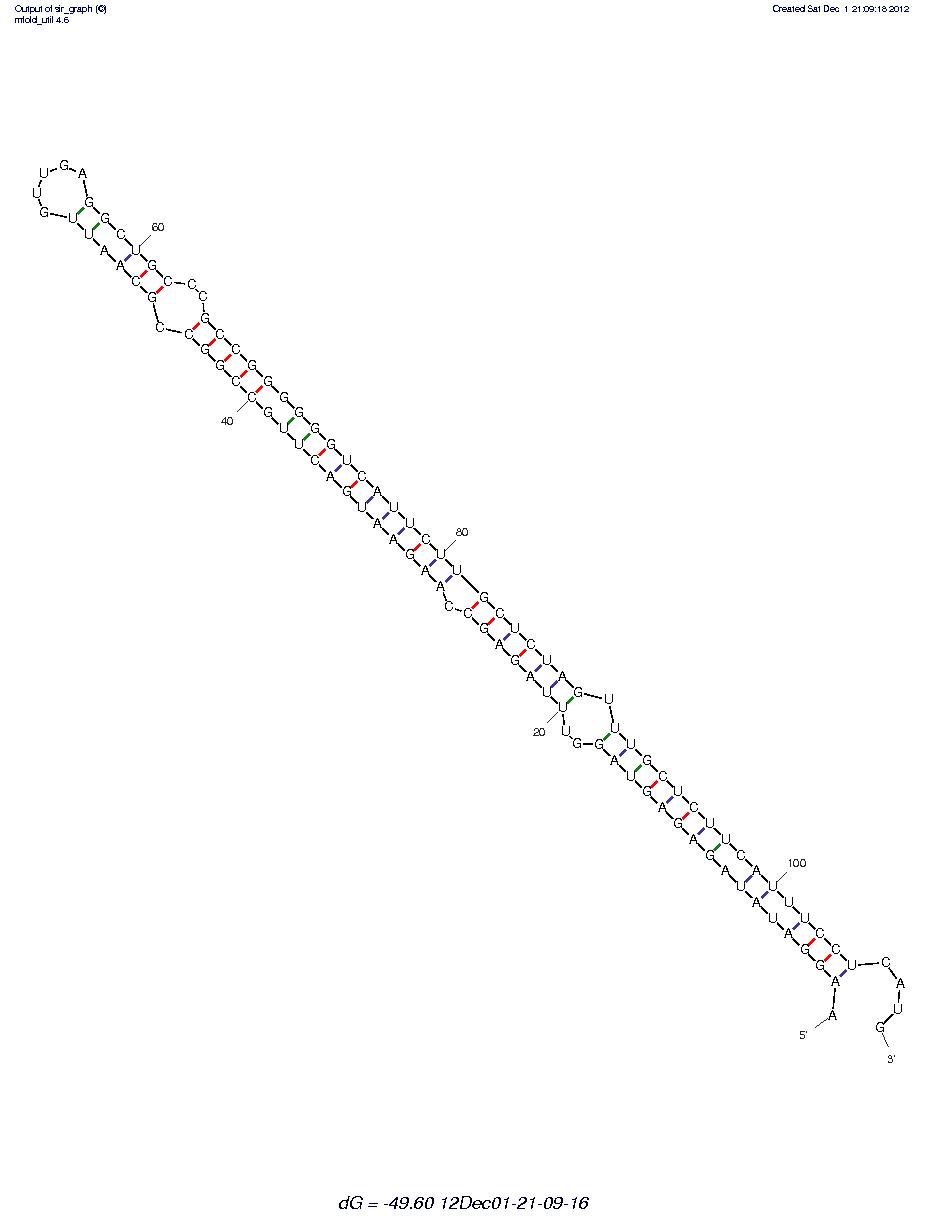


rcomiR040


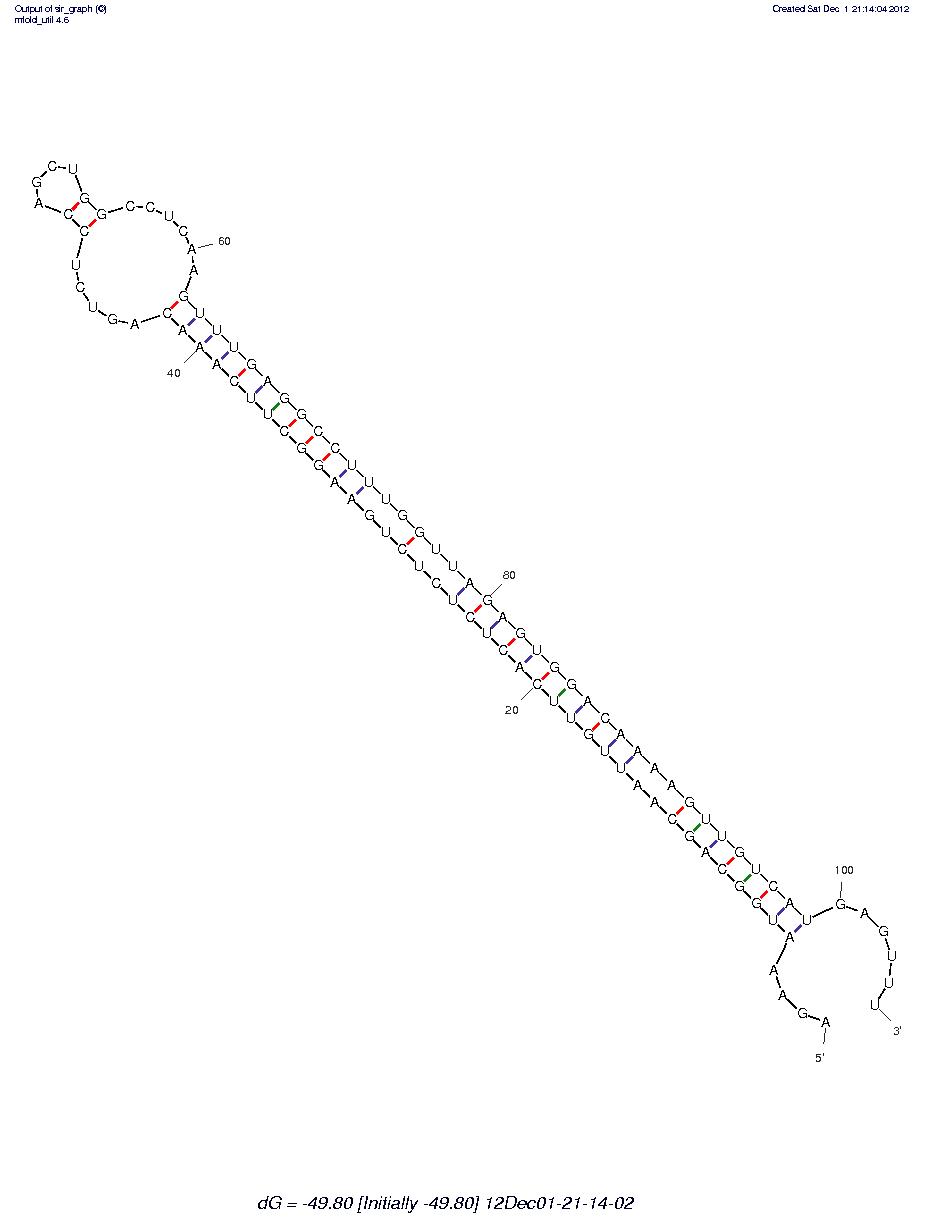


rcomiR041


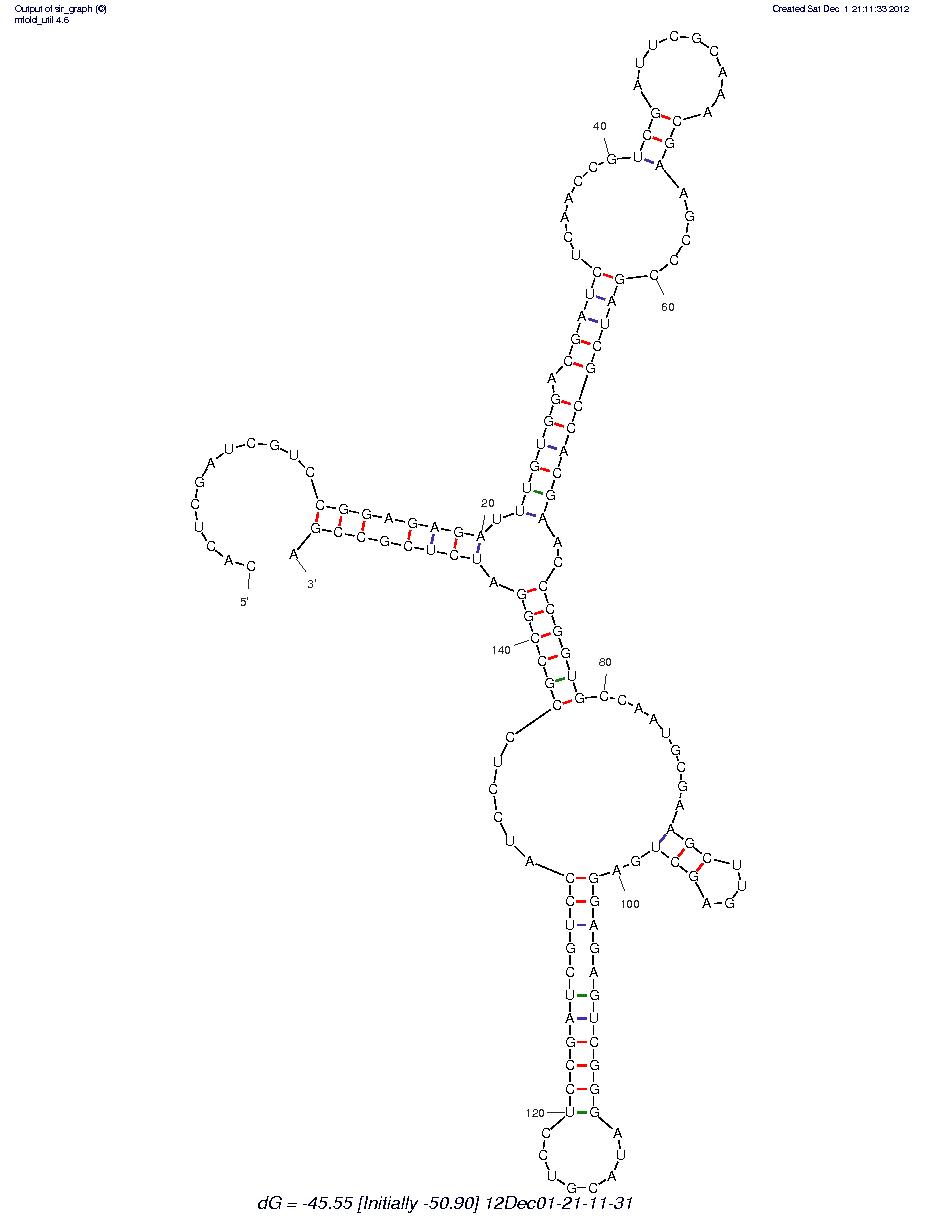


rcomiR042


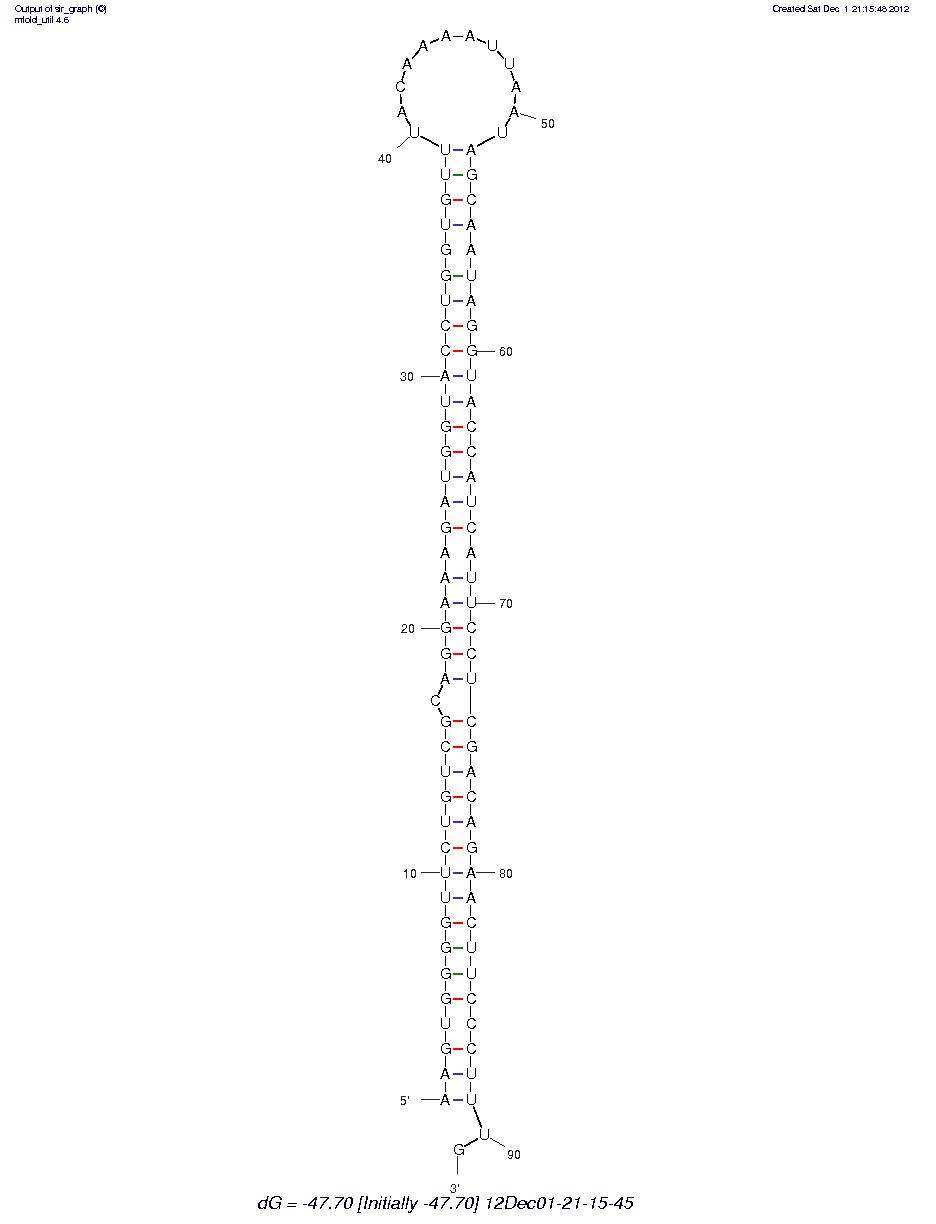


rcomiR043


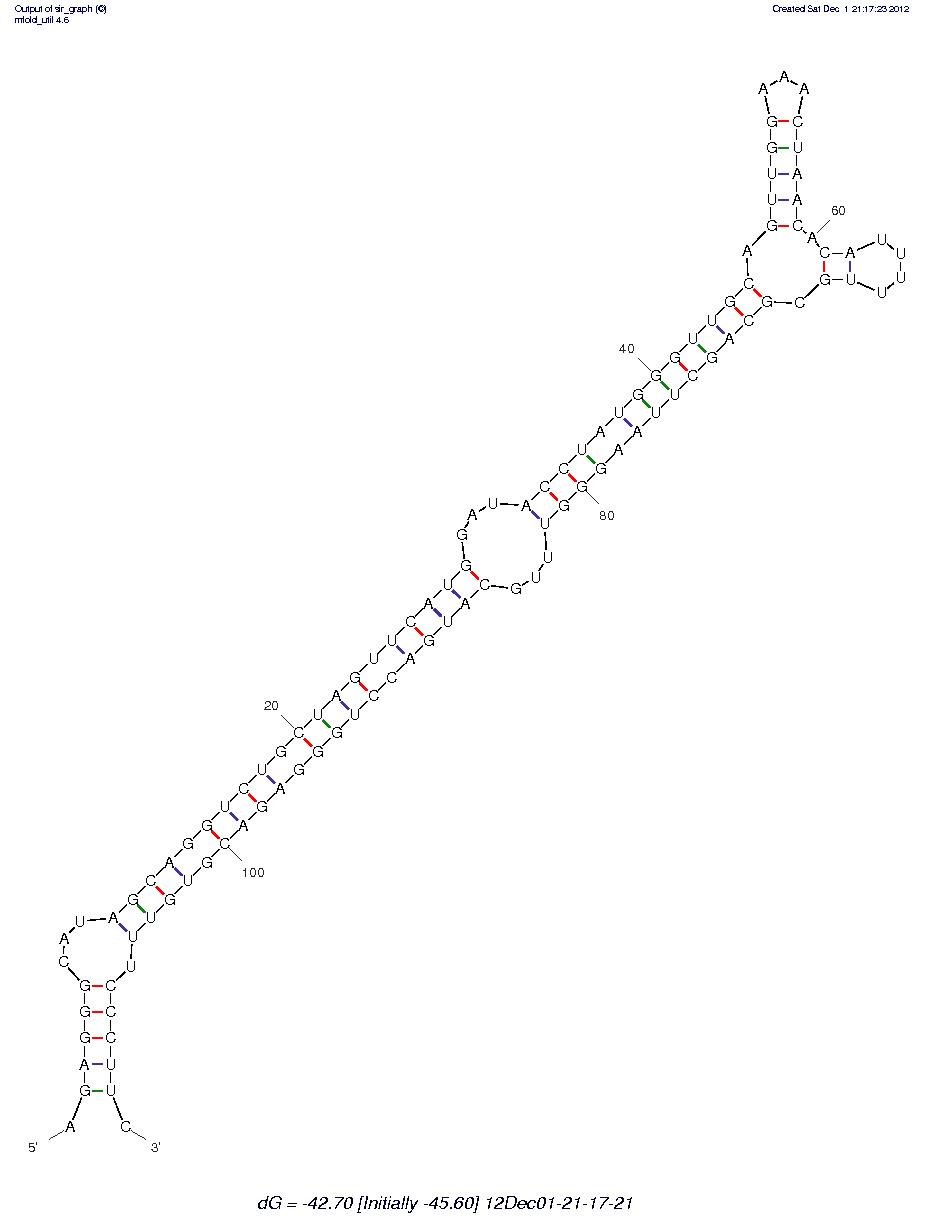


rcomiR044


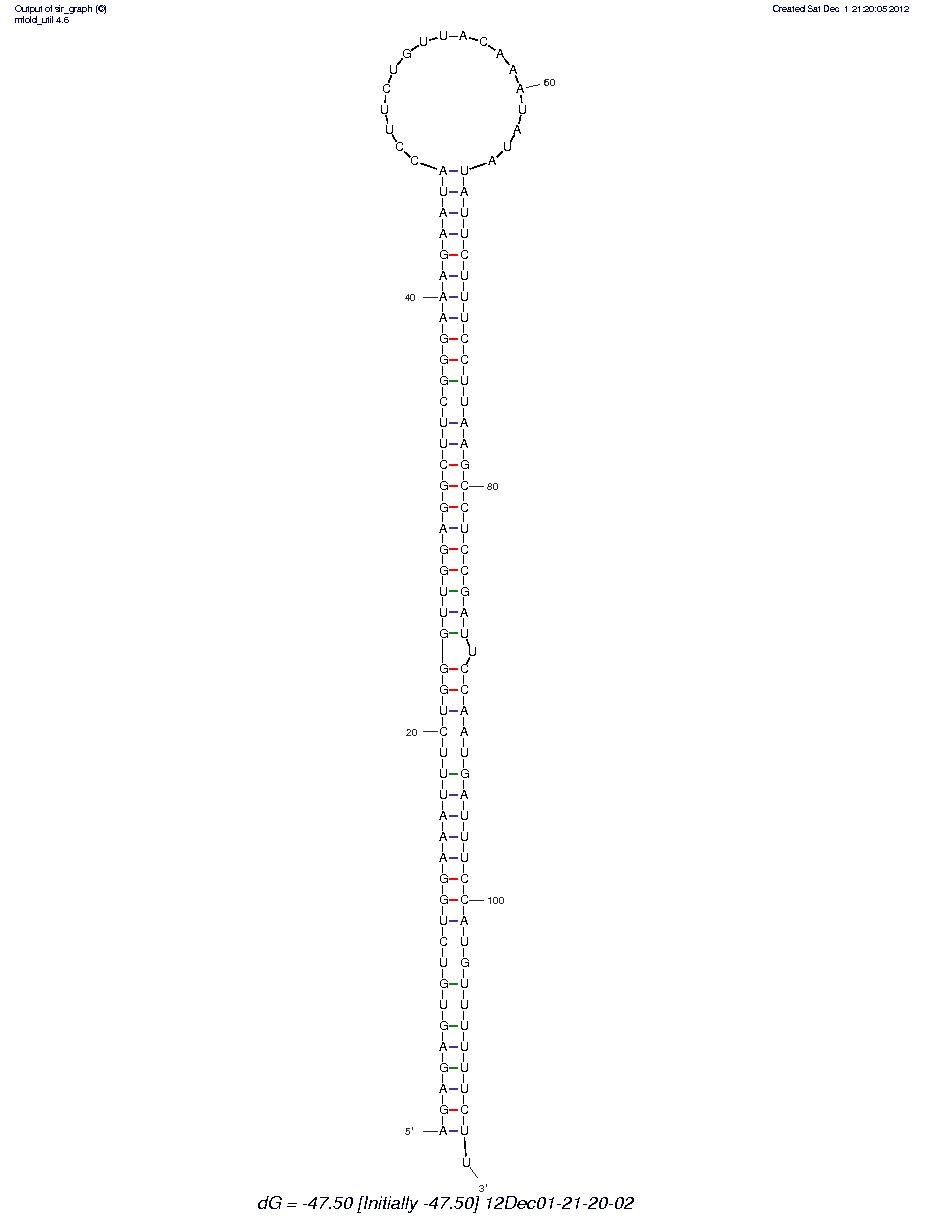


rcomiR045


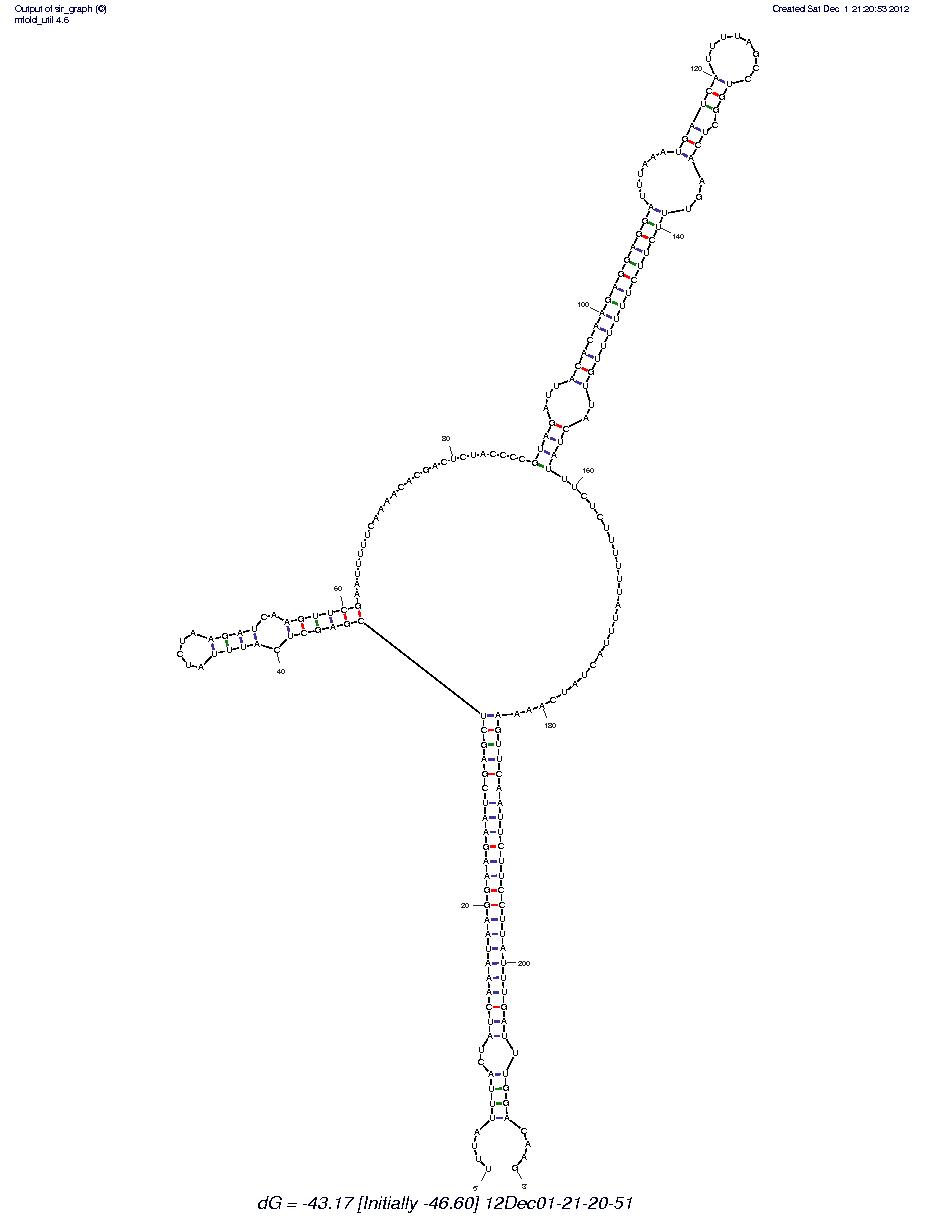


rcomiR046


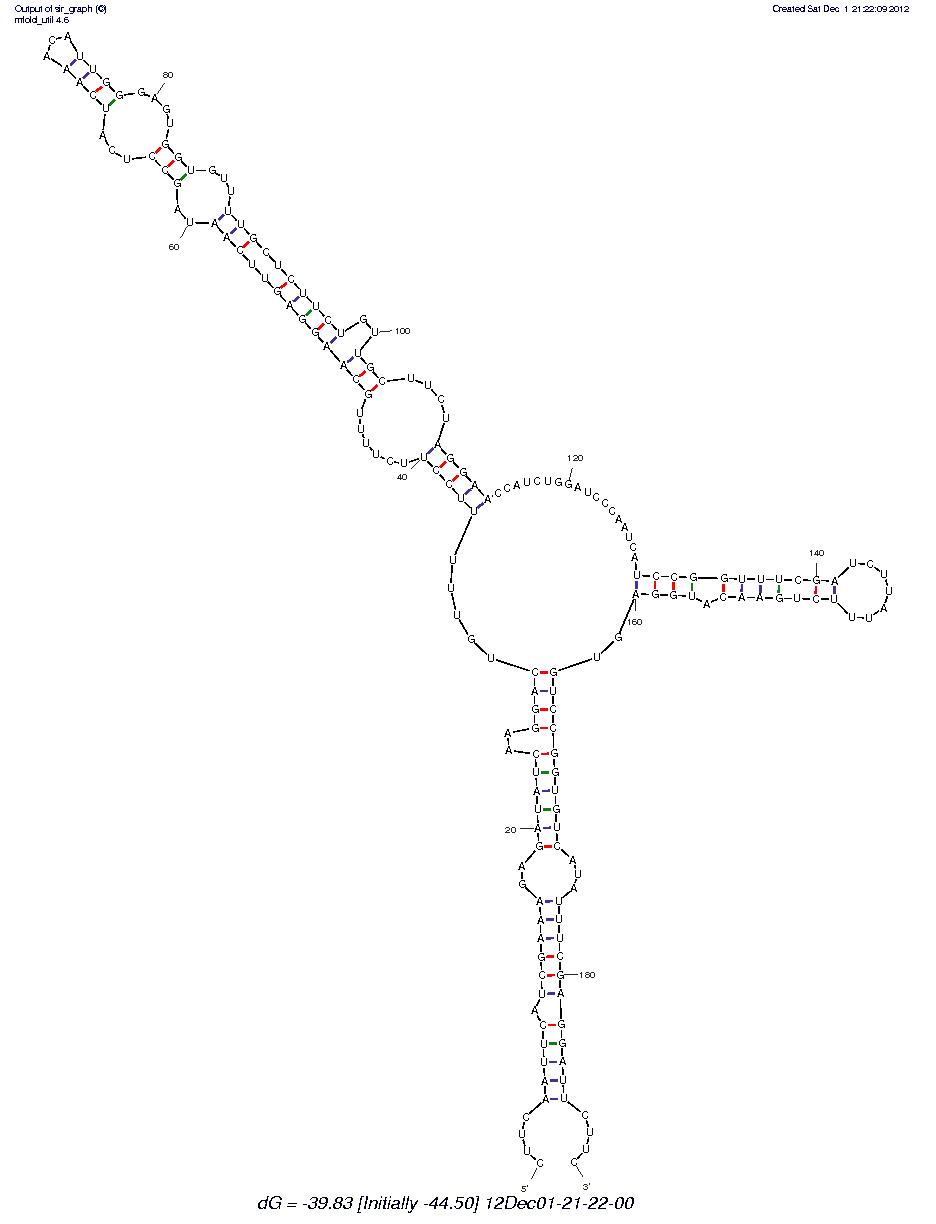


rcomiR047


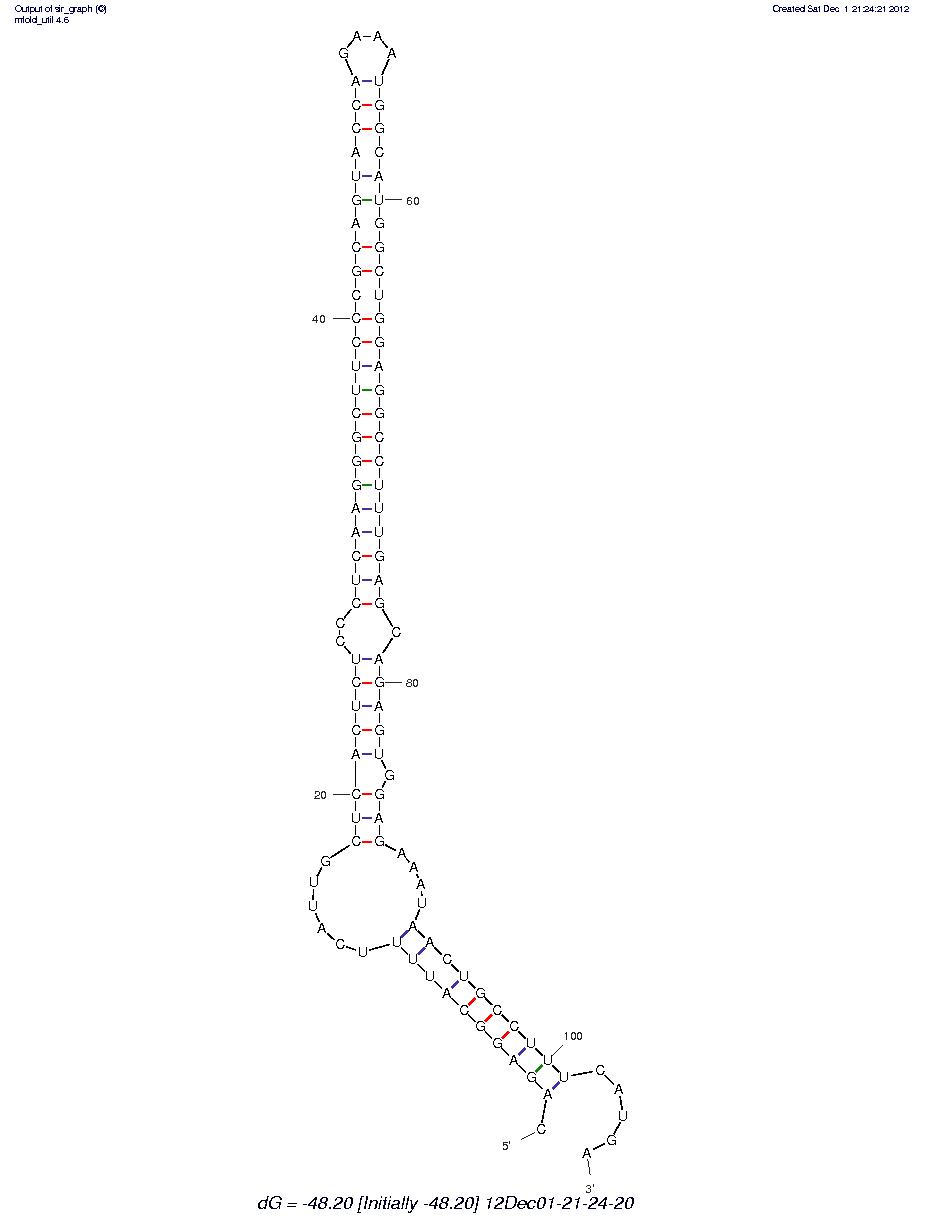


rcomiR048


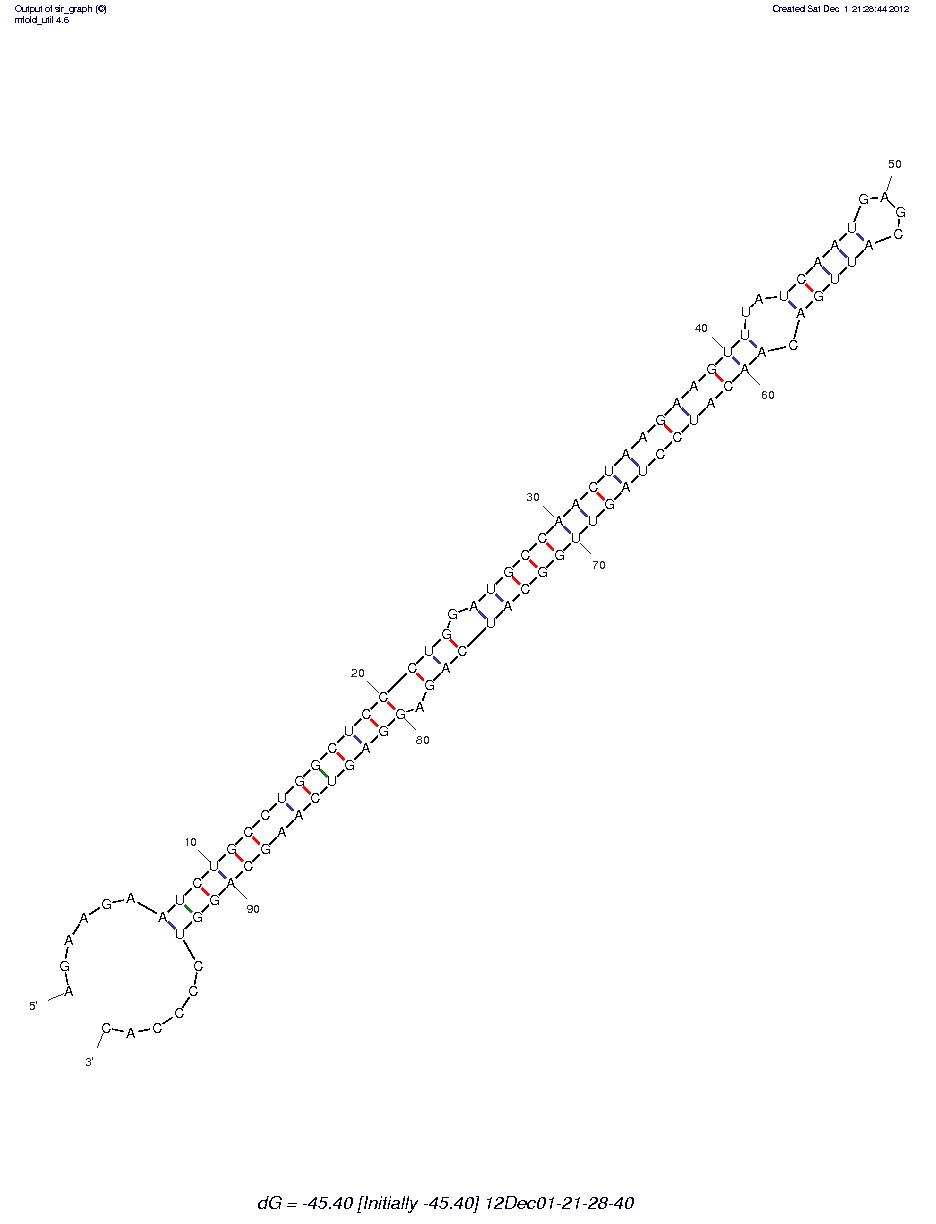


rcomiR049


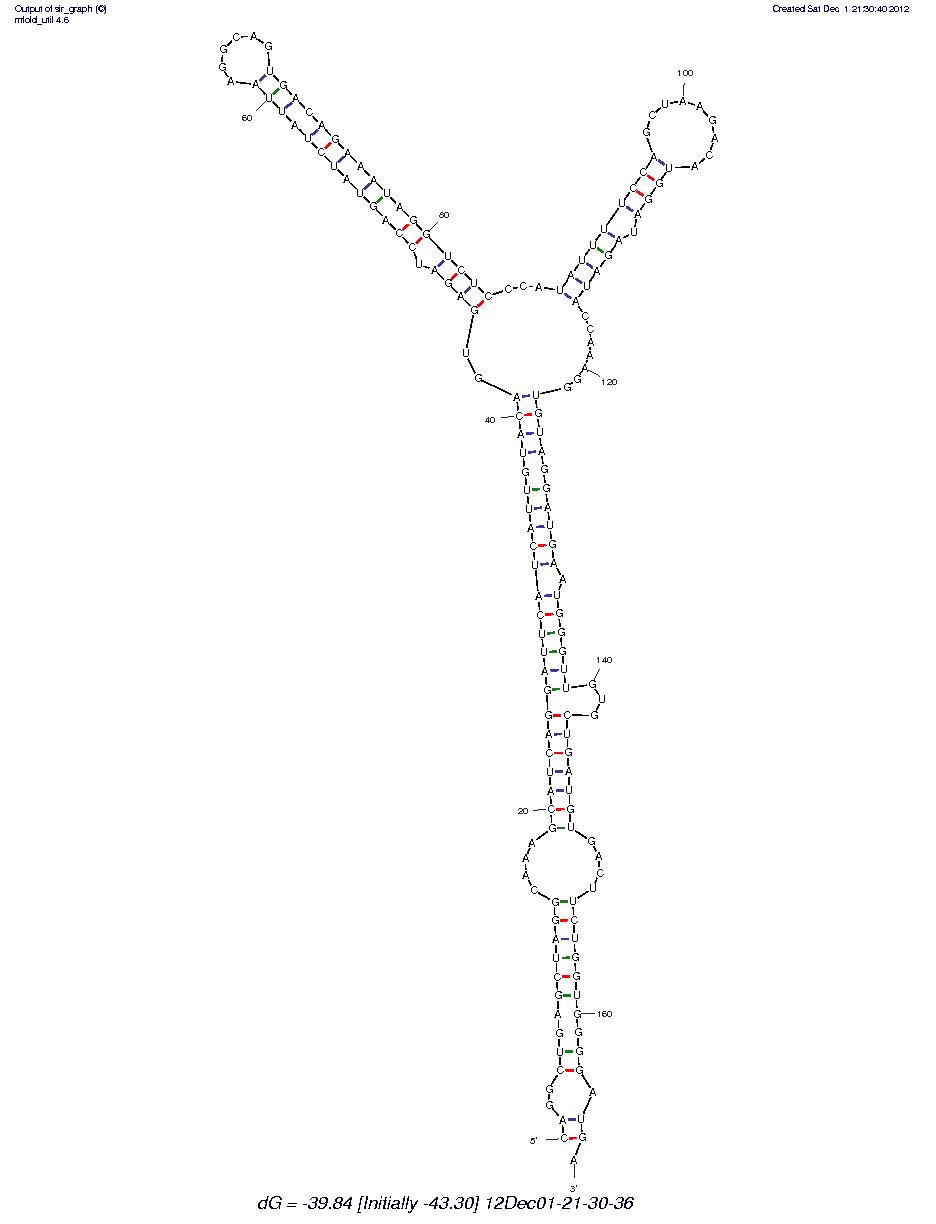


rcomiR050


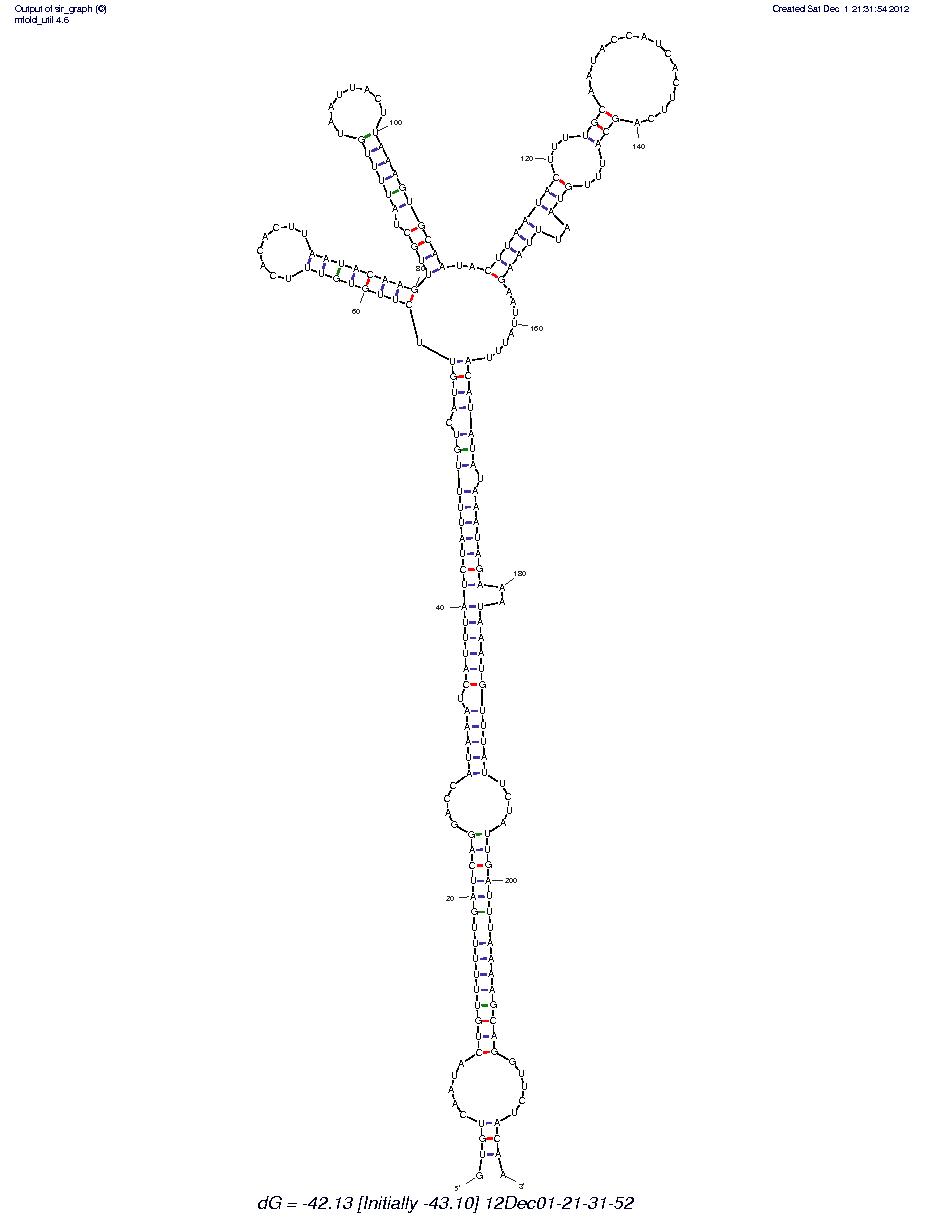


rcomiR051


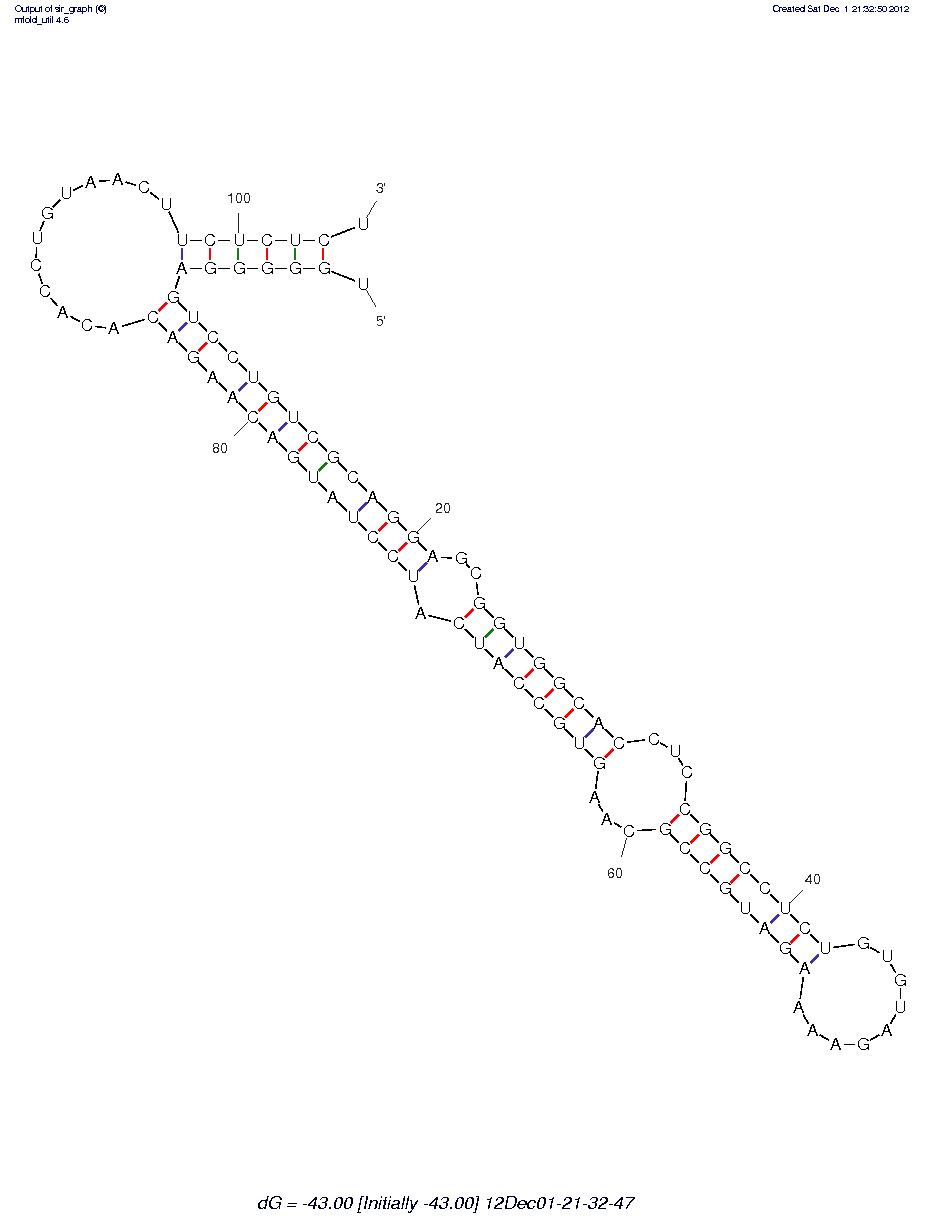


rcomiR052


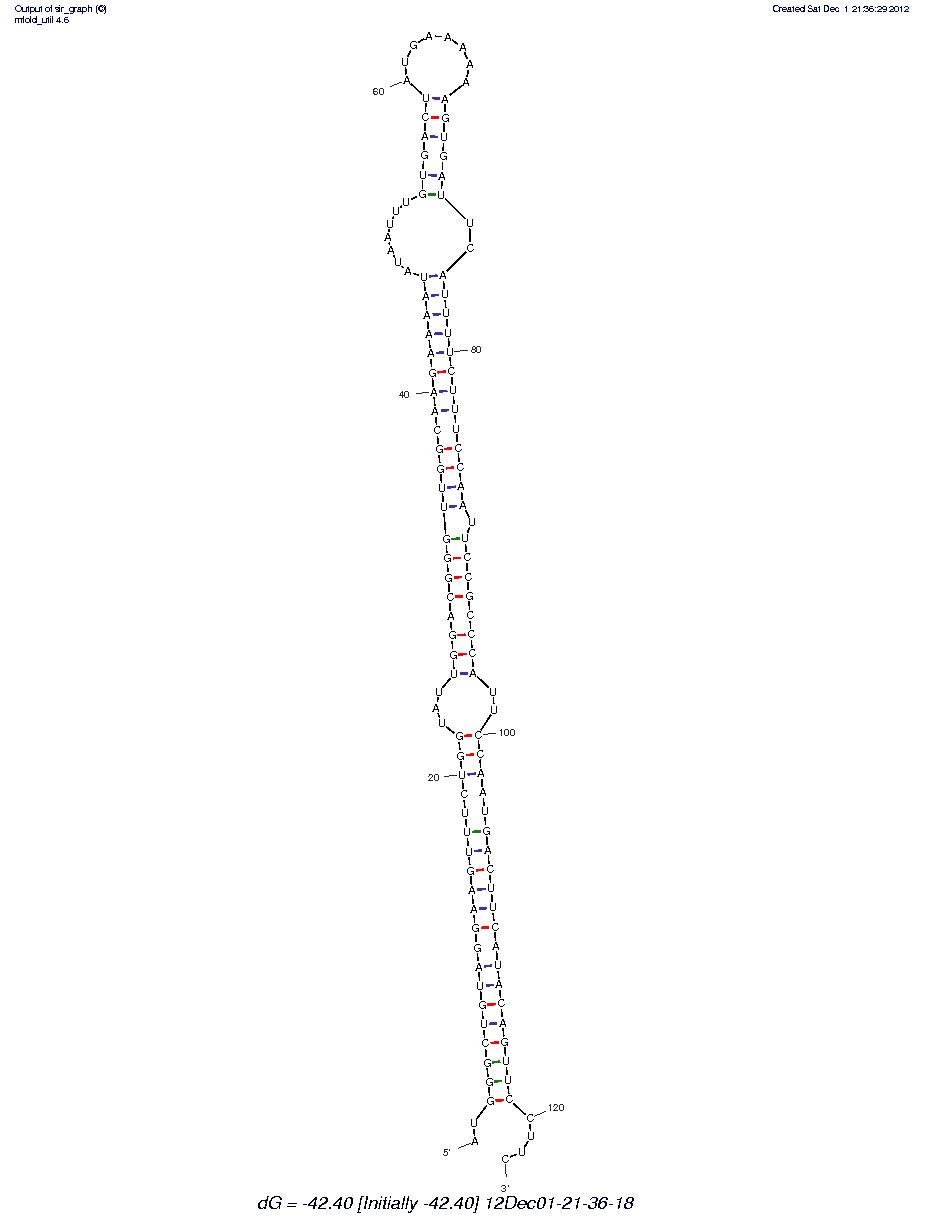


rcomiR053


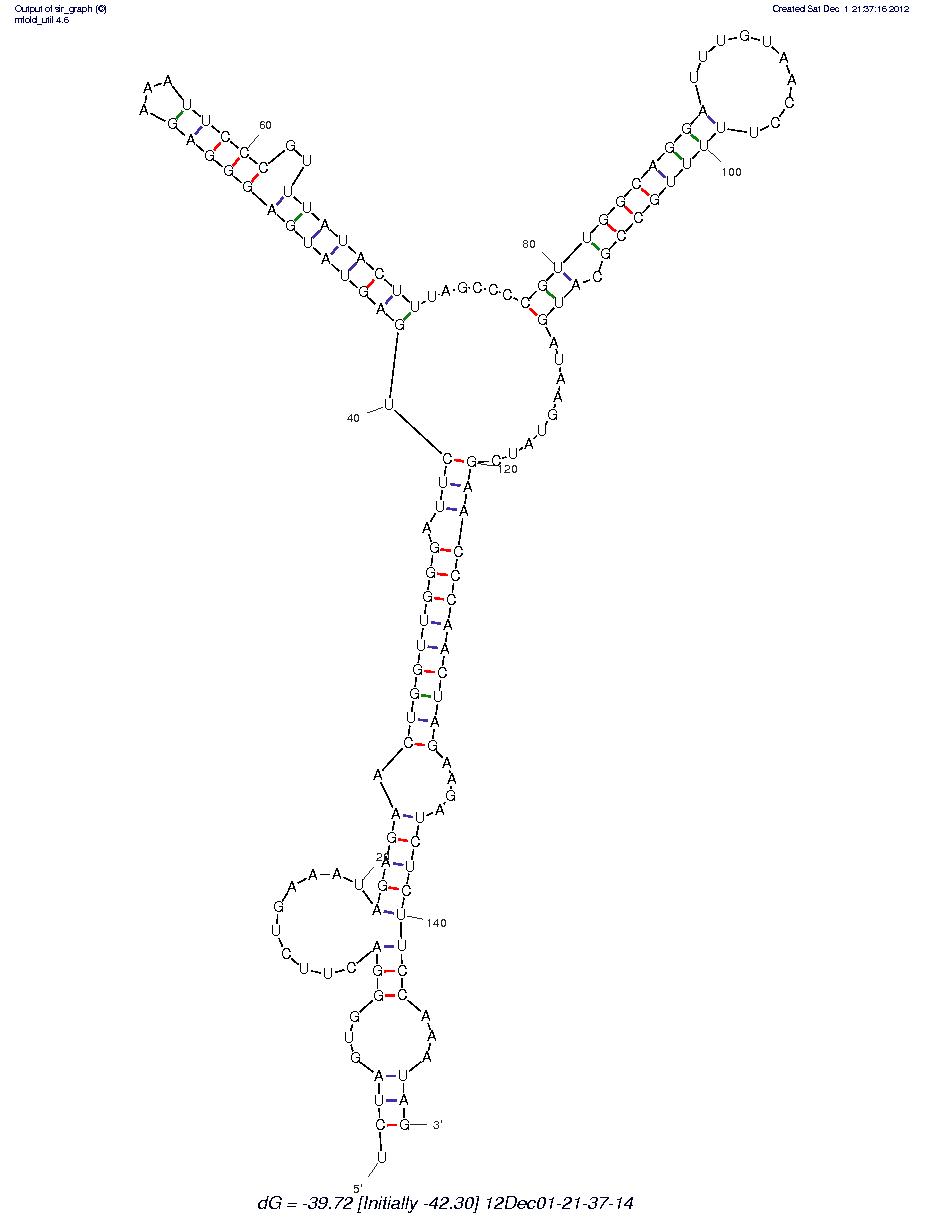


rcomiR054


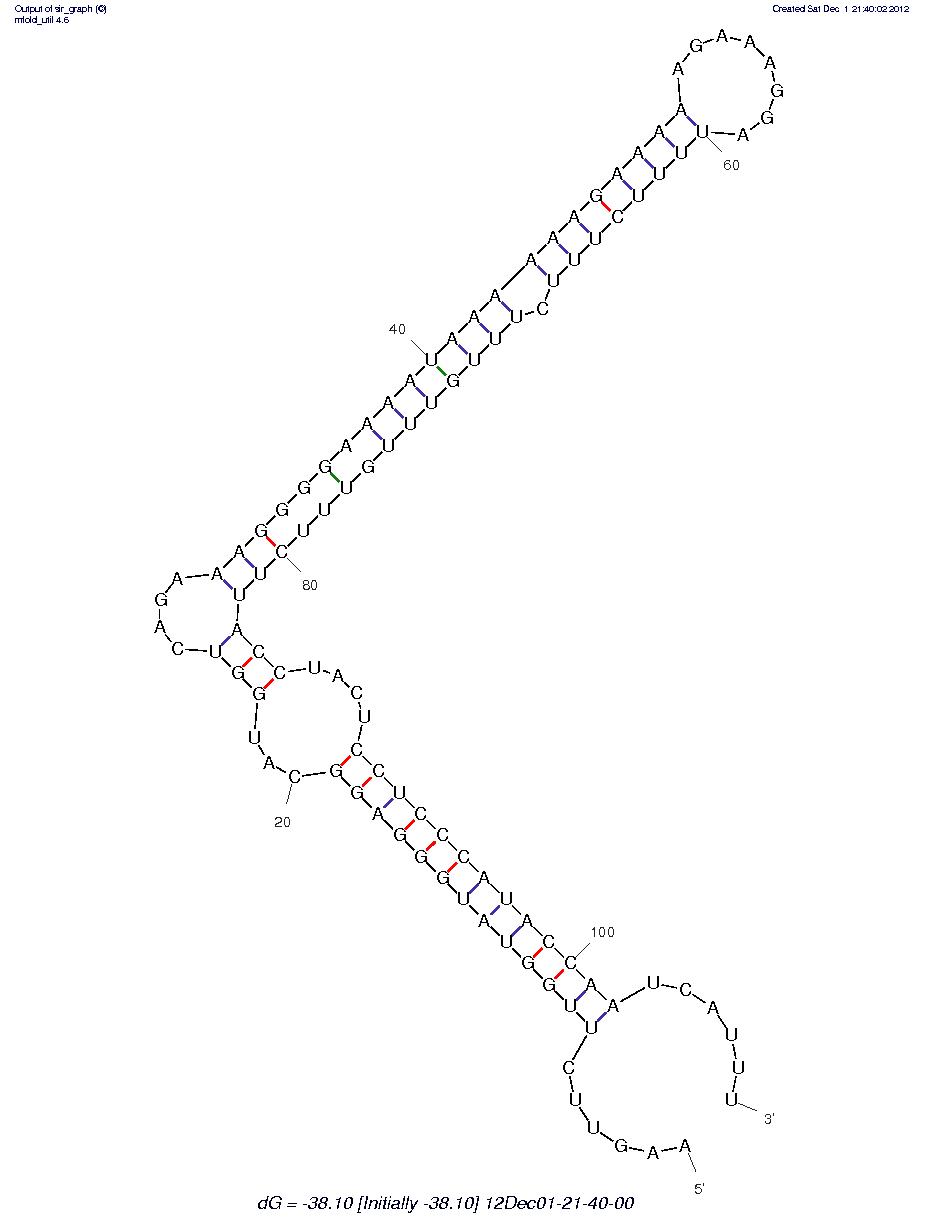


rcomiR055


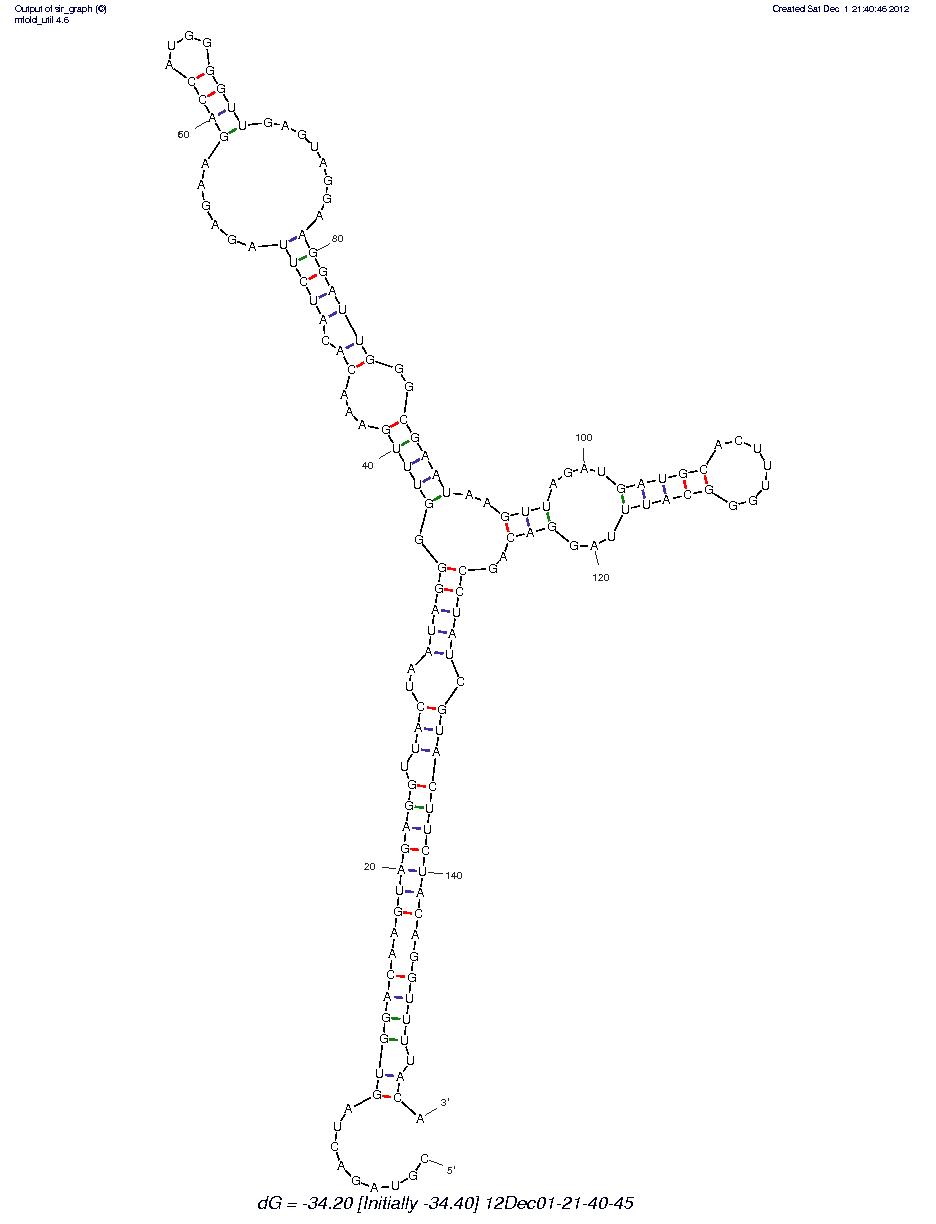


rcomiR056


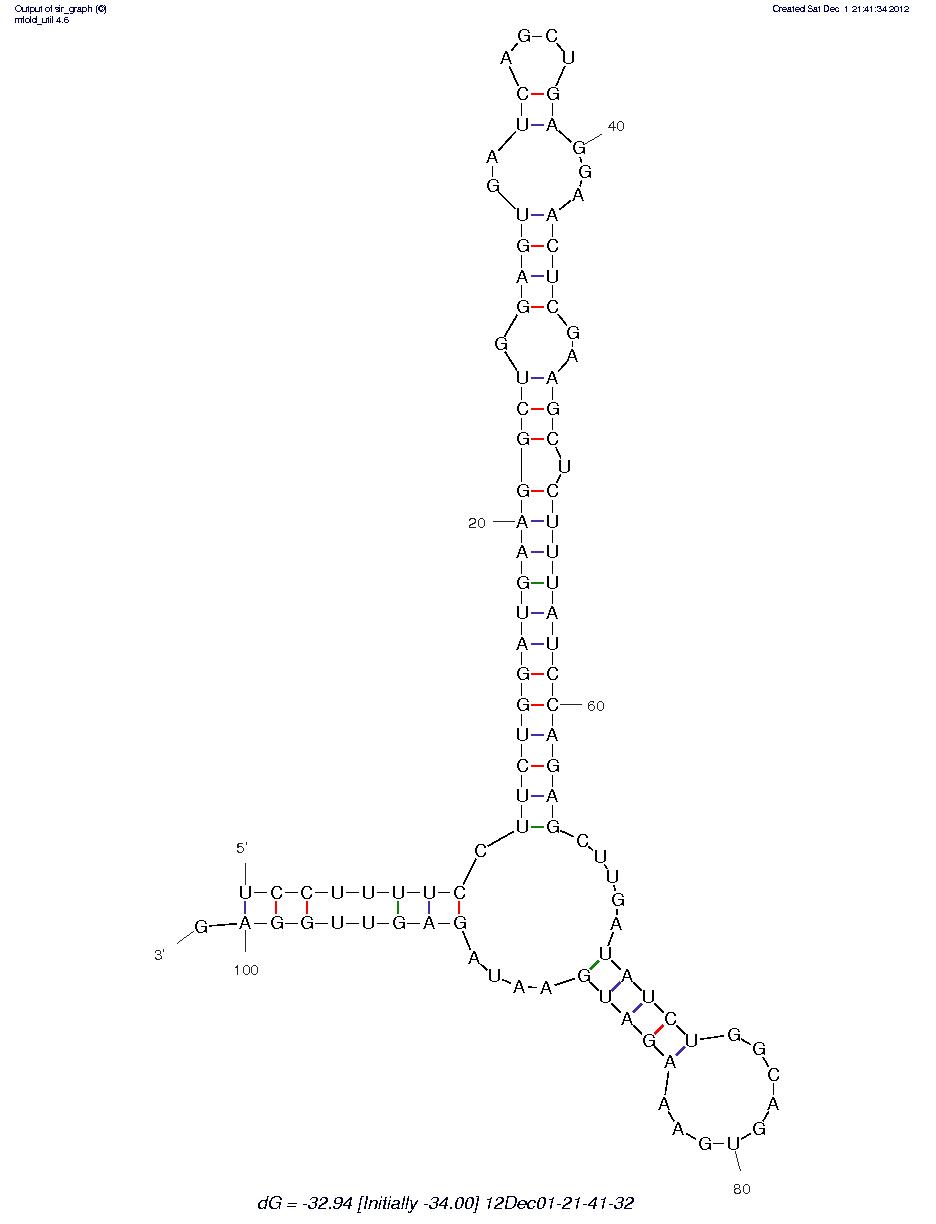


rcomiR057


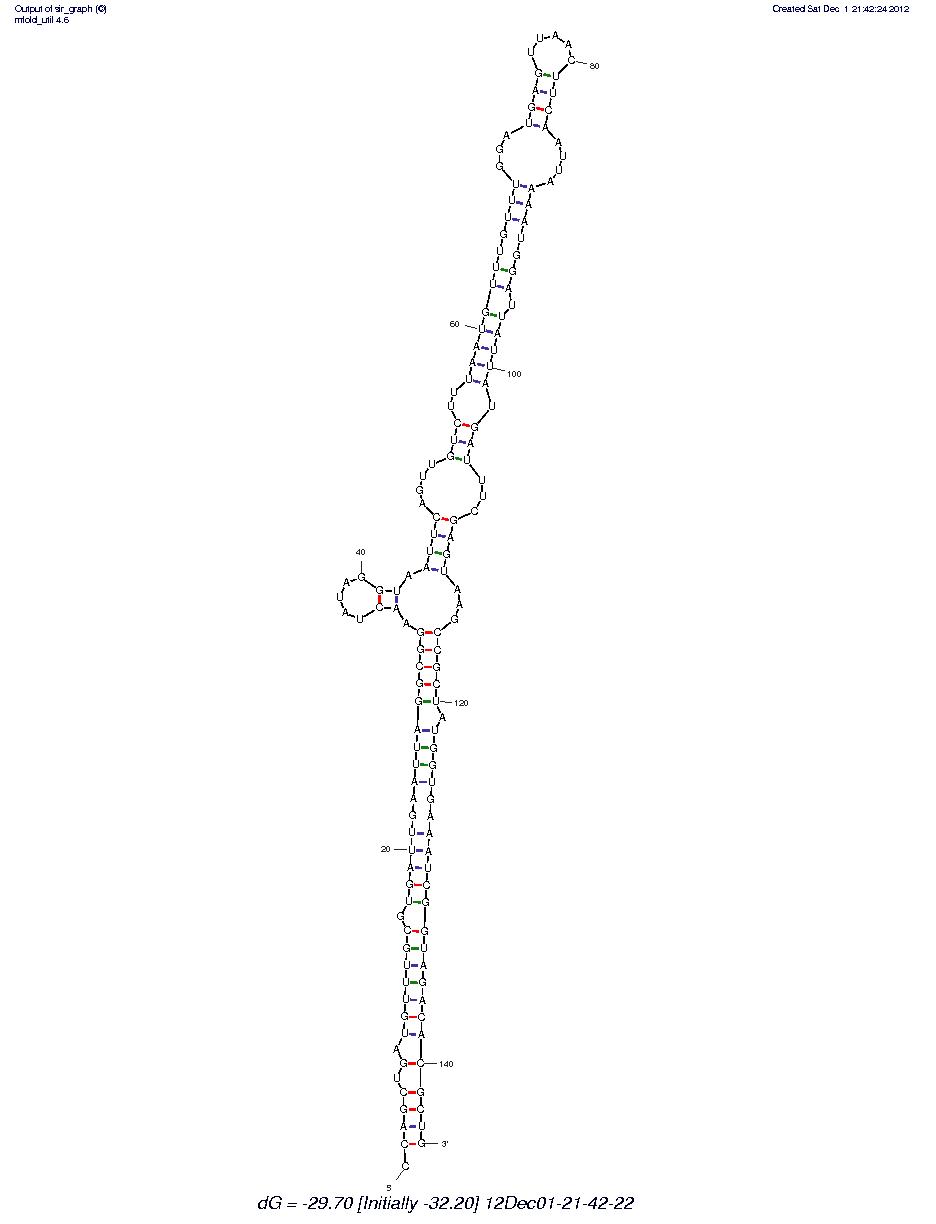


rcomiR058


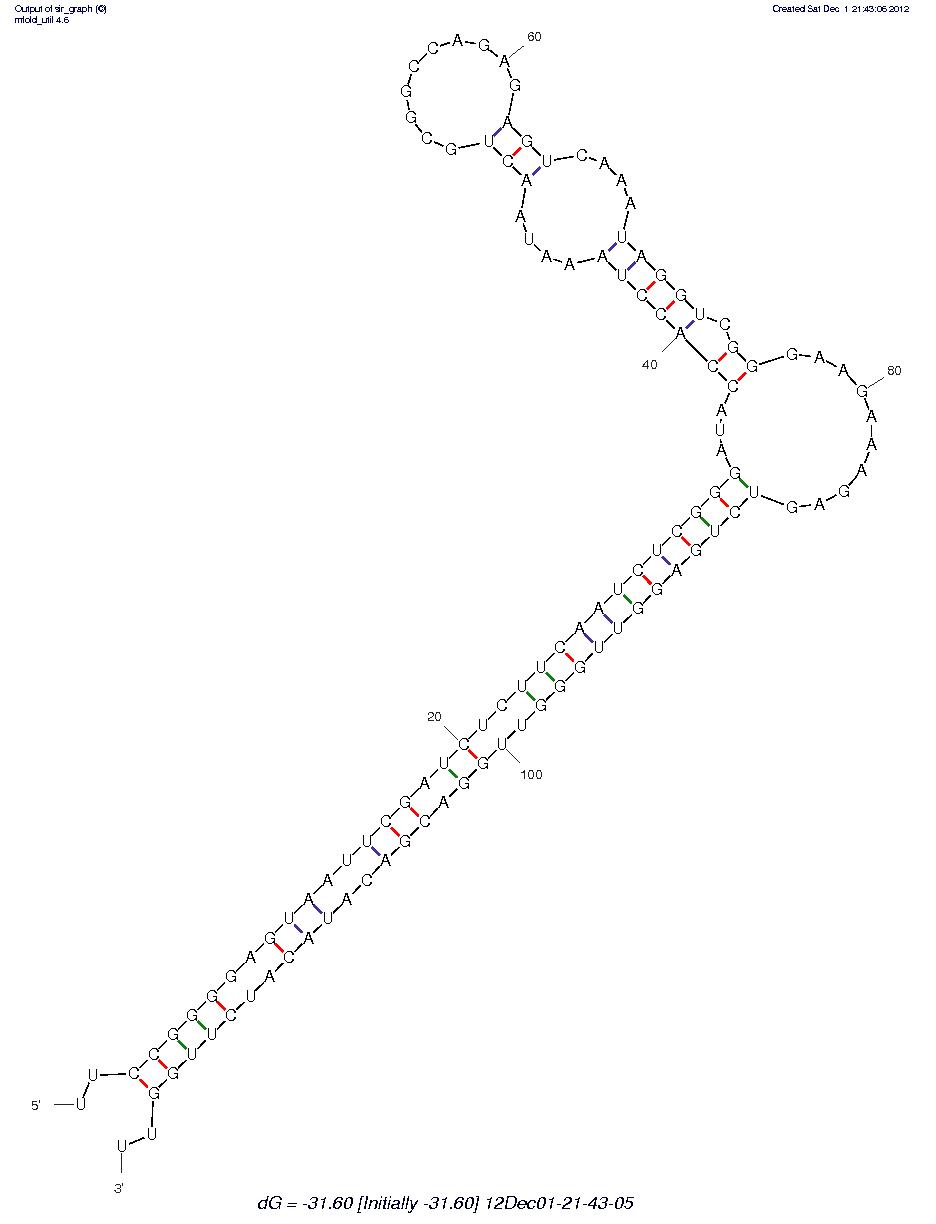


rcomiR059


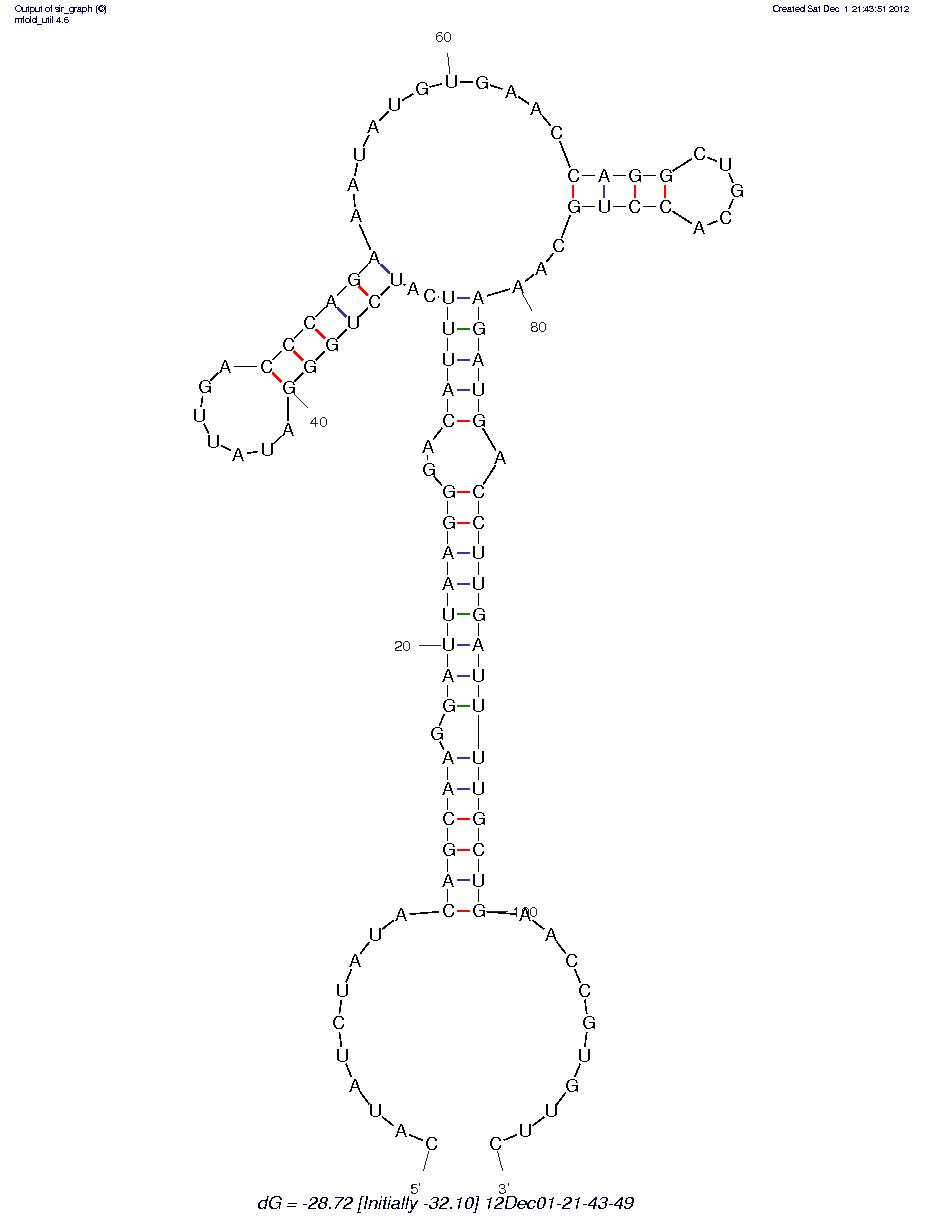


rcomiR060
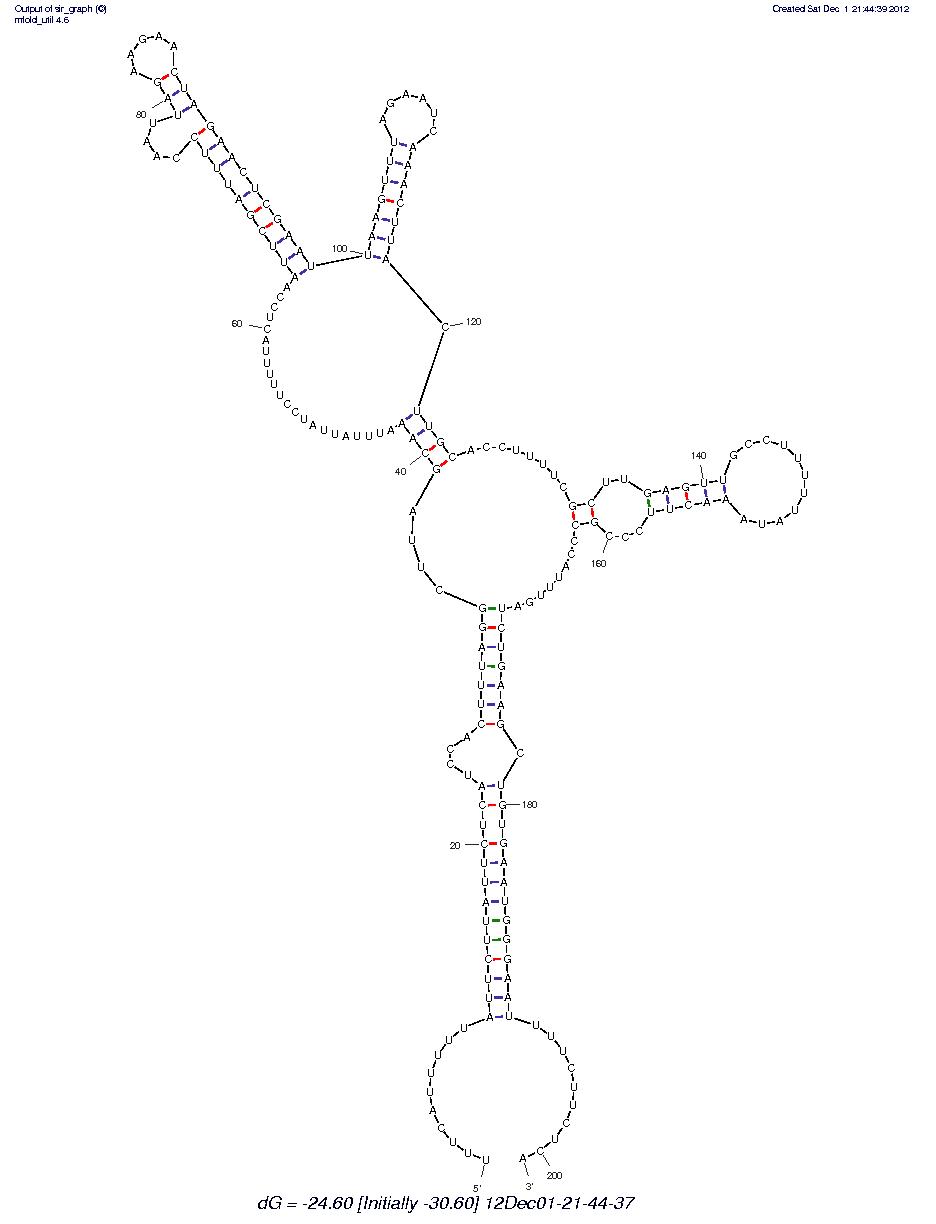


rcomiR061


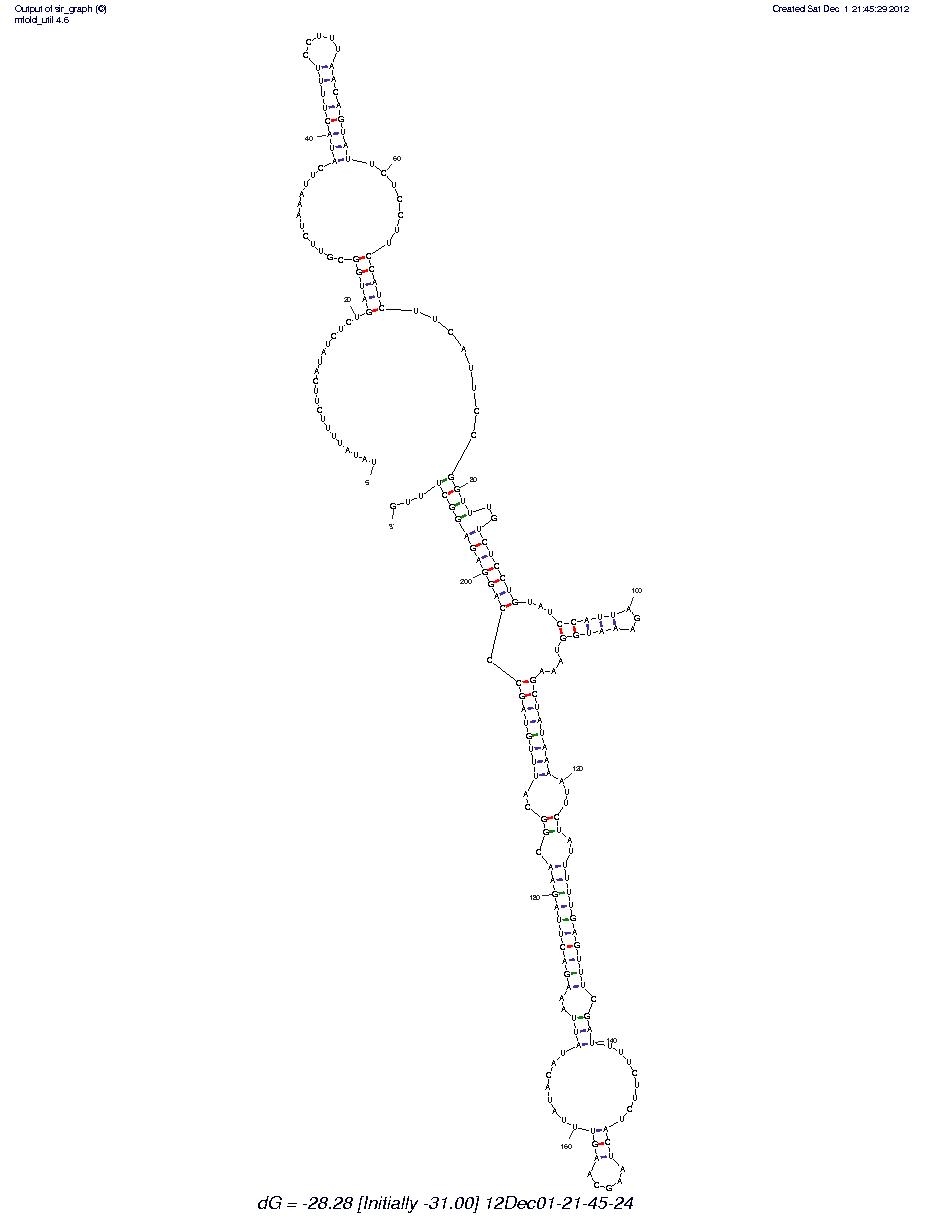


rcomiR062


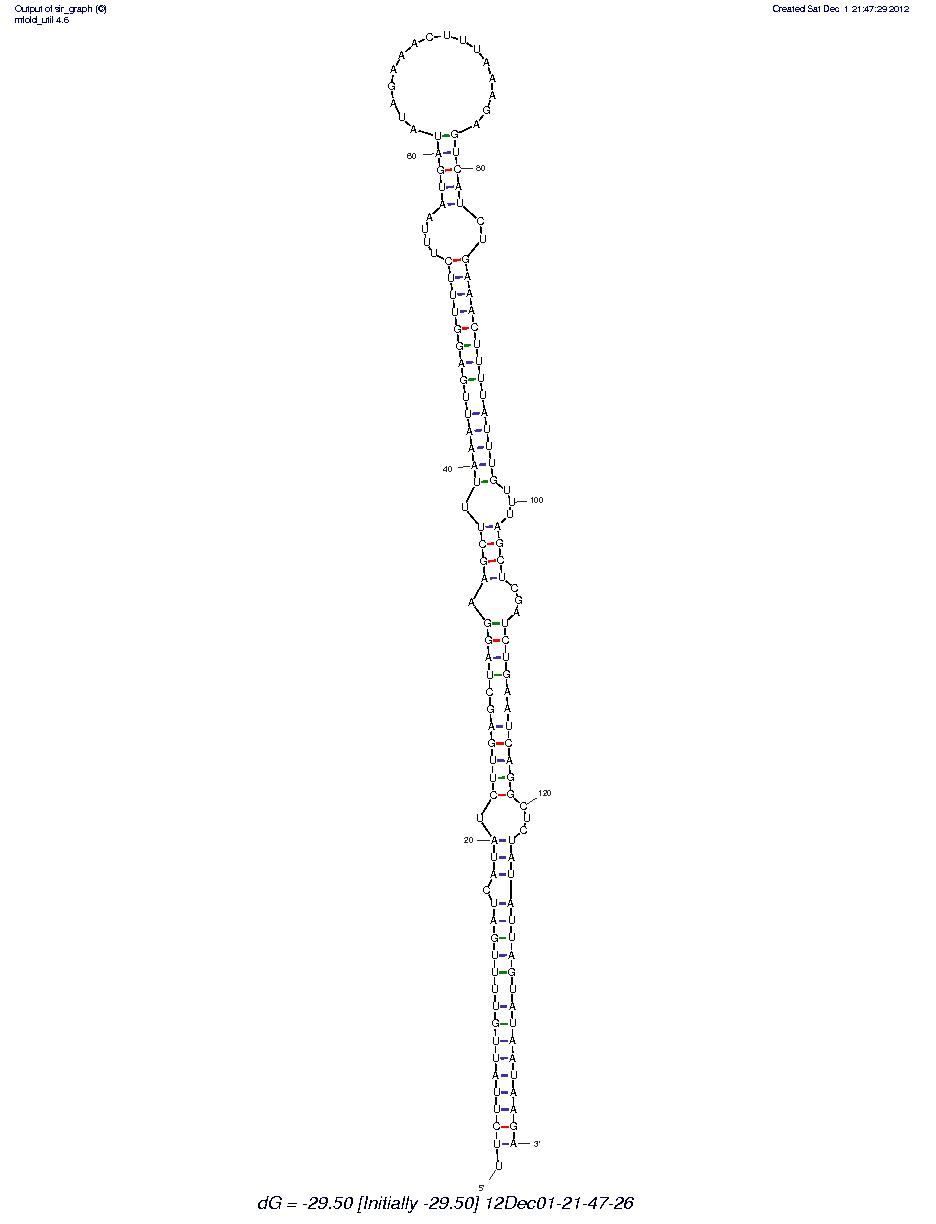


rcomiR063


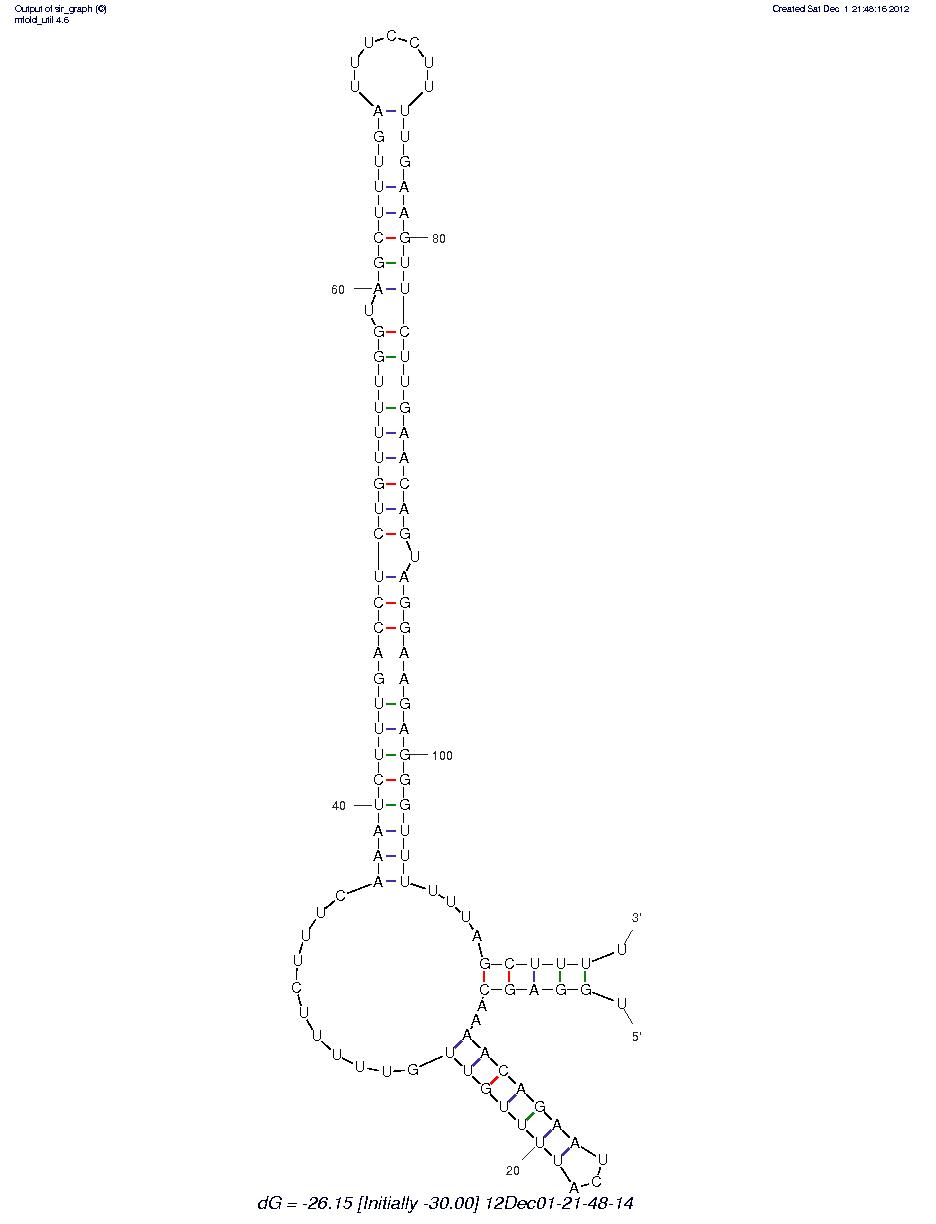


rcomiR064


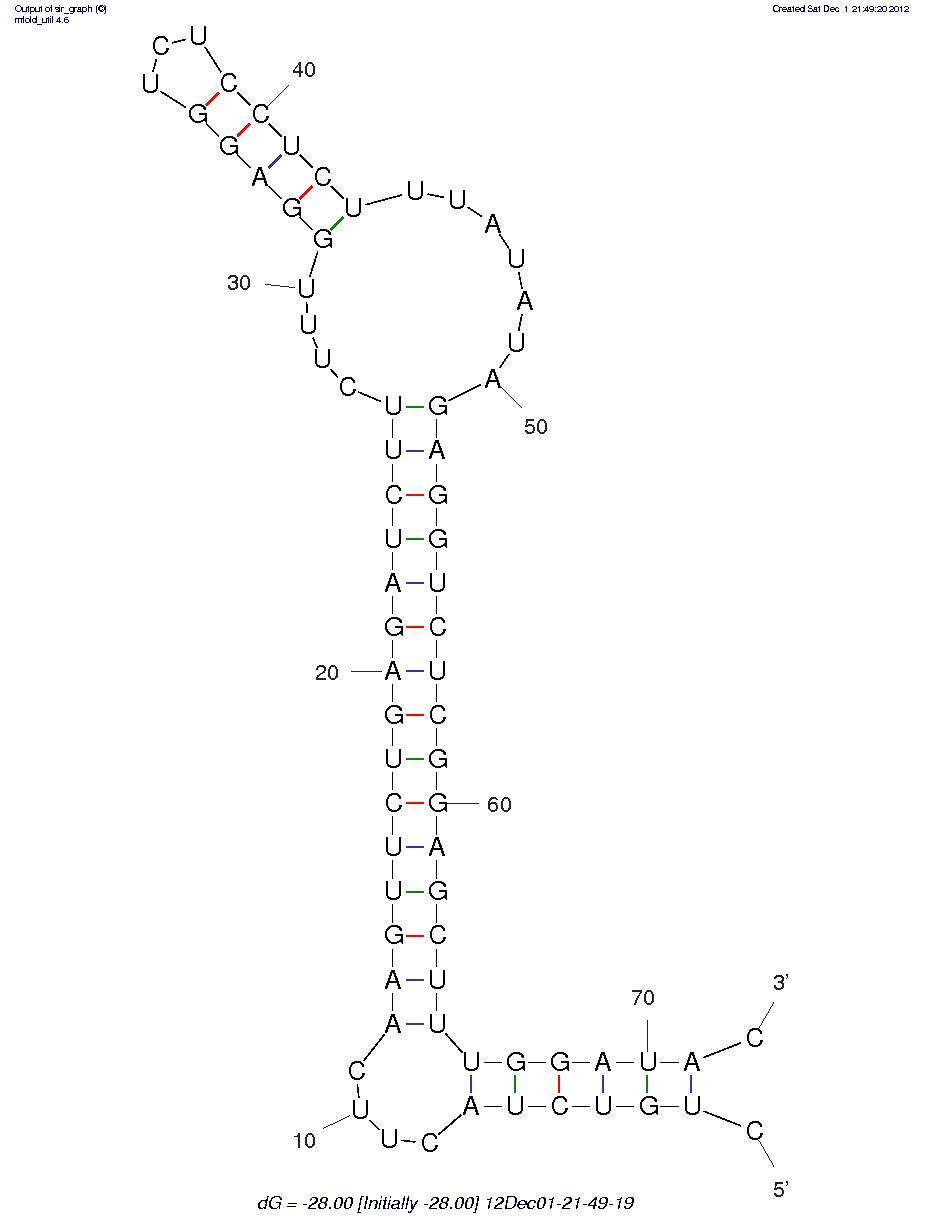


rcomiR065


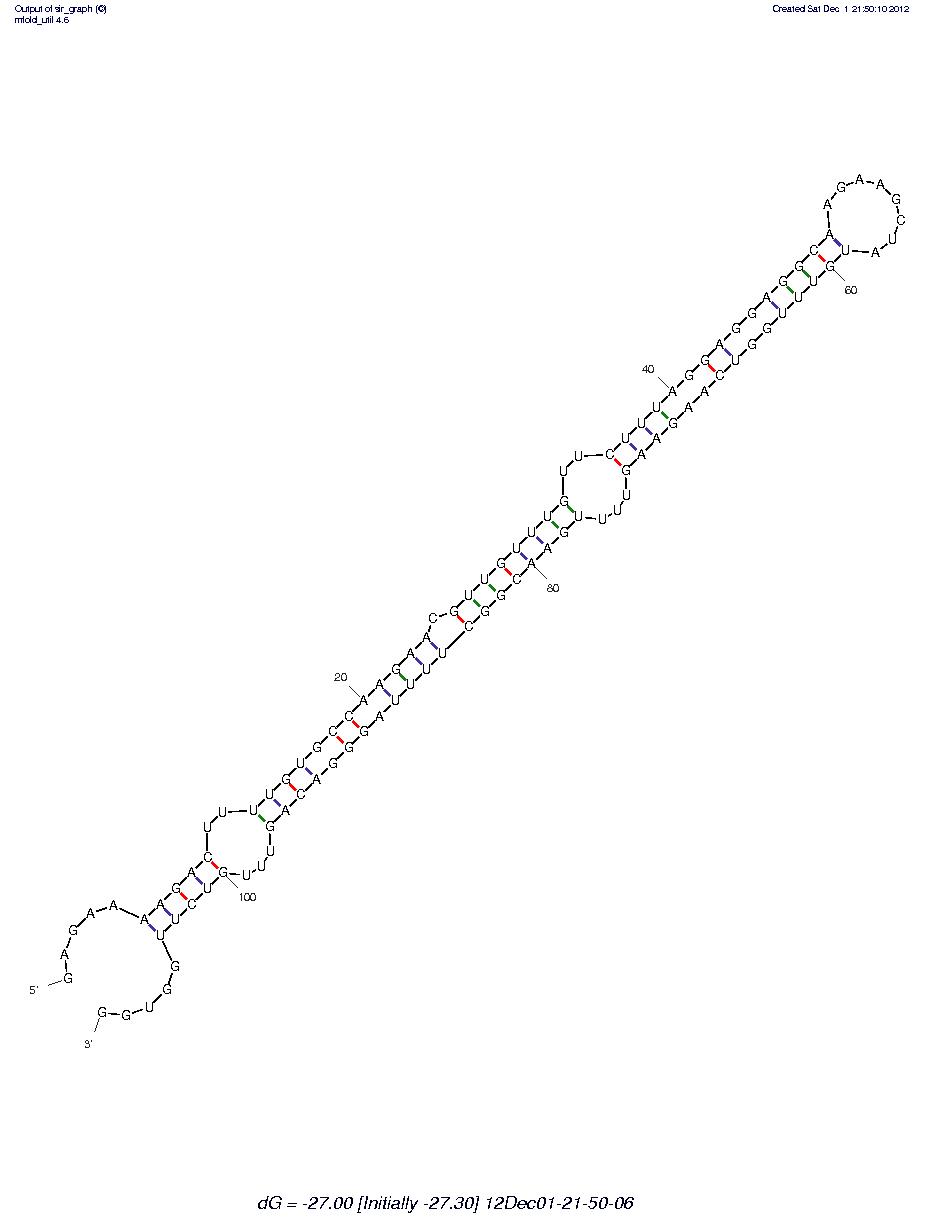


rcomiR066


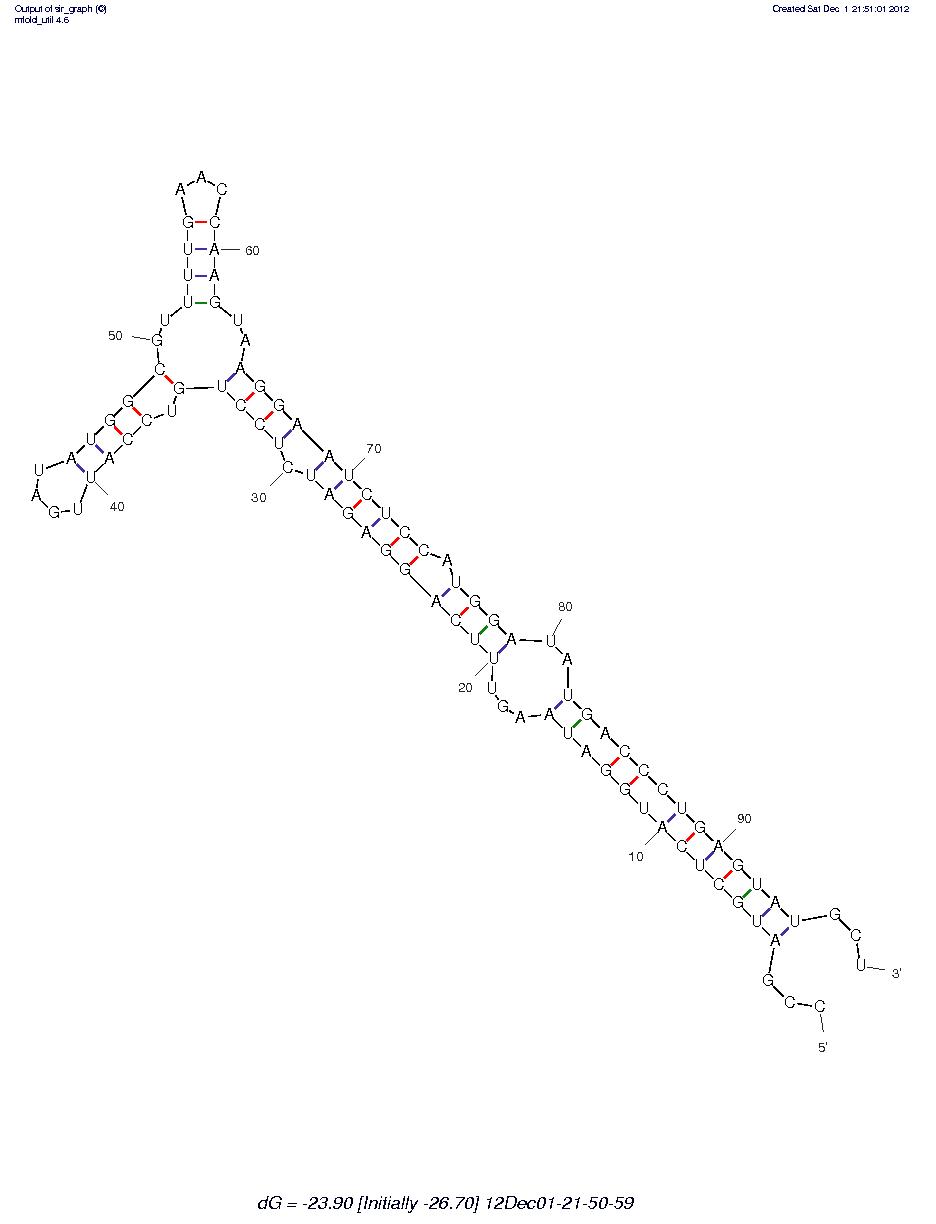


rcomiR067


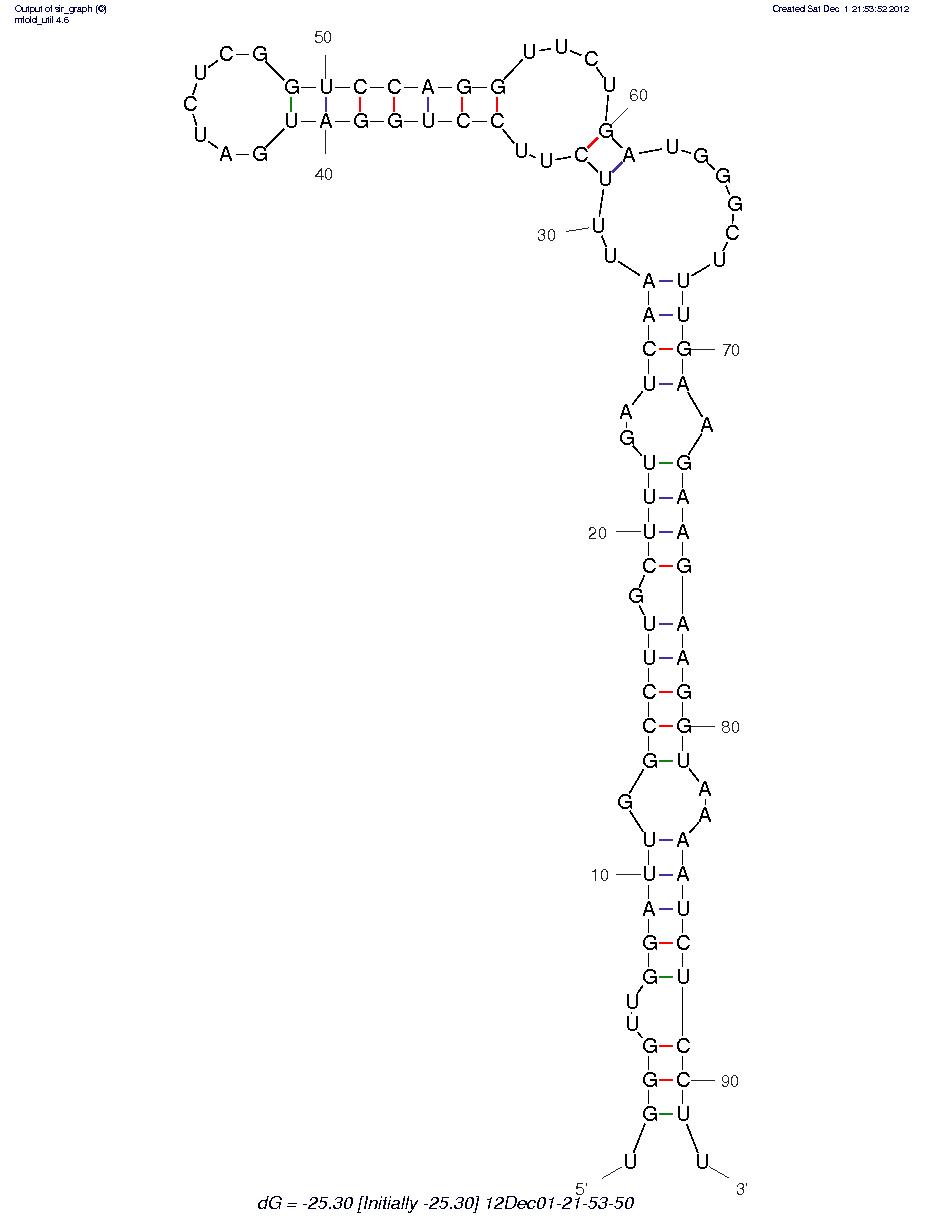


rcomiR068


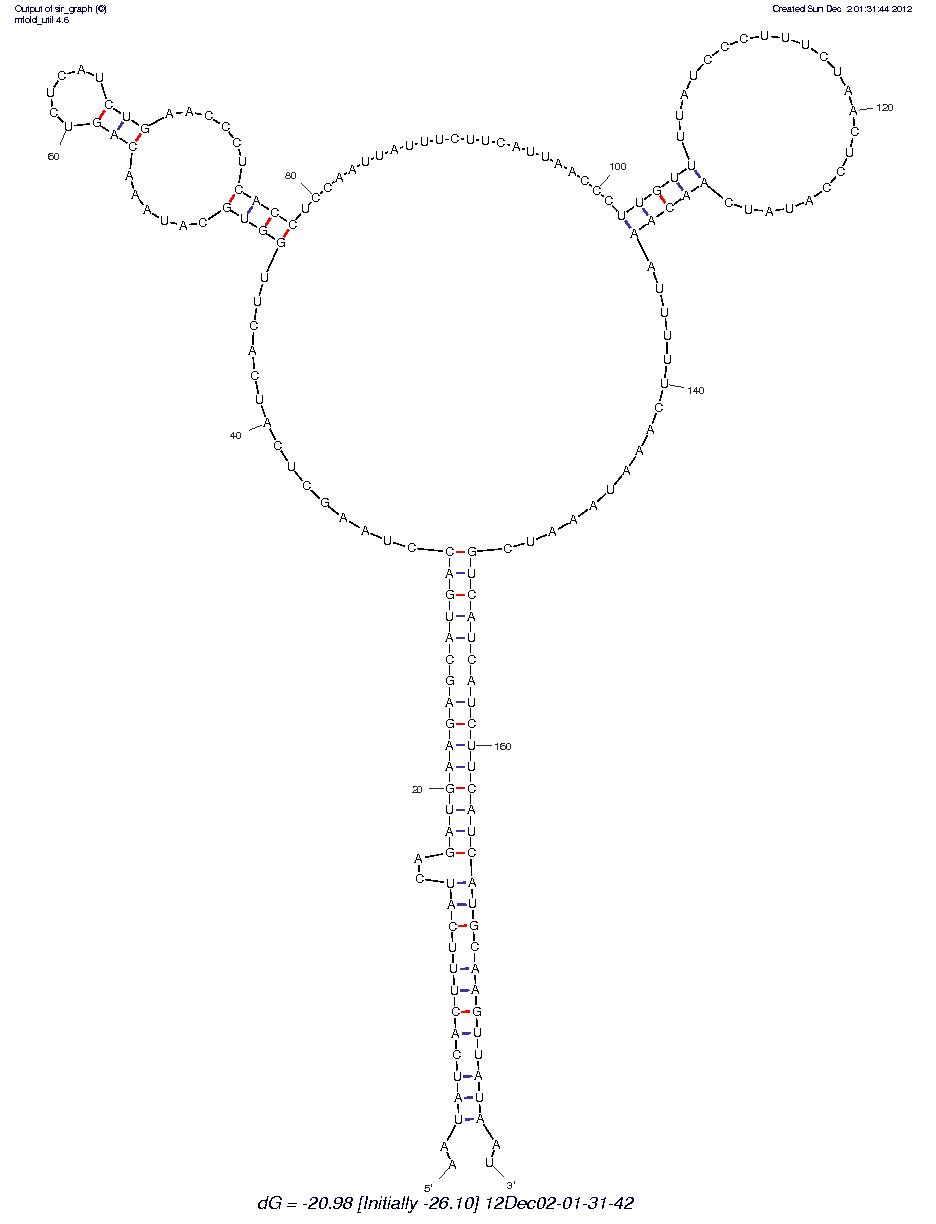


rcomiR069


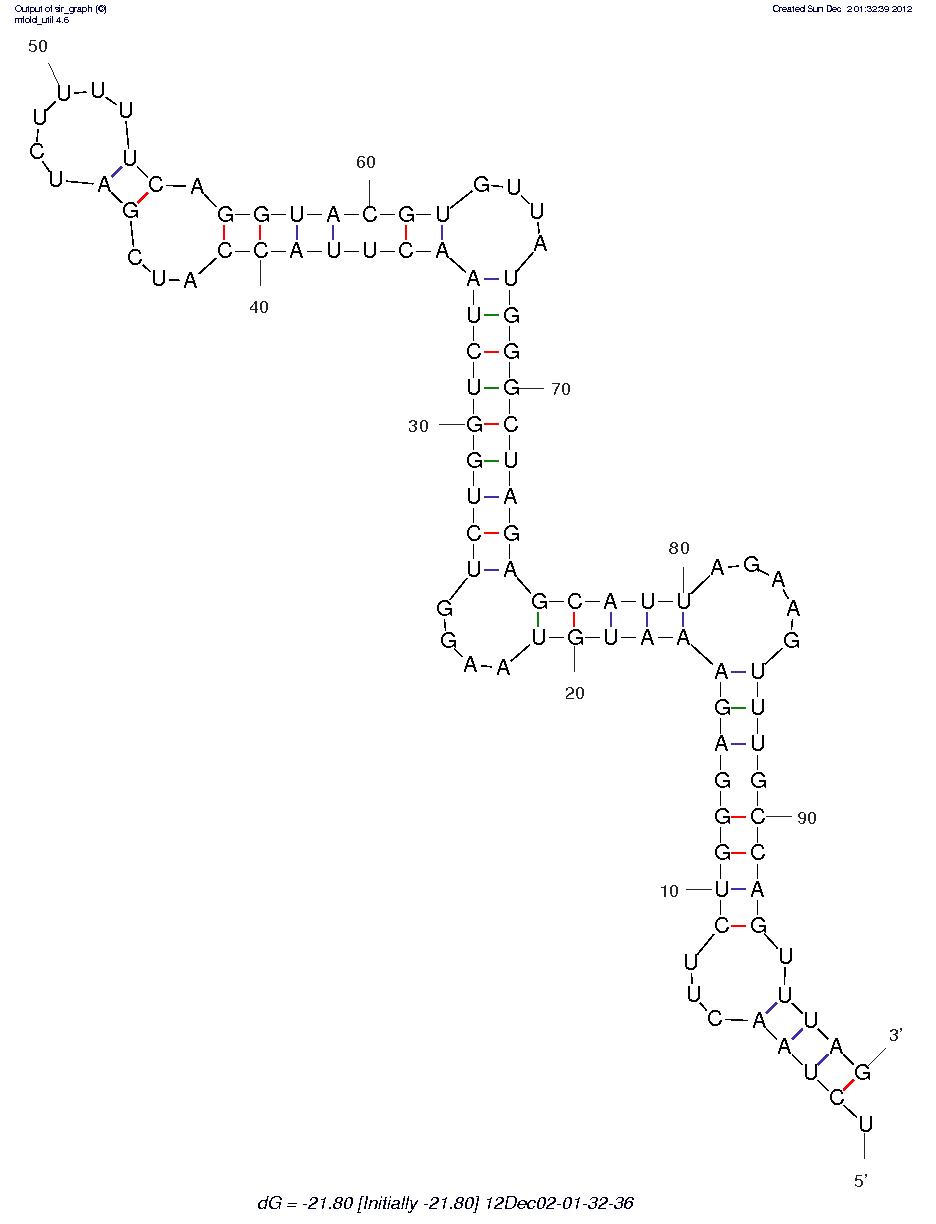


rcomiR070


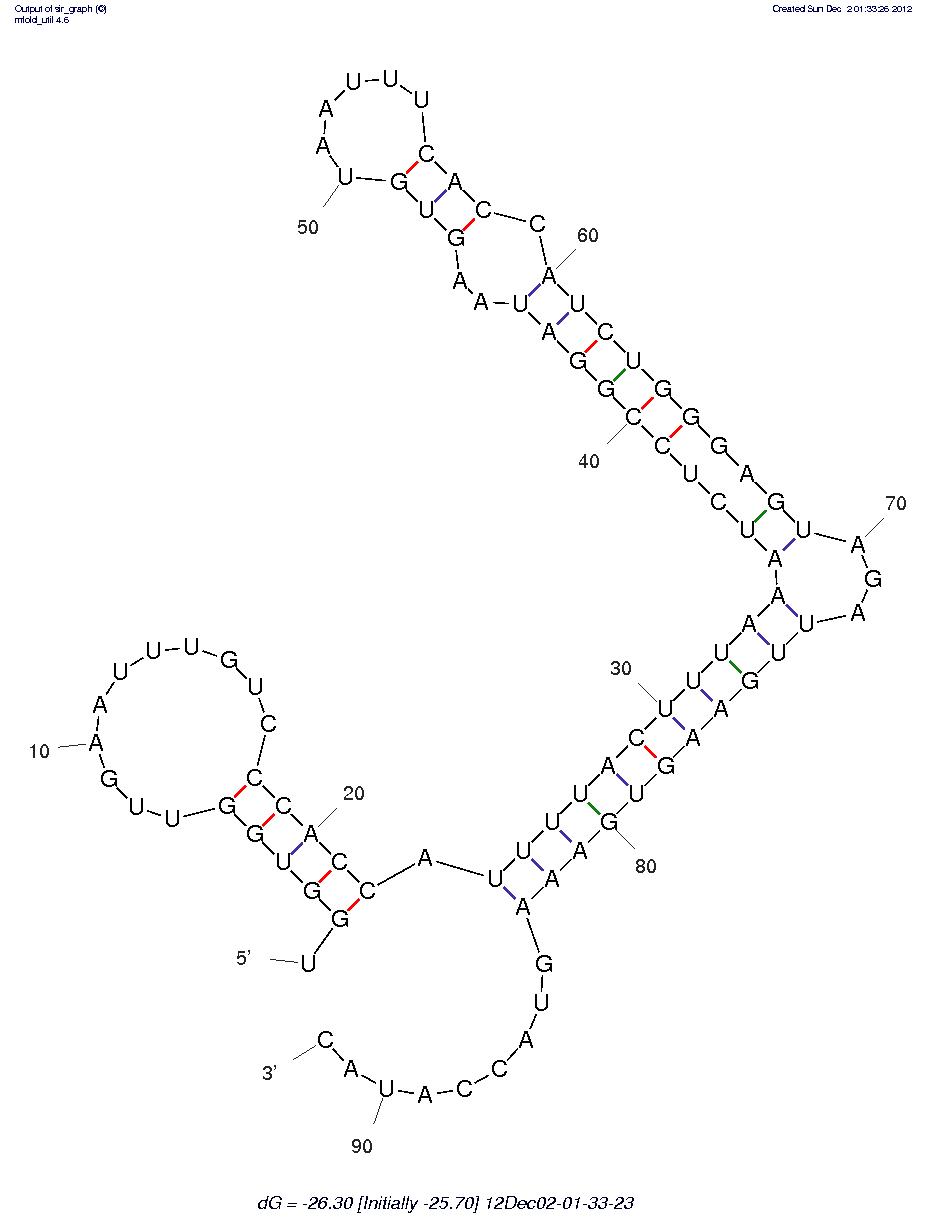


rcomiR071


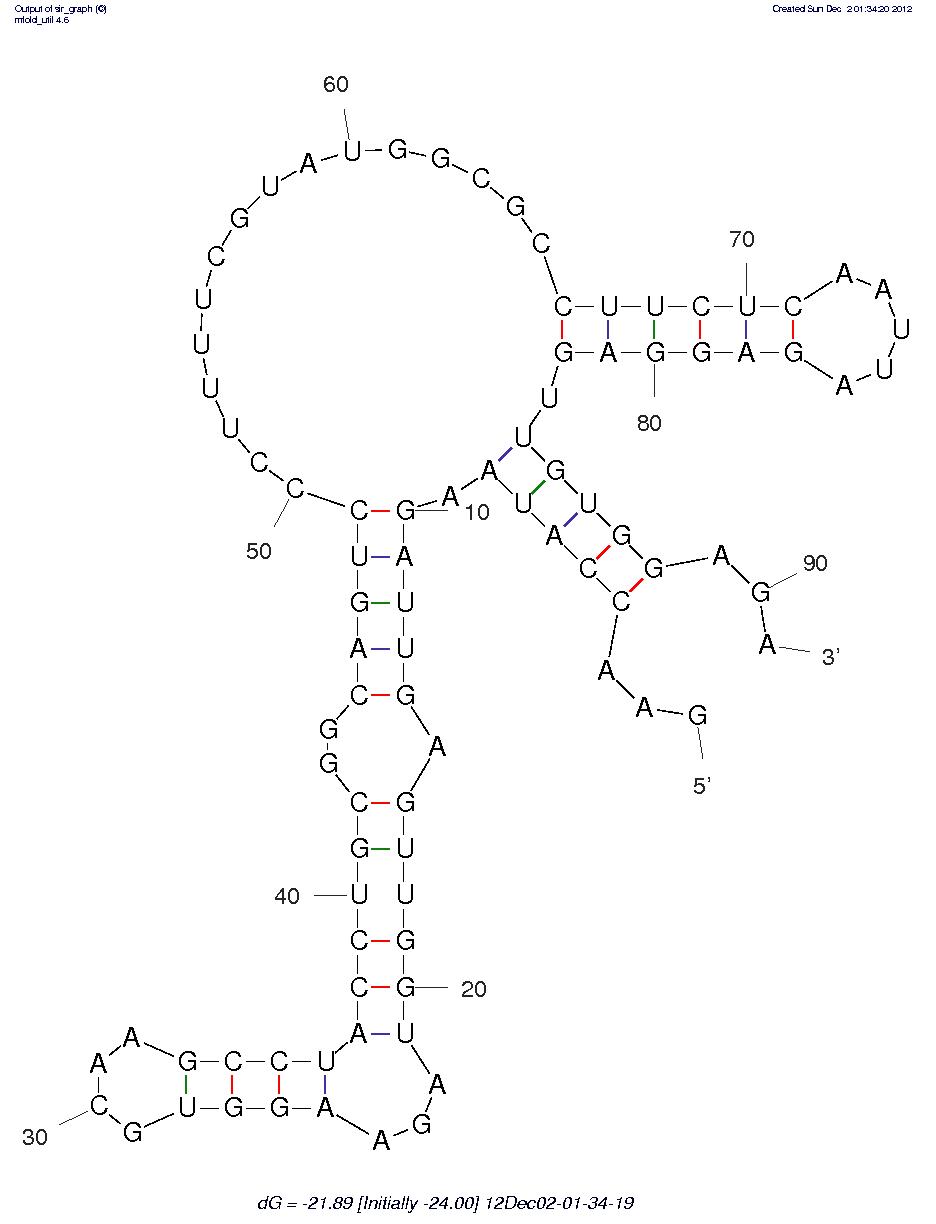


rcomiR072


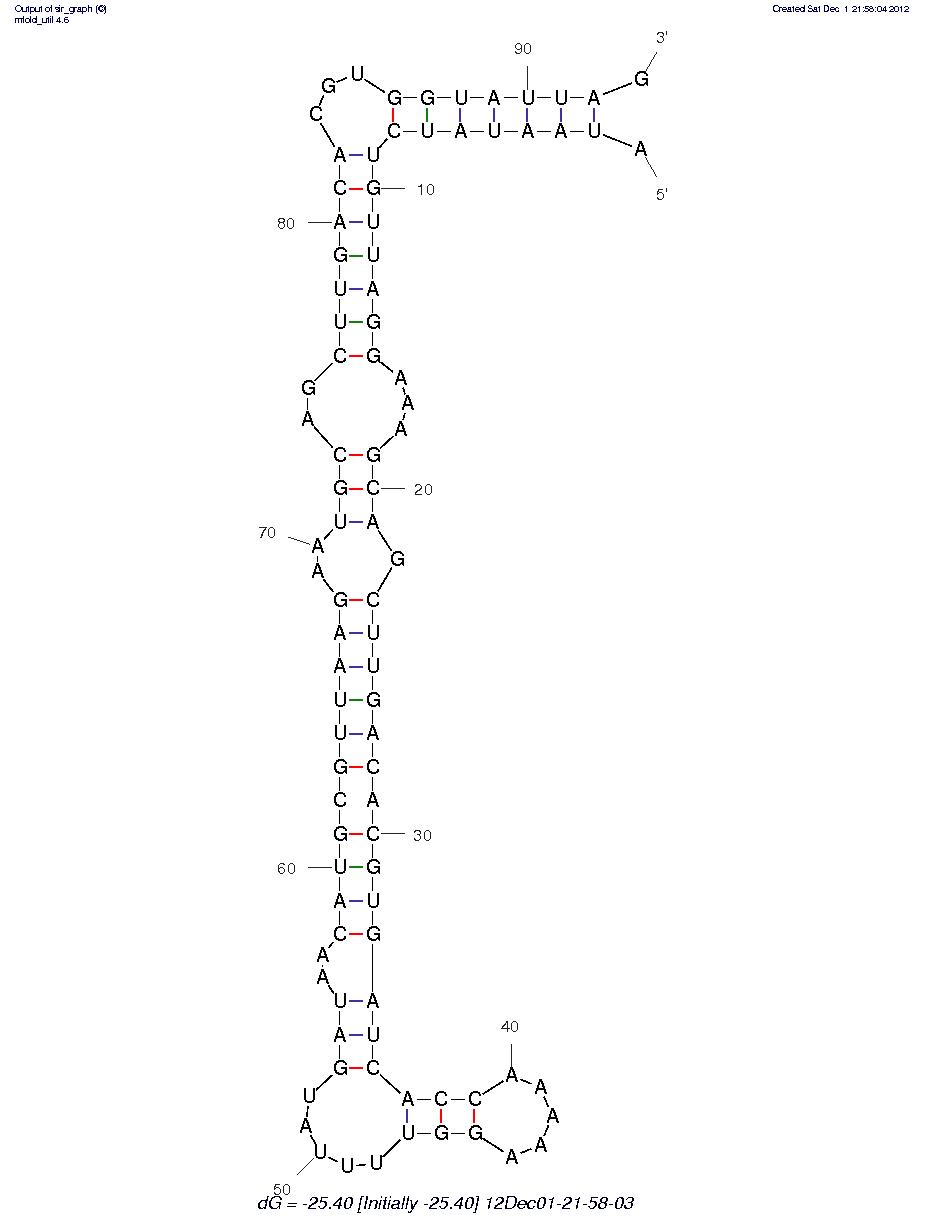

Supplement: Figure S1 — The second structures of newly identified 95 miRNAs including 23 conserved (*) miRNAs and 72 novel pre-miRNAs in castor bean. (DOC) [file pone.0069995.s001.doc]
