# Supplementary material for: Transcriptome-Wide Identification and Characterization of MicroRNAs from Castor Bean (Ricinus communis L.)
Source: PLoS One. 2013 Jul 24;8(7):e69995. doi: 10.1371/journal.pone.0069995 (PMC3722108; doi:10.1371/journal.pone.0069995)

**Figure S2.** Developing seeds of castor bean and lipid (triacylglycerols, TAG) accumulation at two different developmental stages. (**A**) seed1, developing seeds at the initial stage (on 15 days after pollination) were sampled; seed2, developing seeds at the fast oil accumulation stage (on 35 days after pollination) were sampled. (**B**) Thin layer chromatography analysis of TAGs isolated for seed1 and seed2, respectively.


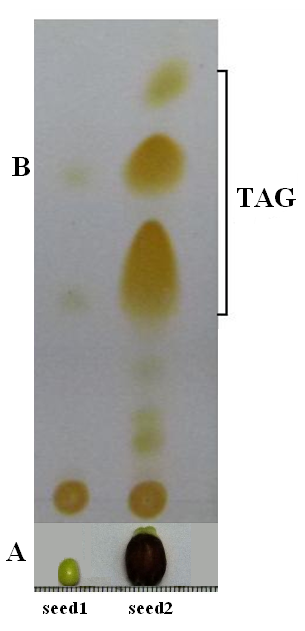

Supplement: Figure S2 — Developing seeds of castor bean and lipid (triacylglycerols, TAG) accumulation at two different developmental stages. (DOC) [file pone.0069995.s002.doc]
