# Supplementary material for: Transcriptome-Wide Identification and Characterization of MicroRNAs from Castor Bean (Ricinus communis L.)
Source: PLoS One. 2013 Jul 24;8(7):e69995. doi: 10.1371/journal.pone.0069995 (PMC3722108; doi:10.1371/journal.pone.0069995)
Supplement: Table S1 — The conserved miRNAs identified from castor bean and their distribution among miRNA families. (DOC) [file pone.0069995.s003.doc]

**Table S1.** The conserved miRNAs identified from castor bean and their distribution among miRNA families.

| miRNA family | leaf | root | seed1 | seed2 | endosperm | Total | Zeng et al.’s study |
| --- | --- | --- | --- | --- | --- | --- | --- |
| miR156/157 | 8 | 8 | 8 | 8 | 8 | 8 | 8 |
| miR159/319 | 4 | 5 | 5 | 5 | 5 | 5 | 5 |
| miR160 | 3 | 2 | 3 | 3 | 3 | 3 | 3 |
| miR162 | 1 | 1 | 1 | 1 | 1 | 1 | 1 |
| miR164 | 4 | 4 | 4 | 4 | 4 | 4 | 4 |
| miR166 | 5 | 5 | 5 | 5 | 5 | 5 | 5 |
| miR167 | 4 | 4 | 4 | 4 | 4 | 4 | 3 |
| miR168 | 1 | 1 | 1 | 1 | 1 | 1 | 1 |
| miR169 | 3 | 8 | 3 | 6 | 10 | 12 | 13 |
| miR170/171 | 9 | 9 | 8 | 9 | 8 | 9 | 8 |
| miR172 | 3 | 4 | 4 | 4 | 4 | 4 | 4 |
| miR390 | 2 | 2 | 2 | 2 | 2 | 2 | 2 |
| miR393 | 2 | 2 | 2 | 2 | 2 | 2 | 2 |
| miR394 | 2 | 2 | 2 | 2 | 2 | 2 | 0 |
| miR395 | 5 | 5 | 5 | 5 | 5 | 5 | 5 |
| miR396 | 3 | 3 | 3 | 3 | 3 | 3 | 3 |
| miR397 | 1 | 1 | 1 | 1 | 1 | 1 | 1 |
| miR398 | 2 | 1 | 2 | 2 | 2 | 2 | 2 |
| miR399 | 3 | 5 | 5 | 5 | 4 | 5 | 9 |
| miR403 | 2 | 2 | 2 | 2 | 2 | 2 | 2 |
| miR408 | 1 | 1 | 1 | 1 | 1 | 1 | 1 |
| miR482 | 1 | 1 | 1 | 1 | 1 | 1 | 0 |
| miR535 | 1 | 1 | 1 | 1 | 1 | 1 | 1 |
| miR827 | 1 | 1 | 1 | 1 | 1 | 1 | 0 |
| miR2111 | 1 | 0 | 1 | 1 | 1 | 1 | 0 |
| miR4414 | 1 | 1 | 1 | 1 | 1 | 1 | 0 |
| Total | 73 | 79 | 76 | 80 | 82 | 86 | 83 |
